# Sesquiterpene Lactones Containing an α-Methylene-γ-Lactone Moiety Selectively Down-Regulate the Expression of Tumor Necrosis Factor Receptor 1 by Promoting Its Ectodomain Shedding in Human Lung Adenocarcinoma A549 Cells

**DOI:** 10.3390/molecules29081866

**Published:** 2024-04-19

**Authors:** Quy Van Vu, Shinsei Sayama, Masayoshi Ando, Takao Kataoka

**Affiliations:** 1 Department of Applied Biology, Kyoto Institute of Technology, Matsugasaki, Sakyo-ku, Kyoto 606-8585, Japan; 2 Department of Natural Sciences (Chemistry), Fukushima Medical University, 1 Hikarigaoka, Fukushima 960-1295, Japan; pbtbw009@yahoo.co.jp; 3 Department of Chemistry and Chemical Engineering, Niigata University, 2-8050 Ikarashi, Nishi-ku, Niigata 950-2181, Japan; 4 Biomedical Research Center, Kyoto Institute of Technology, Matsugasaki, Sakyo-ku, Kyoto 606-8585, Japan

**Keywords:** sesquiterpene, alantolactone, eudesmanolides, perthenolide, costunolide, α-methylene-γ-lactone, tumor necrosis factor receptor 1, ectodomain shedding, nuclear factor κB

## Abstract

Alantolactone is a eudesmane-type sesquiterpene lactone containing an α-methylene-γ-lactone moiety. Previous studies showed that alantolactone inhibits the nuclear factor κB (NF-κB) signaling pathway by targeting the inhibitor of NF-κB (IκB) kinase. However, in the present study, we demonstrated that alantolactone selectively down-regulated the expression of tumor necrosis factor (TNF) receptor 1 (TNF-R1) in human lung adenocarcinoma A549 cells. Alantolactone did not affect the expression of three adaptor proteins recruited to TNF-R1. The down-regulation of TNF-R1 expression by alantolactone was suppressed by an inhibitor of TNF-α-converting enzyme. Alantolactone increased the soluble forms of TNF-R1 that were released into the culture medium as an ectodomain. The structure–activity relationship of eight eudesmane derivatives revealed that an α-methylene-γ-lactone moiety was needed to promote TNF-R1 ectodomain shedding. In addition, parthenolide and costunolide, two sesquiterpene lactones with an α-methylene-γ-lactone moiety, increased the amount of soluble TNF-R1. Therefore, the present results demonstrate that sesquiterpene lactones with an α-methylene-γ-lactone moiety can down-regulate the expression of TNF-R1 by promoting its ectodomain shedding in A549 cells.

## 1. Introduction

The tumor necrosis factor (TNF) cytokine family of ligands and their receptors regulate a wide variety of immune and inflammatory responses [1]. TNF-α is produced by macrophages and other cell types, including adipocytes and endothelial cells [2,3]. TNF-α binds to two distinct receptors: TNF receptor 1 (TNF-R1) and TNF receptor 2 (TNF-R2) [4,5]. In contrast to the restricted expression of TNF-R2, TNF-R1 is ubiquitously expressed in many tissues and plays an important role in the TNF-α-dependent inflammatory response [4,5]. Stimulation with TNF-α induces multiple cellular signaling pathways, including the nuclear factor κB (NF-κB) transcription factor signaling pathway [6,7]. Upon binding to TNF-α, TNF-R1 interacts with TNF-associated death domain protein (TRADD) at its cytoplasmic death domain, which, in turn, recruits adaptor proteins, such as receptor-interacting protein kinase 1 (RIPK1) and TNF receptor-associated factor 2 (TRAF2), to form a membrane-proximal multiprotein complex [6,7]. The TNF-R1 complex induces the activation of inhibitor of NF-κB (IκB) kinase, which phosphorylates IκB, leading to its degradation by the ubiquitin–proteasome system [8]. NF-κB subunits are sequestered in the cytoplasm by IκB, and its degradation allows NF-κB subunits to translocate to the nucleus and subsequently induce the transcription of many target genes [9,10].

TNF-α-converting enzyme (TACE), also known as a disintegrin and metalloproteinase 17 (ADAM17), is widely expressed in various somatic tissues [11,12]. It cleaves many membrane-anchored proteins, including TNF-α, TNF-R1, and TNF-R2, releasing their ectodomains into the extracellular space [13]. TACE-deficient mice exhibit embryonic lethality [14], whereas conditional TACE deficiency in leukocytes, monocytes, and granulocytes confers protection against endotoxin shock [15]. TACE-dependent shedding converts transmembrane forms of TNF-α to their soluble forms, allowing their systemic circulation and subsequent binding to receptors in order to induce cellular signaling pathways [16,17]. Conversely, TACE-dependent ectodomain shedding in responder cells decreases their TNF-α reactivity by down-regulating cell-surface TNF-R1 and TNF-R2 [18]. Therefore, TACE is considered to play an essential role in TNF-α production and cell-surface TNF receptor levels.

Sesquiterpene lactones are secondary metabolites that are found in many plants and exert various biological effects, such as anti-inflammatory activity [19,20]. Sesquiterpene lactones are a group of 15-carbon skeletons with a lactone ring and are divided into several subtypes, including eudesmanolides (e.g., alantolactone) and germacranolides (e.g., parthenolide and costunolide) [19,20]. The biological activities of sesquiterpene lactones are mainly attributed to an α-methylene-γ-lactone moiety, which mainly reacts with the thiol groups of proteins [21]. Compounds with an α,β-unsaturated carbonyl moiety or α-methylene-γ-lactone moiety are known to form covalent bonds with the thiol groups of various proteins, including IκB kinases, in the NF-κB signaling pathway [22,23,24].

**Figure 1:**
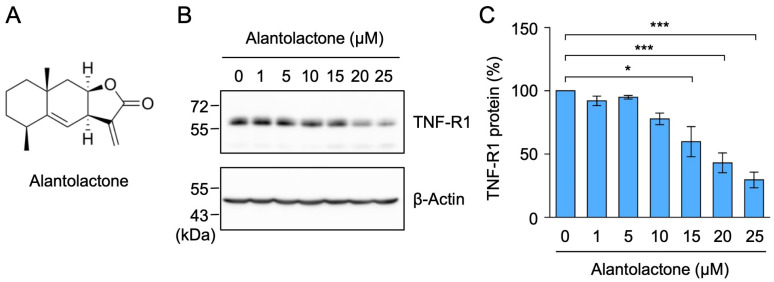
Alantolactone down-regulated the expression of TNF-R1 in A549 cells. (**A**) Structure of alantolactone. (**B**,**C**) A549 cells were treated with alantolactone for 1 h at the concentrations indicated in the figure panels. Western blotting was used to assess the protein levels in the cell lysates. Blots are representative of three independent experiments (**B**). The amount of TNF-R1 protein was normalized to the amount of β-actin protein. The level of TNF-R1 protein (%) (**C**) is shown as the mean ± standard error of three independent experiments * *p* < 0.05 and *** *p* < 0.001. Original blots are shown in Supplementary Figures S1–S4.

Alantolactone is a eudesmane-type sesquiterpene lactone that possesses an α-methylene-γ-lactone moiety (Figure 1A). Previous studies demonstrated that alantolactone interfered with the NF-κB signaling pathway’s anti-inflammatory and anticancer activities [25,26,27]. Furthermore, alantolactone was shown to inhibit TNF-α-dependent IκBα phosphorylation in human leukemia cells [28]. As one of the cellular molecular targets, alantolactone has been reported to inhibit IκB kinase activity by interacting with the ATP-binding site [29]. Consistent with this finding, we recently showed that alantolactone inhibited the TNF-α-induced phosphorylation and degradation of the IκBα protein, whereas alantolactone derivatives without an α-methylene-γ-lactone moiety suppressed the binding of NF-κB subunits to DNA downstream of IκB kinase activation [30]. These findings suggest the potential of alantolactone to target multiple steps in the NF-κB signaling pathway.

In the present study, we investigated the mechanism of action of alantolactone and unexpectedly found that it down-regulated TNF-R1 expression without affecting the expression of adaptor proteins in human adenocarcinoma A549 cells. Using additional sesquiterpene eudesmanolide and germacranolide lactones, the results obtained herein showed that sesquiterpenes containing an α-methylene-γ-lactone moiety can selectively down-regulate the expression of TNF-R1 by promoting its ectodomain shedding in A549 cells.

## 2. Results

### 2.1. Alantolactone Selectively Down-Regulated the Expression of TNF-R1, but Not TRADD, RIPK1, or TRAF2

We recently reported that alantolactone inhibited the TNF-α-induced phosphorylation of IκBα and its proteasomal degradation in A549 cells [30]. In A549 cells, TNF-R1, but not TNF-R2, was mainly expressed at the mRNA and protein levels, in contrast to human histiocytic lymphoma U937 cells, which were used as the positive control [31]. Upon stimulation with TNF-α, TNF-R1 was shown to form a membrane-proximal complex by associating with TRADD, RIPK1, and TRAF2, which are required for IκB kinase activation [6,7]. To establish whether alantolactone affects the signaling pathway upstream of IκB kinase activation, we examined the expression of these TNF-R1 complex components by Western blotting. When A549 cells were treated with alantolactone for 1 h, TNF-R1 expression decreased in a dose-dependent manner and at 15–25 µM (Figure 1B,C).

**Figure 2:**
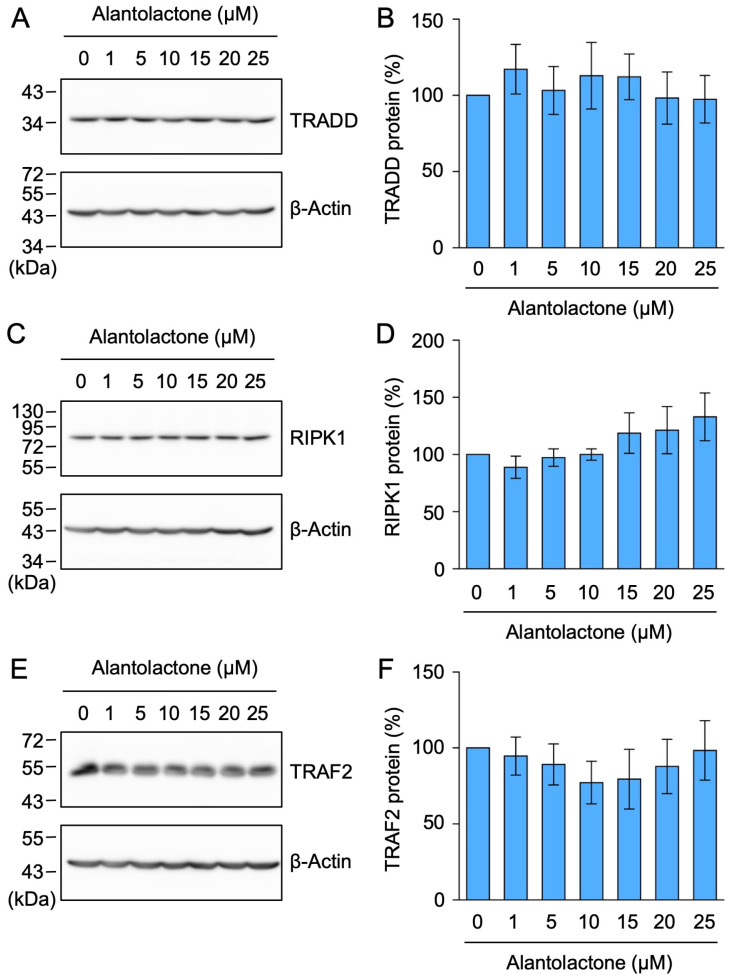
Alantolactone did not affect the expression of TRADD, RIPK1, or TRAF2 in A549 cells. (**A**–**F**) A549 cells were treated with alantolactone for 1 h at the concentrations indicated in the figure panels. Western blotting was used to assess the protein levels in the cell lysates. Blots are representative of three independent experiments (**A**,**C**,**E**). The amount of each protein was normalized to the amount of β-actin protein. The levels of TRADD protein (%) (**B**), RIPK1 protein (%) (**D**), and TRAF2 protein (%) (**F**) are shown as the mean ± standard error of three independent experiments. No significant differences were observed in (**B**,**D**,**F**). Original blots are shown in Figures S5–S16.

In contrast, the expression of TRADD, RIPK1, and TRAF2 was not markedly affected by alantolactone at concentrations up to 25 µM (Figure 2A–F). These results demonstrate that alantolactone selectively down-regulated the expression of TNF-R1.

### 2.2. Alantolactone Down-Regulated the Expression of Transfected TNF-R1

**Figure 3:**
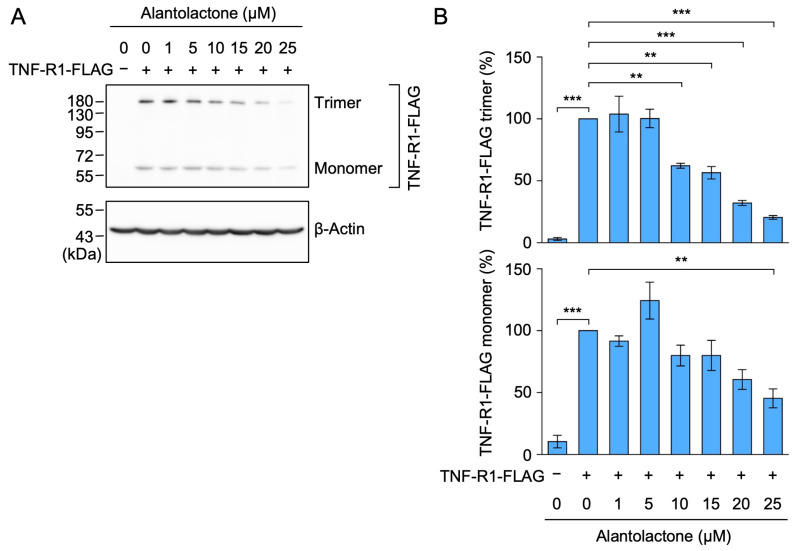
Alantolactone down-regulated the expression of transfected TNF-R1 in A549 cells. (**A**,**B**) A549 cells were left untreated (−) or transfected with an expression vector encoding FLAG-tagged TNF-R1 (+) for 24 h. A549 cells were treated with alantolactone for 1 h at the concentrations indicated in the figure panels. Western blotting was used to assess the protein levels in the cell lysates. Blots are representative of three independent experiments (**A**). The amount of TNF-R1-FLAG protein in the cell lysates was normalized to the amount of β-actin protein. The levels of TNF-R1-FLAG monomers (%) and TNF-R1-FLAG trimers (%) are shown as the mean ± standard error of three independent experiments (**B**). ** *p* < 0.01 and *** *p* < 0.001. Original blots are shown in Figures S17–S20.

We transfected A549 cells with a pCR3 expression vector encoding FLAG-tagged human TNF-R1, the expression of which was driven by a constitutive cytomegalovirus promoter. In contrast to endogenous TNF-R1, the transfected TNF-R1 was detected as monomers (migrating around a 55 kDa marker) and putative trimers (migrating around a 180 kDa marker) by Western blotting using an anti-FLAG antibody (Figure 3A). The treatment with alantolactone for 1 h down-regulated the expression of monomeric TNF-R1 in a dose-dependent manner (Figure 3A,B). Consistent with our previous study using A549 cells and human embryonic kidney 293T cells [32], putative trimeric TNF-R1 was resistant to thiol-reducing agents, suggesting that they are not linked by disulfide bonds. Alantolactone also reduced the amount of the trimeric TNF-R1 bands (Figure 3A,B). We previously showed that the expression of TNF-R1 in A549 cells did not decrease following a 1 h exposure to the translation inhibitor cycloheximide [31]. Therefore, the down-regulation of TNF-R1 by alantolactone may not be due to a reduction in endogenous transcription or global translation.

### 2.3. The TACE Inhibitor Reversed the Down-Regulation of TNF-R1 Expression by Alantolactone in A549 Cells

**Figure 4:**
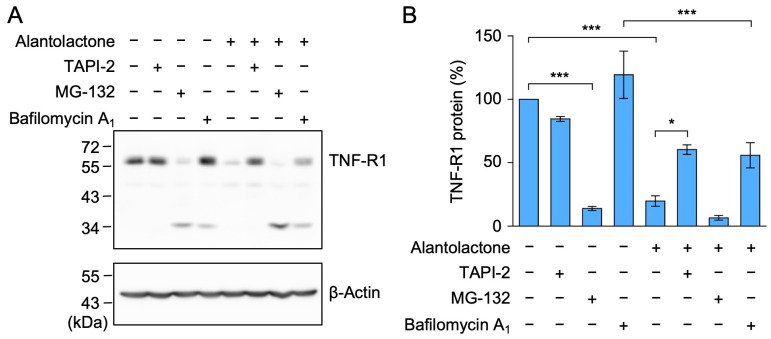
TAPI-2 suppressed the down-regulation of TNF-R1 expression by alantolactone in A549 cells. (**A**,**B**) A549 cells were pretreated without (−) or with (+) TAPI-2, MG-132, or bafilomycin A_1_ for 1 h, and were then treated without (−) or with (+) alantolactone (25 µM) in the absence (−) or presence (+) of TAPI-2 (25 µM), MG-132 (20 µM), or bafilomycin A_1_ (100 nM) for 1 h at the indicated final concentrations. Blots are representative of three independent experiments (**A**). The amount of TNF-R1 protein was normalized to the amount of β-actin protein. The levels of TNF-R1 protein (%) in the cell lysates are shown as the mean ± standard error of three independent experiments (**B**). * *p* < 0.05 and *** *p* < 0.001. Original blots are shown in Figures S21–S24.

To investigate whether alantolactone induced the proteolytic degradation of TNF-R1, A549 cells were preincubated with TNF-α protease inhibitor 2 (TAPI-2) (a TACE inhibitor), MG-132 (a proteasome inhibitor), or bafilomycin A_1_ (an inhibitor of vacuolar-type H^+^-ATPases), followed by a 1 h treatment with alantolactone. TAPI-2 suppressed the reduction in TNF-R1 by alantolactone (Figure 4A,B). In the presence of bafilomycin A_1_, TNF-R1 expression was still reduced by alantolactone (Figure 4A,B). MG-132 alone reduced TNF-R1, and this was accompanied by cleavage products (Figure 4A,B), which is consistent with our previous findings [33]. This unexpected effect of MG-132 may not be directly related to the effects of alantolactone; however, further studies are needed to examine the regulation of TNF-R1 expression by proteasomal degradation. Of the three inhibitors tested, TAPI-2 appeared to be the most effective at preventing the down-regulation of TNF-R1 by alantolactone.

### 2.4. Alantolactone Promoted the Ectodomain Shedding of TNF-R1 in A549 Cells

**Figure 5:**
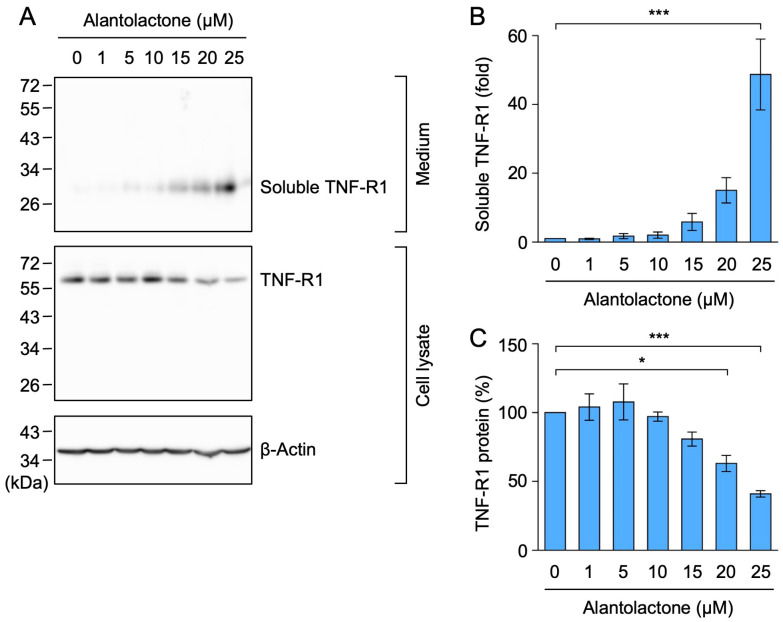
Alantolactone promoted the ectodomain shedding of TNF-R1 in A549 cells. (**A**–**C**) A549 cells were treated with alantolactone for 1 h at the concentrations indicated in the figure panels. Blots are representative of three independent experiments (**A**). The amount of TNF-R1 protein was normalized to the amount of β-actin protein. The levels of soluble TNF-R1 protein (fold) (**B**) in the culture medium and TNF-R1 protein (%) (**C**) in the cell lysates are shown as the mean ± standard error of three independent experiments. * *p* < 0.05 and *** *p* < 0.001. Original blots are shown in Figures S25–S28.

Cell-surface TNF-R1 is a TACE substrate, and its ectodomain is released as soluble forms after cleavage [13]. A549 cells were treated with alantolactone for 1 h, and the amount of TNF-R1 in the culture medium and cell lysates was assessed by Western blotting. Alantolactone increased the amount of TNF-R1 (migrating between 26 and 34 kDa markers) in the culture medium in a dose-dependent manner (Figure 5A,B). Conversely, alantolactone decreased the amount of TNF-R1 in the cell lysates (Figure 5A,C). These results showed that alantolactone promoted the ectodomain shedding of TNF-R1.

### 2.5. The Down-Regulation of TNF-R1 Expression by Alantolactone Was Suppressed by Glutathione, N-Acetyl-L-Cysteine (NAC), and L-Cysteine

**Figure 6:**
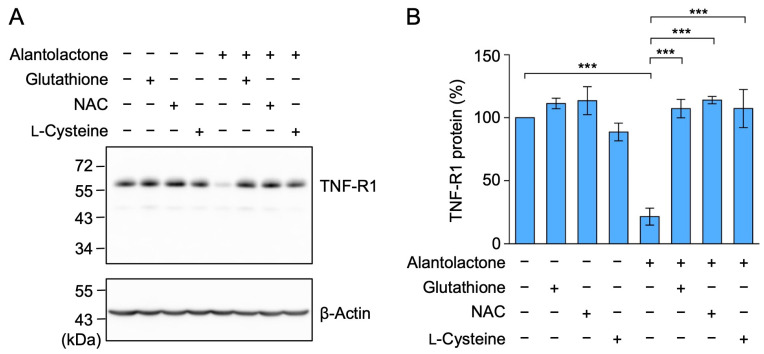
TNF-R1 shedding promoted by alantolactone was suppressed by glutathione, NAC, and L-cysteine in A549 cells. (**A**,**B**) A549 cells were pretreated without (−) or with (+) glutathione, NAC, and L-cysteine for 1 h, and were then treated without (−) or with (+) alantolactone (25 µM) in the absence (−) or presence (+) of glutathione (10 mM), NAC (10 mM), and L-cysteine (2 mM) at the indicated final concentrations for 1 h (**A**,**B**). Blots are representative of three independent experiments (**A**). The amount of TNF-R1 protein was normalized to the amount of β-actin protein. The level of TNF-R1 protein (%) in the cell lysates is shown as the mean ± standard error of three independent experiments (**B**). *** *p* < 0.001. Original blots are shown in Figures S29–S32.

An α-methylene-γ-lactone moiety reacts with the thiol groups of proteins via the Michael addition reaction [22,23,24]. To clarify whether the α-methylene-γ-lactone moiety of alantolactone is critical for down-regulating the expression of TNF-R1, A549 cells were pretreated with glutathione, NAC, and L-cysteine, all of which contain a thiol group, and were then treated with alantolactone. Glutathione, NAC, and L-cysteine did not affect the expression of TNF-R1 by themselves, but they did attenuate the reduction in TNF-R1 protein expression by alantolactone (Figure 6A,B). These results demonstrated that the α-methylene-γ-lactone moiety of alantolactone is necessary for the down-regulation of TNF-R1 expression.

### 2.6. Structure–Activity Relationship of Eudesmane Derivatives: The α-Methylene-γ-Lactone Moiety of Eudesmane Derivatives Was Necessary to Promote the Ectodomain Shedding of TNF-R1

**Figure 7:**
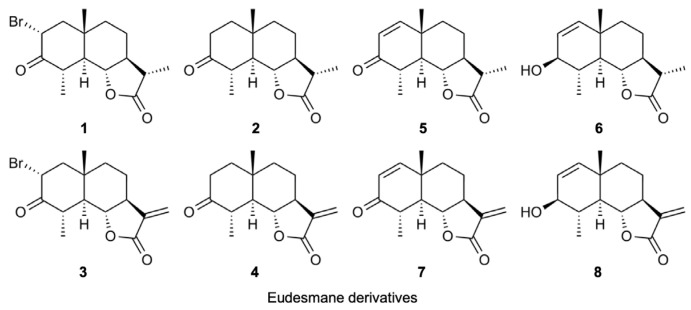
Structures of eudesmane derivatives **1**–**8**.

We previously showed that eudesmane-type sesquiterpene lactones targeted multiple steps in the NF-κB signaling pathway induced by TNF-α and IL-1α [34]. Eudesmane derivatives **1**–**8** are characterized by the presence or absence of an α-bromo ketone moiety, α,β-unsaturated carbonyl moiety, or α-methylene-γ-lactone moiety (Figure 7). Among the eight eudesmane derivatives **1**–**8**, we previously showed that **3**, **4**, and **7** inhibited TNF-α-induced IκBα phosphorylation and subsequent degradation in A549 cells [34].

**Figure 8:**
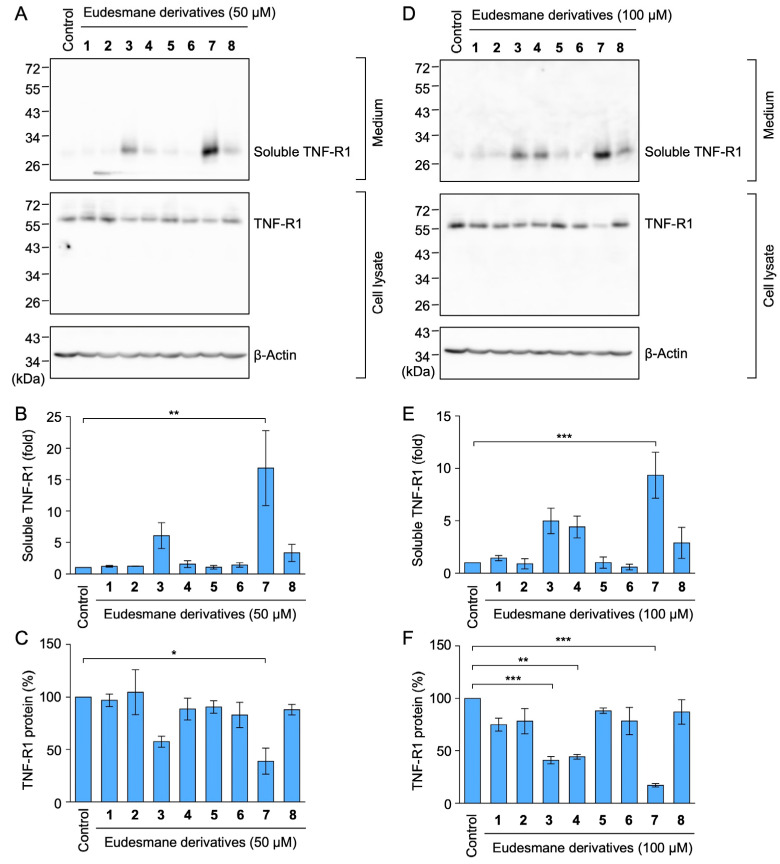
Eudesmane derivatives with an α-methylene-γ-lactone moiety promoted TNF-R1 shedding in A549 cells. (**A**–**F**) A549 cells were treated without (Control) or with **1**–**8** at 50 µM (**A**–**C**) or 100 µM (**D**–**F**) for 1 h at the indicated final concentrations. Blots are representative of three independent experiments (**A**,**D**). The amount of TNF-R1 protein was normalized to the amount of β-actin protein. The levels of soluble TNF-R1 protein (fold) in the culture medium (**B**,**E**) and TNF-R1 protein (%) in the cell lysates (**C**,**F**) are shown as the mean ± standard error of three independent experiments. * *p* < 0.05, ** *p* < 0.01 and *** *p* < 0.001. Original blots are shown in Figures S33–S40.

Based on these findings, we investigated whether eudesmane derivatives affected TNF-R1 expression. The amount of soluble TNF-R1 increased when A549 cells were treated with **7** at 50 µM and **3**, **4**, and **7** at 100 µM (Figure 8A,B,D,E). The TNF-R1 expression in cell lysates was also decreased by **3**, **4**, and **7** at 100 µM (Figure 8A,C,D,F). Although **8** contains an α-methylene-γ-lactone moiety, **8** at 50–100 µM did not affect the amount of soluble TNF-R1 or TNF-R1 in cell lysates (Figure 8A–F). These results suggest that an α-methylene-γ-lactone moiety in eudesmane derivatives was necessary to promote the ectodomain shedding of TNF-R1.

### 2.7. Parthenolide and Costunolide Promoted the Ectodomain Shedding of TNF-R1

**Figure 9:**
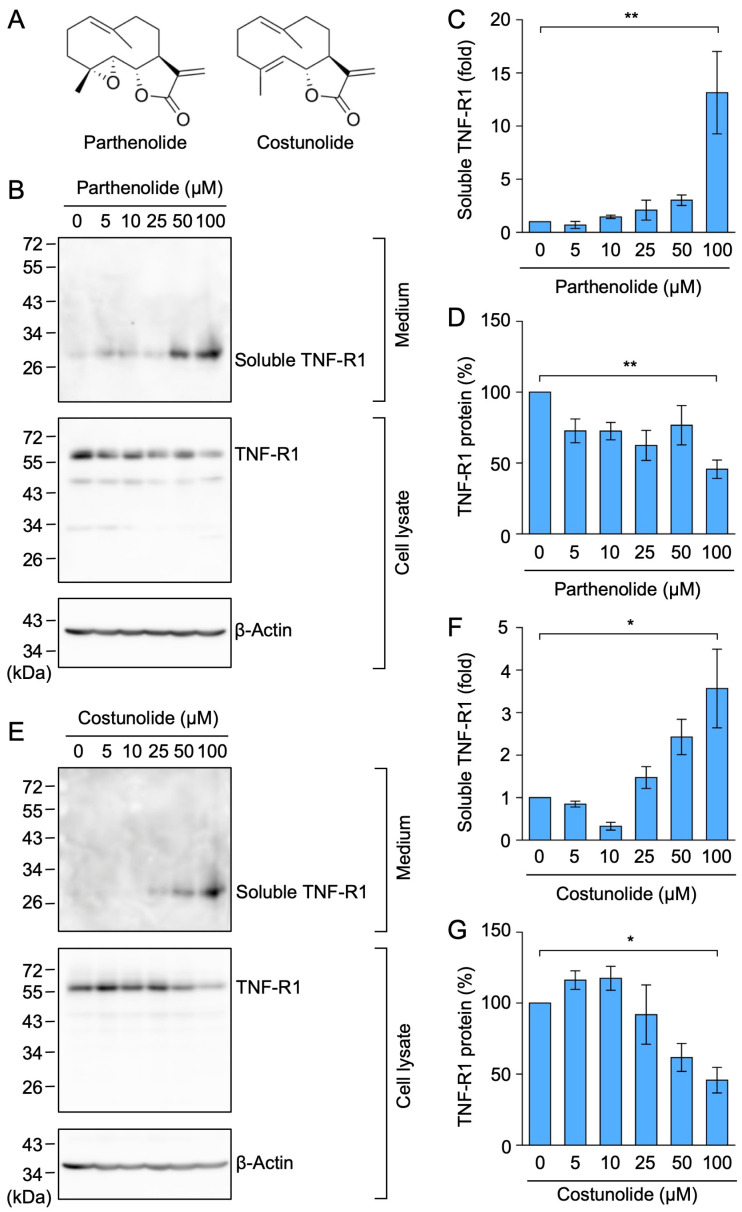
Parthenolide and costunolide promoted TNF-R1 shedding in A549 cells. (**A**) Structures of parthenolide and costunolide. (**B**–**G**) A549 cells were treated with parthenolide (**B**–**D**) and costunolide (**E**–**G**) for 1 h at the concentrations indicated in the figure panels. Blots are representative of three independent experiments (**B**,**E**). The amount of TNF-R1 protein was normalized to the amount of β-actin. The levels of soluble TNF-R1 protein (fold) in the culture medium (**C**,**F**) and TNF-R1 protein (%) in the cell lysates (**D**,**G**) are shown as the mean ± standard error of three independent experiments. * *p* < 0.05 and ** *p* < 0.01. Original blots are shown in Figures S41–S48.

Parthenolide and costunolide are germacranolides possessing an α-methylene-γ-lactone moiety (Figure 9A). Parthenolide was previously shown to bind and inhibit IκB kinase β, but not IκB kinase β with a C179A mutation [35]. Costunolide has been reported to inhibit NF-κB activation via covalent binding to cysteine 179 of IκB kinase β [36]. Based on the similar biological activities between germacranolides and eudesmanolides, we hypothesized that parthenolide and costunolide may affect TNF-R1 expression. Parthenolide at 100 µM increased the soluble TNF-R1 level in the culture medium, and decreased the cellular level of TNF-R1 in A549 cells (Figure 9B–D). Similarly, costunolide at 100 µM increased the amount of soluble TNF-R1, but conversely decreased the cellular level of TNF-R1 (Figure 9E–G). These results suggest that parthenolide and costunolide down-regulate TNF-R1 expression by promoting ectodomain shedding.

## 3. Discussion

Previous studies reported that alantolactone inhibited the NF-κB signaling pathway, when it was constitutively activated and induced by different stimuli [25,26,27,37,38,39,40,41,42,43,44,45,46,47]. In the TNF-α-dependent NF-κB signaling pathway, we and others demonstrated that alantolactone inhibited IκBα phosphorylation [28,30]. Alantolactone was also shown to inhibit IκB kinase β by interacting with the ATP-binding site [29]. Since IκB kinase β plays an essential role in the NF-κB signaling pathway [24], IκB kinase β may be a common target protein of alantolactone in the NF-κB signaling pathway. In the present study, we found that TNF-R1 was down-regulated in A549 cells when they were exposed to alantolactone for 1 h. In contrast, alantolactone did not reduce the expression of TRADD, RIPK1, or TRAF2, which are adaptor proteins that are part of the TNF-R1 complex. TNF-R1, TRADD, RIPK1, and TRAF2 appeared to be relatively stable at the protein level because their expression was not affected by a 1 h treatment with the translation inhibitor cycloheximide [31]. Moreover, the down-regulation of TNF-R1 by alantolactone was reversed by the inhibition of TACE by TAPI-2, whereas the inhibition of lysosomal proteolytic degradation by bafilomycin A_1_ was ineffective at blocking the down-regulation of TNF-R1 by alantolactone. Alantolactone promoted an increase in cleaved TNF-R1 as a soluble form in the culture medium. These results clearly indicate that alantolactone down-regulated the expression of TNF-R1 by promoting its ectodomain shedding.

Many sesquiterpene lactones with an α,β-unsaturated carbonyl moiety undergo the Michael addition reaction and covalently bind to the thiol groups of proteins, thereby modulating their biological activities [19,20]. Previous studies reported that sesquiterpene lactones inhibited IκB kinases (e.g., IκB kinase β) and NF-κB subunits (e.g., RelA) by targeting critical cysteines via their α,β-unsaturated carbonyl moiety [22,23,24]. IκB kinase β contains serine 177 and 181 residues in its activation loop, both of which are phosphorylated for activation [48,49]. IκB kinase β also contains cysteine 179 in its activation loop, which is covalently attached to α,β-unsaturated carbonyl compounds, resulting in the blockade of kinase activity [22,23,24]. The NF-κB subunit RelA contains cysteine 38 in the N-terminal Rel homology domain, which is responsible for DNA binding and homo- and heterodimerization [50,51]. Sesquiterpene lactones have been shown to covalently bind to cysteine 38 of RelA via their α,β-unsaturated carbonyl moiety, thereby preventing RelA functions, including its DNA-binding activity [22,23]. In addition, 1β-hydroxyalantolactone was found to directly target the catalytic cysteine residue of the ubiquitin-conjugated enzyme UbcH5, thereby inhibiting TNF-α-induced NF-κB activation [52]. An in silico docking study recently demonstrated that handelin, a guaianolide dimer belonging to the sesquiterpene lactones, inhibited transforming growth factor β-activated kinase 1 (TAK1) by targeting the ATP-binding pocket [53]. So far, these proteins are considered to be the target proteins of sesquiterpene lactones to block the NF-κB signaling pathway. In the present study, we showed that cell-surface TNF-R1 was down-regulated by alantolactone via the promotion of TACE-dependent cleavage. Therefore, alantolactone appears to modulate TNF-R1 levels as an initial step in the TNF-α-dependent NF-κB signaling pathway.

**Figure 10:**
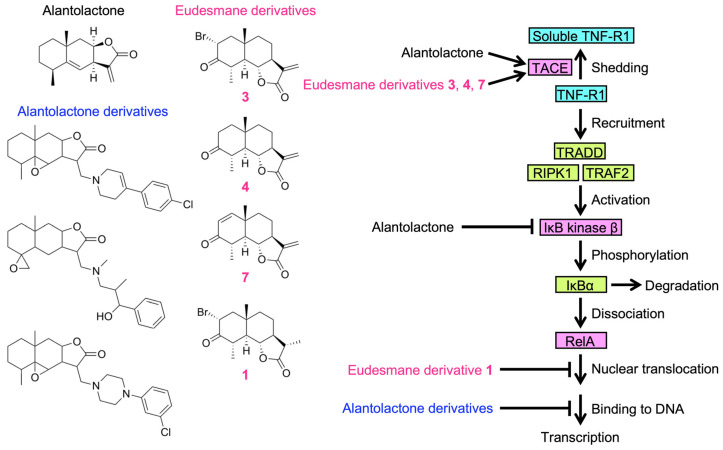
Proposed mechanisms of action of eudesmane-type sesquiterpene lactones on the TNF-α-induced NF-κB signaling pathway. Structures of alantolactone, alantolactone derivatives, and eudesmane derivatives are presented. TNF-R1 is cleaved to its soluble forms by TACE and released into the culture medium. Upon binding to TNF-α, TNF-R1 recruits TRADD, RIPK1, and TRAF2 to form the membrane-proximal complex, which activates IκB kinase β by phosphorylating serines 177 and 181 in the activation loop. In the cytoplasm, phosphorylated IκBα is ubiquitinated and degraded by the proteasome. Upon dissociation from IκBα, NF-κB subunits, including RelA, translocate to the nucleus and bind to the κB sites of target genes, initiating their transcription. Based on the present and previous studies, the mechanisms of action of eudesmane-type sesquiterpene lactones on the TNF-α-induced NF-κB signaling pathway are proposed as follows: (1) Alantolactone promotes the TACE-dependent shedding of TNF-R1, thereby down-regulating the expression of cell-surface TNF-R1 (this study). (2) Alantolactone inhibits IκB kinase β by interacting with the ATP-binding site [29]. (3) Three alantolactone derivatives inhibit RelA binding to DNA, but not its nuclear translocation [30]. (4) Eudesmane derivatives **3**, **4**, and **7** promote the ectodomain shedding of TNF-R1 and thereby down-regulate the expression of cell-surface TNF-R1 (this study). (5) Eudesmane derivative **1** inhibits RelA translocation to the nucleus [54].

We recently reported that the inhibitory activity of alantolactone was attenuated by glutathione, NAC, and L-cysteine, which are thiol-containing compounds [30], indicating that the α-methylene-γ-lactone moiety of alantolactone is essential for its inhibitory activity. Nevertheless, alantolactone derivatives lacking an α-methylene-γ-lactone moiety, but containing an epoxide moiety, inhibited the TNF-α-induced NF-κB signaling pathway by a different mechanism from that of alantolactone; alantolactone derivatives did not affect TNF-α-induced IκBα phosphorylation and degradation or nuclear RelA translocation, but inhibited RelA binding to the promoter region of its target gene [30]. Therefore, the structural modification of alantolactone revealed potential hidden targets downstream of IκB kinase β activation. Based on our present and previous studies, alantolactone and its derivatives are postulated to target at least two different steps in the TNF-α-dependent NF-κB signaling pathway: TNF-R1 expression and DNA binding of the NF-κB subunit RelA (Figure 10).

Among the sesquiterpene lactones structurally related to alantolactone, we previously reported the structure–activity relationship of eight eudesmane derivatives, which contain either an α-bromo ketone moiety or α,β-unsaturated carbonyl moiety in the A-ring and/or an α-methylene-γ-lactone moiety in the C-ring [34]. Among eudesmane derivatives **1**–**8**, **3**, **4**, and **7** inhibited TNF-α-induced IκBα phosphorylation, whereas the effects of **1**, **2**, **5**, **6**, and **8** were negligible [34]. Consistent with our previous findings, **3**, **4**, and **7** induced the ectodomain shedding of TNF-R1, whereas **1**, **2**, **5**, **6**, and **8** were ineffective. These results suggest that an α-bromo ketone moiety in the A-ring (found in **1** and **3**) and an α,β-unsaturated carbonyl moiety in the A-ring (found in **5** and **7**) are not necessarily important for promoting TNF-R1 shedding. In contrast, an α-methylene-γ-lactone moiety in the C-ring, which is common to **3**, **4**, and **7**, was necessary for promoting TNF-R1 shedding, with the exception of **8**, in which a hydroxy group in the A-ring may influence the inhibitory activity. We previously showed that **1** did not prevent TNF-α-induced IκB phosphorylation, but inhibited the TNF-α-induced nuclear translocation of wild-type RelA [34,54]. In contrast to wild-type RelA, **1** did not affect the TNF-α-induced translocation of the RelA mutant in which cysteine 38 was replaced by a serine [54]. Based on our previous findings and the present results, **1** with an α-bromo ketone moiety may selectively inhibit RelA in the TNF-α-induced NF-κB signaling pathway, whereas **3**, **4**, and **7** with an α-methylene-γ-lactone moiety modulated the expression of TNF-R1 in the initial step of the TNF-α-dependent NF-κB signaling pathway (Figure 10).

Germacranolides, such as parthenolide and costunolide, have been reported to inhibit multiple steps of the NF-κB signaling pathway [19,20]. Parthenolide was shown to directly bind to and inhibit IκB kinase β and this inhibitory activity was abolished by a mutation in cysteine 179 [35]. Costunolide was found to covalently bind to cysteine 179 of IκB kinase β using a biotinylated costunolide as a purification tool [36]. In addition, parthenolide and costunolide target other cellular proteins, including RelA, in the NF-κB signaling pathway [55,56,57,58]. Due to the similarity of their biological activities to eudesmanolides, we investigated whether parthenolide and costunolide down-regulated TNF-R1 expression by promoting its ectodomain shedding. Parthenolide and costunolide promoted the ectodomain shedding of TNF-R1. Therefore, the present results support the notion that sesquiterpene lactones with an α-methylene-γ-moiety are capable of promoting the ectodomain shedding of TNF-R1. TNF-R1 is ubiquitously expressed in the majority of cells [4,5], whereas TACE is also widely expressed in various tissues [11,12]. Therefore, the down-regulation of cell-surface TNF-R1 expression may contribute to the inhibitory effects of sesquiterpene lactones on the TNF-α-induced signaling pathway.

TACE is a membrane-anchored metalloproteinase that consists of multiple domains, including a prodomain, catalytic domain, transmembrane domain, and cytoplasmic tail [11,13]. It is synthesized in an inactive form and its prodomain is removed in the trans-Golgi network [12]. Mitogen-activated protein (MAP) kinases, including extracellular signal-regulated kinase (ERK) and p38 MAP kinase, are activated by phosphorylation through upstream kinase cascades in response to various stimuli and regulate many cellular responses [59]. As a form of post-translational regulation, TACE is phosphorylated in its cytoplasmic tail by protein kinases, such as ERK and p38 MAP kinase [13]. ERK and p38 MAP kinase have been shown to phosphorylate TACE at threonine 735 in the cytoplasmic tail [60,61,62,63]. Furthermore, the catalytic activity of TACE and its trafficking to the cell surface are regulated by the phosphorylation by ERK and p38 MAP kinase [60,61,62,63]. Several types of translation inhibitors have been shown to induce the activation of MAP kinases via the ribotoxic stress response [64]. We previously reported that glutarimide and triene-ansamycin translation inhibitors rapidly induced the ectodomain shedding of TNF-R1, thereby preventing TNF-α-induced NF-κB activation, which was reversed by inhibitors of the ERK and p38 MAP kinase pathways in A549 cells [65]. In addition to its inhibitory effects on ERK and/or p38 MAP kinase [37,44,66], alantolactone has been shown to increase the phosphorylation of ERK and p38 MAP kinase in human breast, gastric, and colon cancer cells [41,42,67,68]. Therefore, ERK and p38 MAP kinase appear to be involved in the ectodomain shedding of TNF-R1 promoted by alantolactone.

## 4. Materials and Methods

### 4.1. Cells

Human lung adenocarcinoma A549 cells (JCRB0076) were obtained from the National Institutes of Biomedical Innovation, Health, and Nutrition JCRB Cell Bank (Osaka, Japan). A549 cells were maintained by subculturing every 2–3 days in RPMI 1640 medium (Thermo Fisher Scientific, Waltham, MA, USA) supplemented with fetal calf serum (FCS) (Sigma-Aldrich, St. Louis, MO, USA), which was treated at 56 °C for 30 min, and a Penicillin-Streptomycin Mixed Solution (Stabilized) (Nacalai Tesque, Kyoto, Japan).

### 4.2. Reagents

Alantolactone (catalog number: S8318; Selleck Chemicals, Houston, TX, USA), bafilomycin A_1_ (item number: 119038; Cayman Chemical, Ann Arbor, MI, USA), costunolide (product number: 032-13731; Wako Pure Chemical Industries, Osaka, Japan), glutathione (product number: 073-02013; FUJIFILM Wako Pure Chemical Corporation, Osaka, Japan), L-cysteine (product number: 10309-41: Nacalai Tesque), Z-Leu-Leu-Leu-H (aldehyde) (also known as MG-132) (code number: 3175-v; Peptide Institute, Osaka, Japan), NAC (product number: 00512-84; Nacalai Tesque), parthenolide (catalog number: P0667-5MG; Sigma, St. Louis, MO, USA), and TAPI-2 (code number: INH-3852-PI; Peptide Institute) were purchased as commercial products. (11*S*)-2α-bromo-3-oxoeudesmano-12,6α-lactone (also known as santonin-related compound 2: SRC2) (**1**), (11*S*)-3-oxoeudesmano-12,6α-lactone (**2**), 2α-bromo-3-oxoeudesm-11(13)-eno-12,6α-lactone (**3**), 3-oxoeudesm-11(13)-eno-12,6α-lactone (**4**), (11*S*)-3-oxoeudesm-1-eno-12,6α-lactone (**5**), (11*S*)-3β-hydroxyeudesm-1-eno-12,6α-lactone (**6**), 3-oxoeudesma-1,11(13)-dieno-12,6α-lactone (also known as (+)-tuberiferin) (**7**), and 3β-hydroxyeudesma-1,11(13)-dieno-12,6α-lactone (**8**) were synthesized as previously described [36,69,70].

### 4.3. Antibodies

Antibodies reactive to β-actin (clone: AC-15, catalog number: A5441; Sigma-Aldrich), the DYKDDDDK tag (FLAG) (clone: 1E6, code number: 012-22384; Fujifilm Wako Pure Chemical Corporation), RIPK1 (clone: 38/RIP, material number: 610458; BD Biosciences, San Jose, CA, USA), TNF-R1 (H-5, catalog number: sc-8436; Santa Cruz Biotechnology, Dallas, TX, USA), TNF-R1 (C25C1, catalog number: #3736; Cell Signaling Technology, Danvers, MA, USA), TRADD (clone: 37/TRADD, material number: 610572; BD Biosciences), and TRAF2 (F-2, catalog number: sc-136999; Santa Cruz Biotechnology) were used as primary antibodies for Western blotting. A peroxidase-conjugated goat anti-mouse IgG (H + L) antibody (code number: 115-035-146) and peroxidase-conjugated goat anti-rabbit IgG (H + L) antibody (code number: 111-035-144) were obtained from Jackson ImmunoResearch Laboratories (West Groove, PA, USA), which were used as secondary antibodies for Western blotting.

### 4.4. Expression Vectors

The pCR3 expression vector encoding human full-length TNF-R1 fused to a C-terminal FLAG tag was described previously [32].

### 4.5. Preparation of Cell Lysates and Medium Fractions

A549 cells were dispersed on 35 mm dishes (5 × 10^5^ cells/dish, 1.5 mL) and precultured overnight. The culture medium was removed prior to experiments, and cells were treated in a final volume of 1 mL. To analyze the culture medium, FCS-free RPMI 1640 medium was used. The culture medium was harvested and then centrifuged (15,300× *g*, 5 min) to remove cell debris and insoluble materials. Supernatants were collected as medium fractions. Equal amounts of medium fractions (0.5 mL) were treated with 0.625 mL of chloroform/methanol (1:4) and centrifuged (15,300× *g*, 5 min) to collect protein precipitates. After the removal of the culture medium and its replacement with PBS, A549 cells were detached by scrapers and collected by centrifugation (800× *g*, 5 min). Cells were lysed with 1% Triton X-100 lysis buffer (1% Triton X-100, 50 mM Tris-HCl (pH 7.4), 2 mM sodium vanadate, and 2 mM dithiothreitol) supplemented with cOmplete^TM^ (Sigma-Aldrich) protease inhibitors on ice for 15 min. Cell lysates were collected as supernatants after centrifugation (15,300× *g*, 5 min). Protein concentrations were measured using the Protein Assay CBB Solution (5×) (Nacalai Tesque).

### 4.6. Western Blotting

Equal amounts of cell lysates and medium fractions were applied to sodium dodecyl sulfate–polyacrylamide gels and then separated by electrophoresis. Proteins were transferred from the gels to ClearTrans^®^ Nitrocellulose Membranes, 0.2 µm (Fujifilm Wako Pure Chemical Corporation) using the Mini Trans-Blot^®^ Cell Transfer System (Bio-Rad Laboratories, Hercules, CA, USA). The nitrocellulose membranes were incubated with 0.5% Tween 20–PBS supplemented with 5% skim milk at 4 °C overnight for blocking. The membranes were reacted with primary antibodies and secondary antibodies in 0.5% Tween 20–PBS supplemented with 5% skim milk for 1 h. To wash the membranes after the antibody reaction, the membranes were treated several times with 0.5% Tween 20–PBS for 1 h. Amersham ECL Western Blotting Detection Reagent (GE Healthcare Japan, Tokyo, Japan) and ImmunoStar^®^ Zeta (Fujifilm Wako Pure Chemical Corporation) were used to detect protein bands by a chemiluminescence reaction. Protein bands were detected by an Amersham Imager 680 (GE Healthcare Japan) and analyzed using ImageQuant TL software 7.0.1.0 (GE Healthcare Japan). Stripping Solution (Fujifilm Wako Pure Chemical Corporation) was used to treat the nitrocellulose membranes before reprobing.

### 4.7. Transfection

A549 cells were transfected with the expression vectors using the HilyMax transfection reagent (Dojindo Laboratories, Kumamoto, Japan) for 24 h.

### 4.8. Statistical Analysis

Means and standard errors were calculated from three independent experiments. Multiple comparisons were performed using one-way ANOVA followed by Tukey’s post hoc test. The creation of graphs and statistical analyses were performed using KaleidaGraph 4.5 software (Hulinks, Tokyo, Japan).

## 5. Conclusions

We herein demonstrated for the first time that sesquiterpenes possessing an α-methylene-γ-lactone moiety promoted the ectodomain shedding of TNF-R1 via TACE activation. The presence of other functional groups, such as an α,β-unsaturated carbonyl moiety, may enhance the ability of eudesmane-type sesquiterpene lactones to induce the proteolytic cleavage of TNF-R1 ectodomains. To date, sesquiterpene lactones have been shown to target several proteins, including IκB kinase β and RelA, by directly binding to cysteine residues, resulting in the inhibition of the NF-κB signaling pathway. The present results showed that an α-methylene-γ-lactone moiety was essential for sesquiterpene lactones to down-regulate TNF-R1 expression through ectodomain shedding. The TNF-R1 signaling pathway is involved in diverse biological processes, including cell growth, cell death, development, oncogenesis, immunity, inflammation, and stress responses. Therefore, the selective targeting of TNF-R1 by sesquiterpene lactones may provide important insights for the development of a new generation of anti-inflammatory and anticancer drugs.

## Data Availability

The data presented in this study are available upon reasonable request.

## Supplementary Materials

The following supporting information can be downloaded at: https://www.mdpi.com/article/10.3390/molecules29081866/s1, Figure S1: Original blots in Figure 1B; Figure S2: Original blots (1) in Figure 1C; Figure S3: Original blots (2) in Figure 1C; Figure S4: Original blots (3) in Figure 1C; Figure S5: Original blots in Figure 2A; Figure S6: Original blots (1) in Figure 2B; Figure S7: Original blots (2) in Figure 2B; Figure S8: Original blots (3) in Figure 2B; Figure S9: Original blots in Figure 2C; Figure S10: Original blots (1) in Figure 2D; Figure S11: Original blots (2) in Figure 2D; Figure S12: Original blots (3) in Figure 2D; Figure S13: Original blots in Figure 2E; Figure S14: Original blots (1) in Figure 2F; Figure S15: Original blots (2) in Figure 2F; Figure S16: Original blots (3) in Figure 2F; Figure S17: Original blots in Figure 3A; Figure S18: Original blots (1) in Figure 3B; Figure S19: Original blots (2) in Figure 3B; Figure S20: Original blots (3) in Figure 3B; Figure S21: Original blots in Figure 4A; Figure S22: Original blots (1) in Figure 4B; Figure S23: Original blots (2) in Figure 4B; Figure S24: Original blots (3) in Figure 4B; Figure S25: Original blots in Figure 5A; Figure S26: Original blots (1) in Figure 5B,C; Figure S27: Original blots (2) in Figure 5B,C; Figure S28: Original blots (3) in Figure 5B,C; Figure S29: Original blots in Figure 6A; Figure S30: Original blots (1) in Figure 6B; Figure S31: Original blots (2) in Figure 6B; Figure S32: Original blots (3) in Figure 6B; Figure S33: Original blots in Figure 8A; Figure S34: Original blots (1) in Figure 8B,C; Figure S35: Original blots (2) in Figure 8B,C; Figure S36: Original blots (3) in Figure 8B,C; Figure S37: Original blots in Figure 8D; Figure S38: Original blots (1) in Figure 8E,F; Figure S39: Original blots (2) in Figure 8E,F; Figure S40: Original blots (3) in Figure 8E,F; Figure S41: Original blots in Figure 9B; Figure S42: Original blots (1) in Figure 9C,D; Figure S43: Original blots (2) in Figure 9C,D; Figure S44: Original blots (3) in Figure 9C,D; Figure S45: Original blots in Figure 9E; Figure S46: Original blots (1) in Figure 9F,G; Figure S47: Original blots (2) in Figure 9F,G; Figure S48: Original blots (3) in Figure 9F,G.
