# Supplementary material for: Sesquiterpene Lactones Containing an α-Methylene-γ-Lactone Moiety Selectively Down-Regulate the Expression of Tumor Necrosis Factor Receptor 1 by Promoting Its Ectodomain Shedding in Human Lung Adenocarcinoma A549 Cells
Source: Molecules. 2024 Apr 19;29(8):1866. doi: 10.3390/molecules29081866 (PMC11053566; doi:10.3390/molecules29081866)

Figure S1: Original blots in Figure 1B

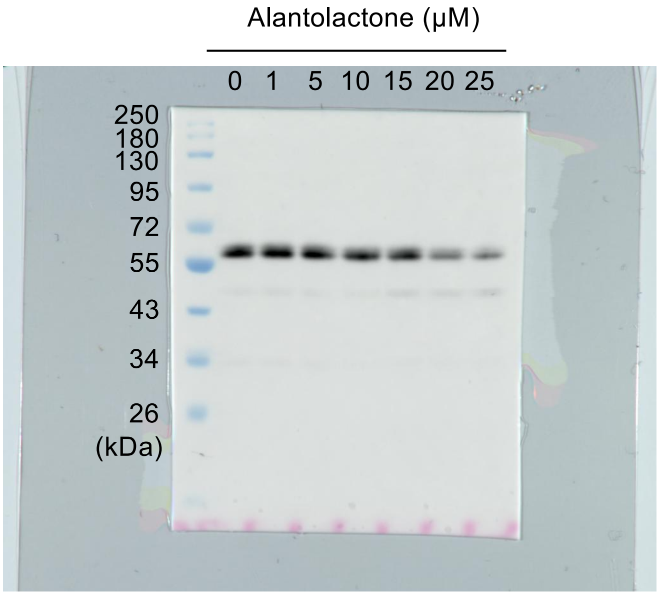

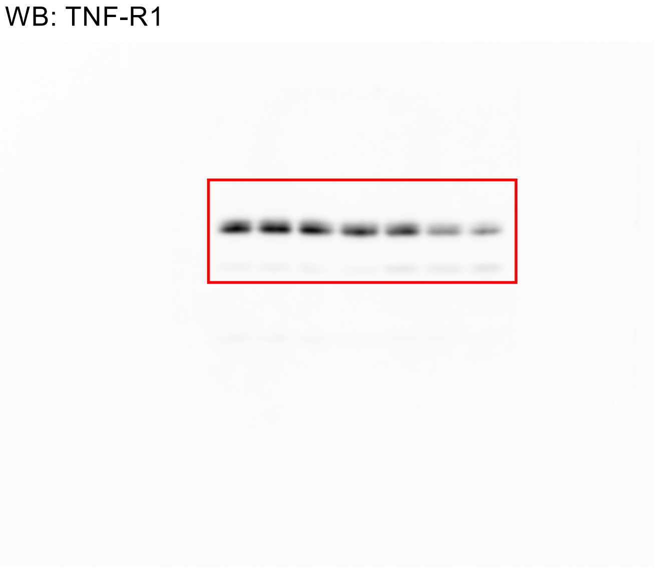

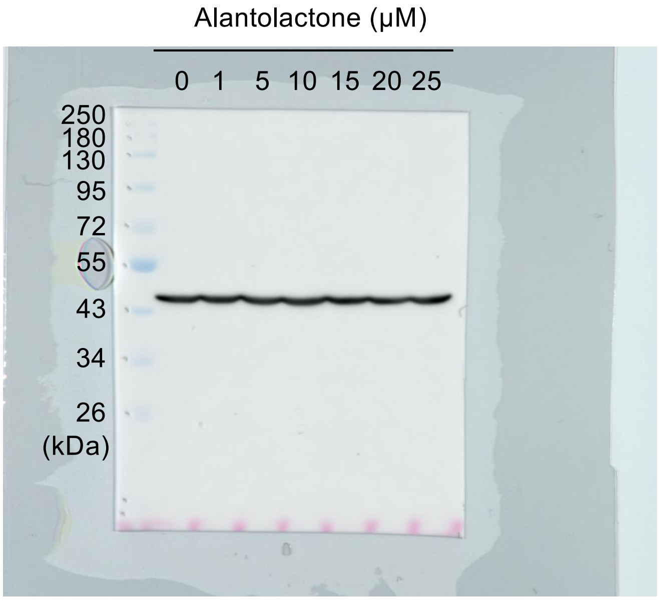

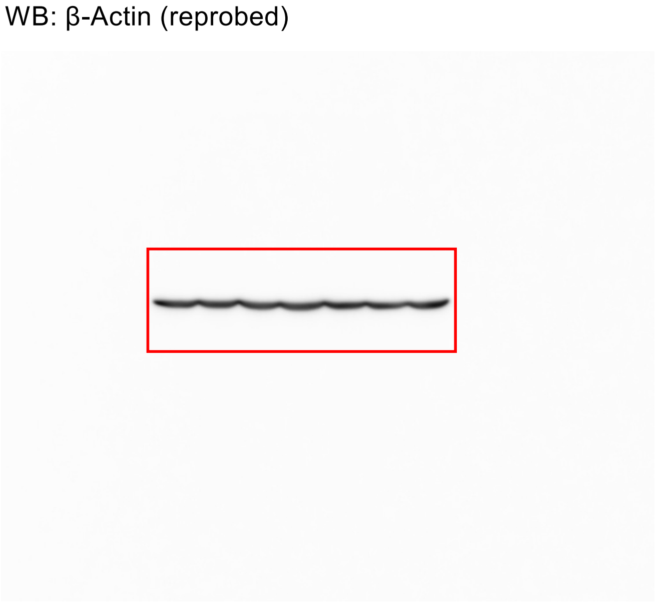

Figure S2: Original blots (1) in Figure 1C

Alantolactone (μM)

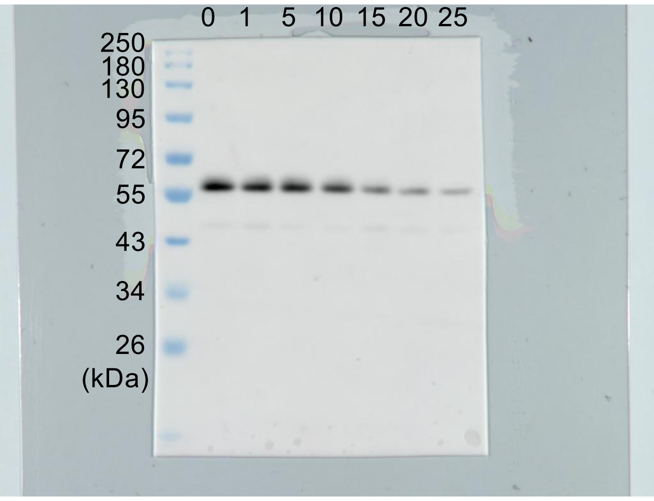

WB: TNF-R1

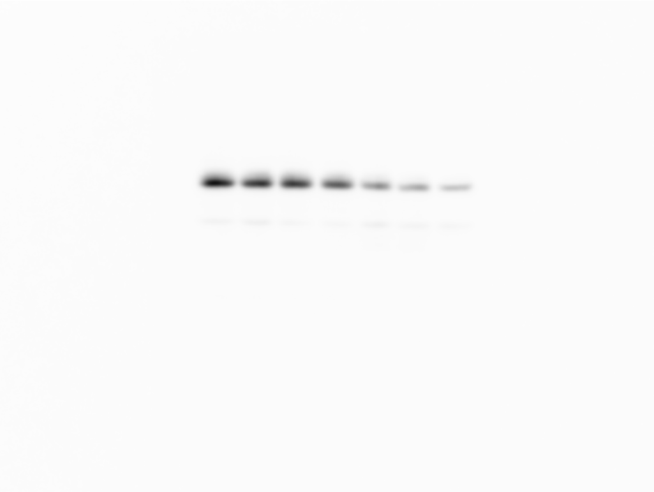

Alantolactone (μM)

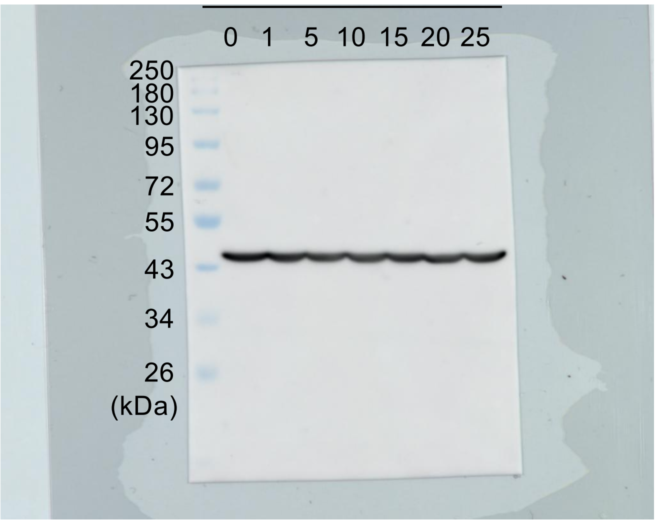

WB: β-Actin (reprobed)

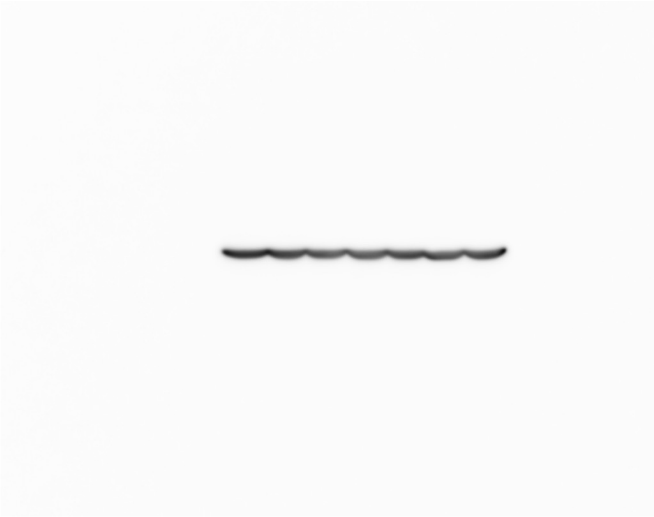

Figure S3: Original blots (2) in Figure 1C

Alantolactone (μM)

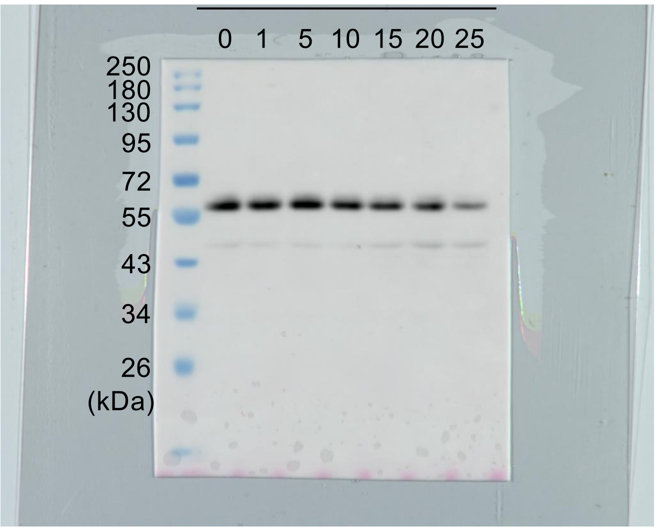

WB: TNF-R1

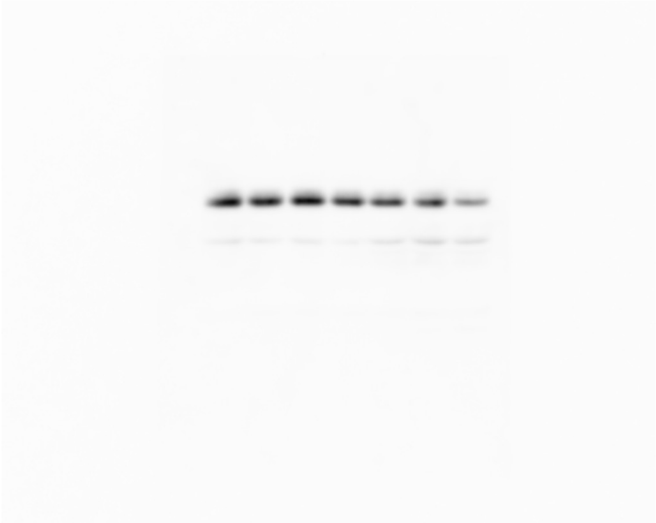

Alantolactone (μM)

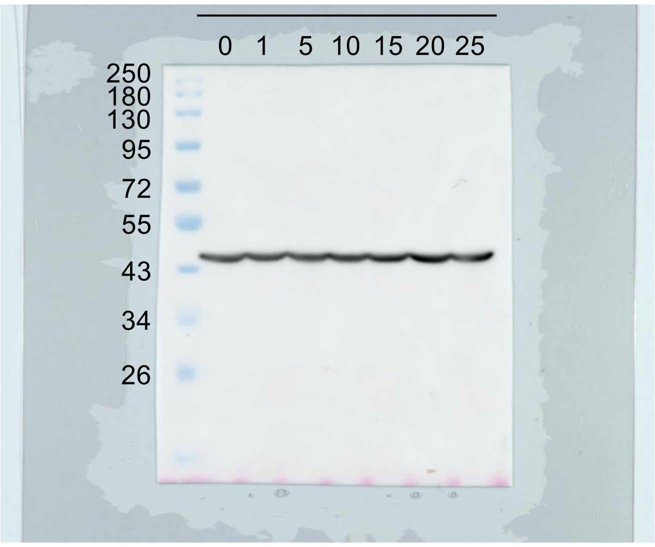

WB: β-Actin (reprobed)

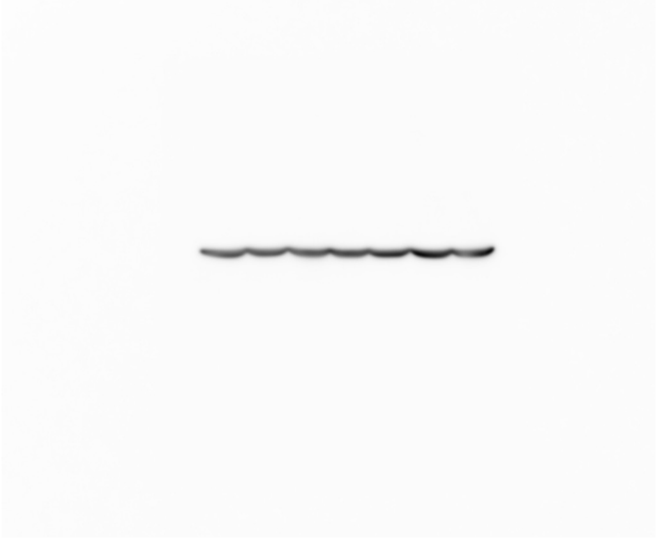

Figure S4: Original blots (3) in Figure 1C

Alantolactone (μM)

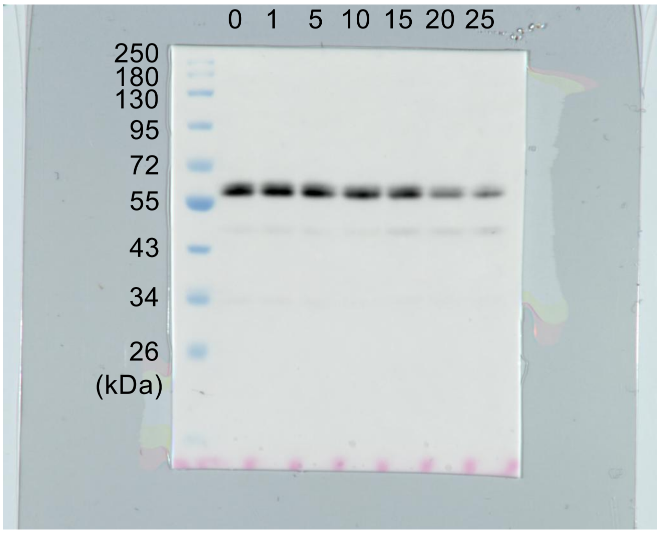

WB: TNF-R1

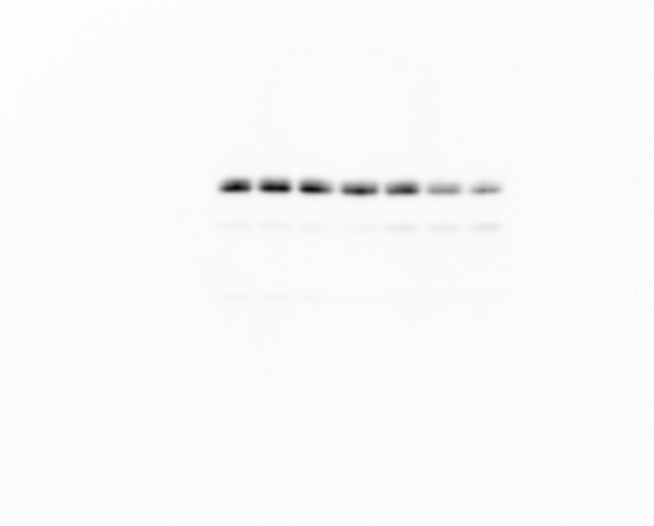

Alantolactone (μM)

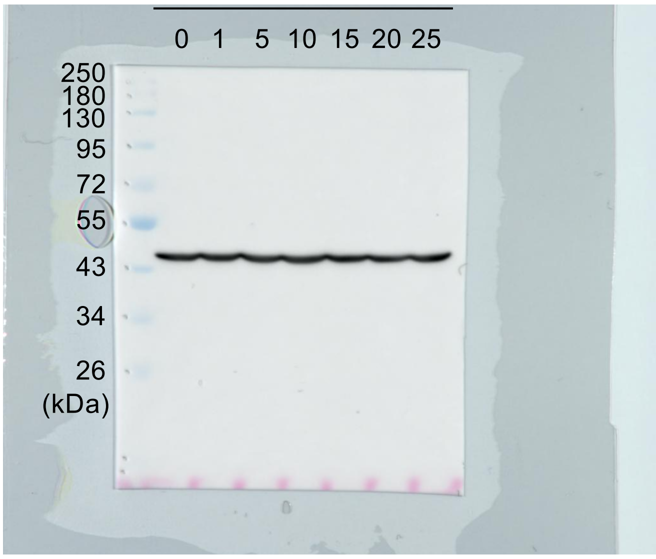

WB: β-Actin (reprobed)

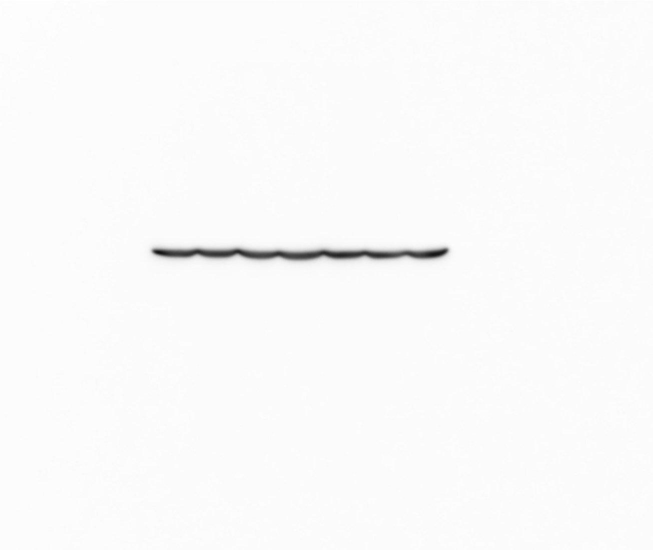

Figure S5: Original blots in Figure 2A

Alantolactone (µM)

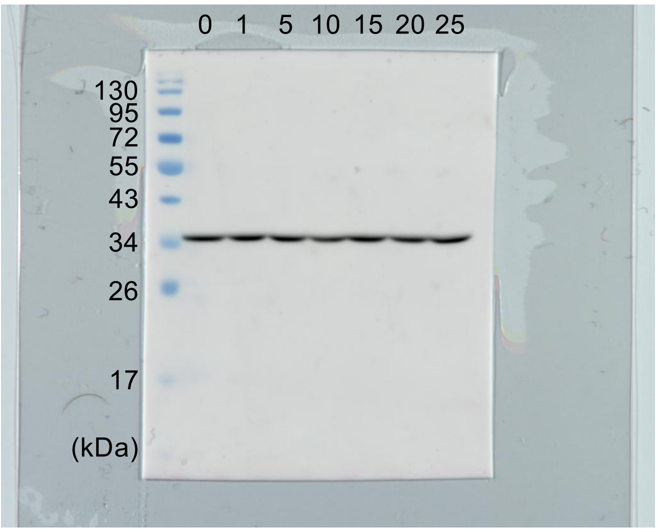

WB: TRADD

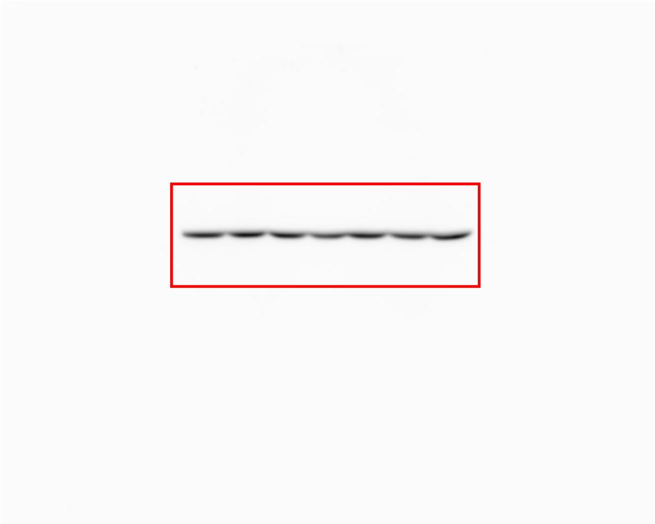

Alantolactone (µM)

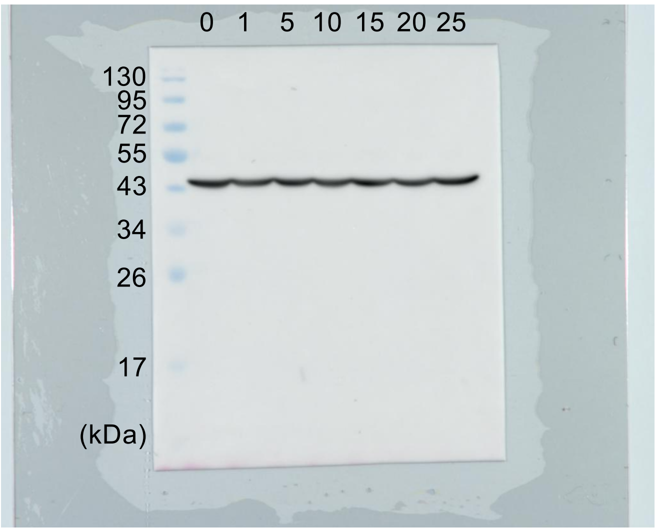

WB: β-Actin (reprobed)

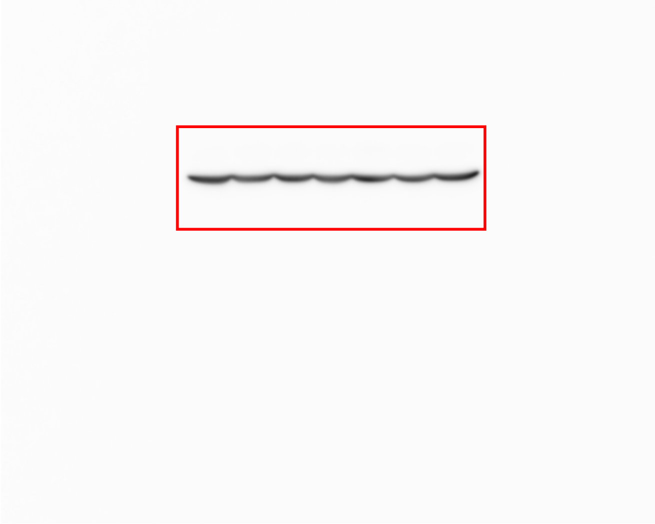

Figure S6: Original blots (1) in Figure 2B

Alantolactone (μM)

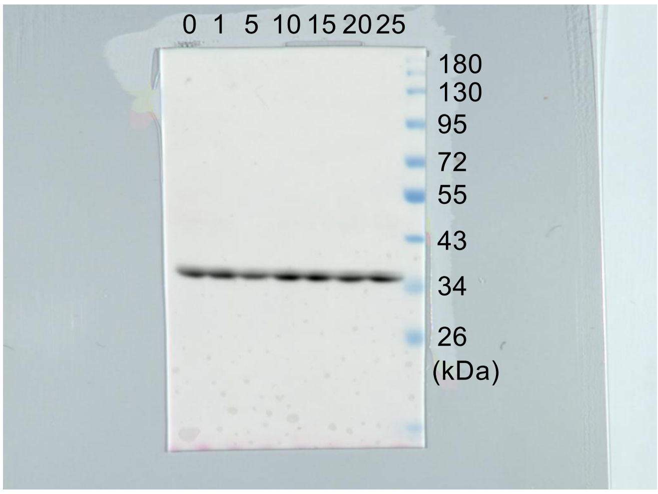

WB: TRADD

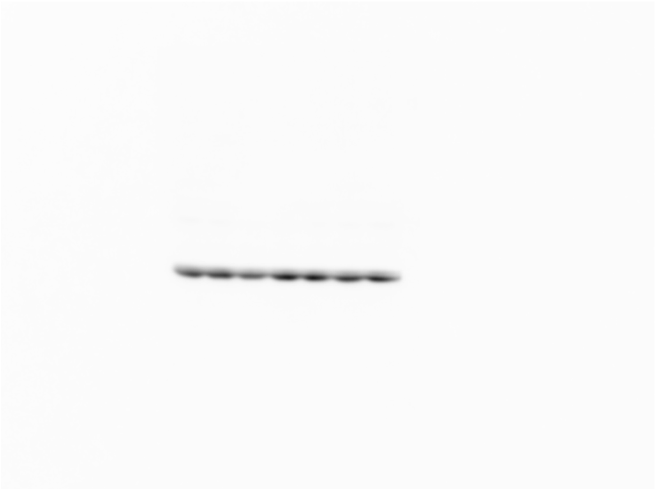

Alantolactone (μM)

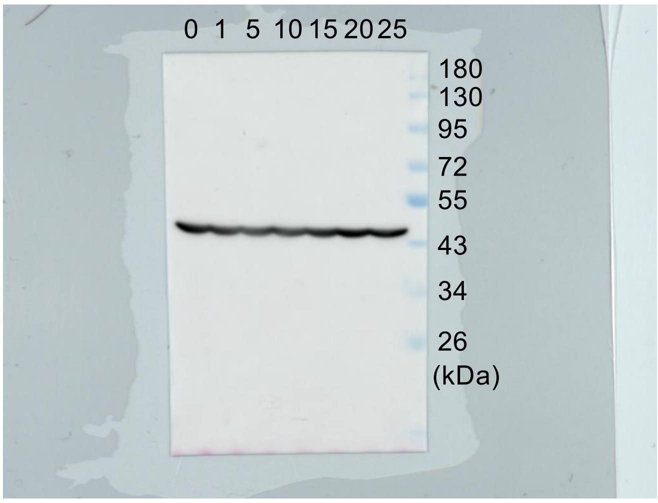

WB: β-Actin (reprobed)

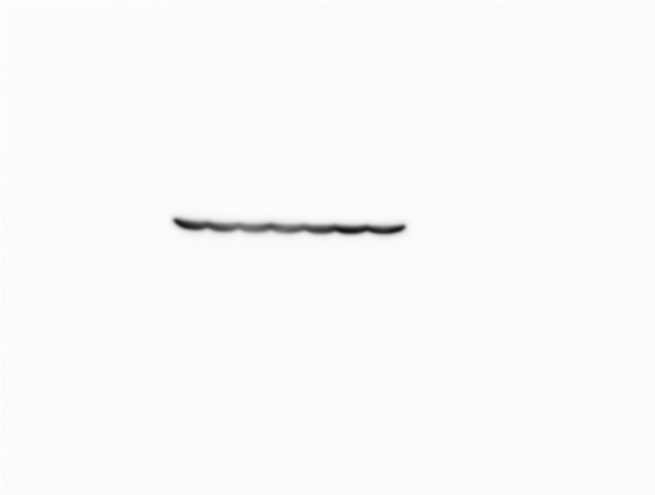

Figure S7: Original blots (2) in Figure 2B

Alantolactone (μM)

WB: TRADD

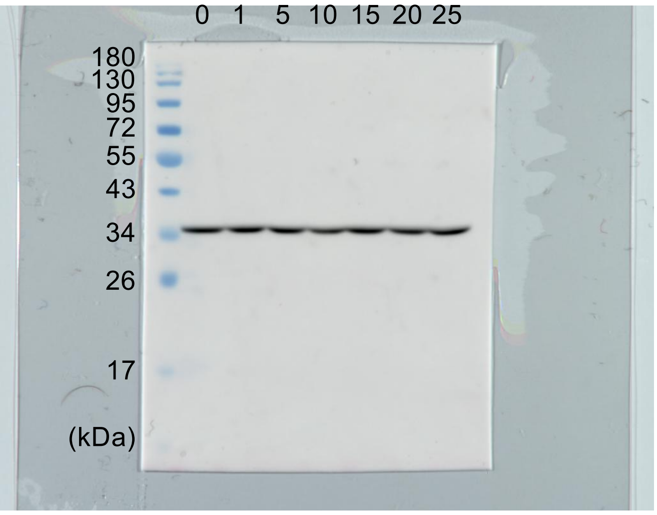

Alantolactone (μM)

WB: β-Actin (reprobed)

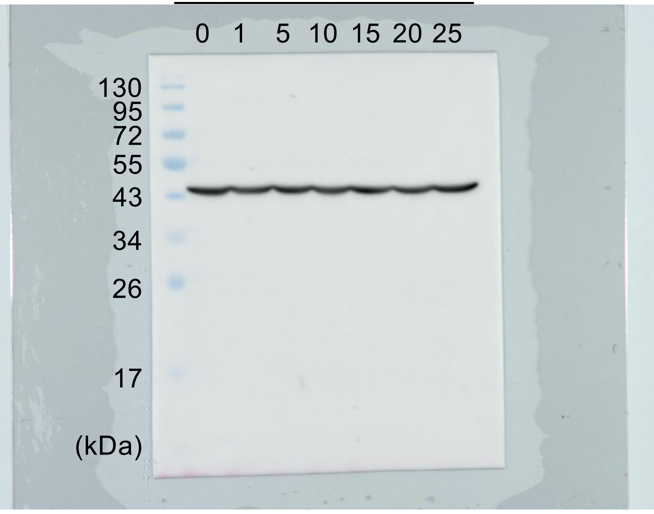

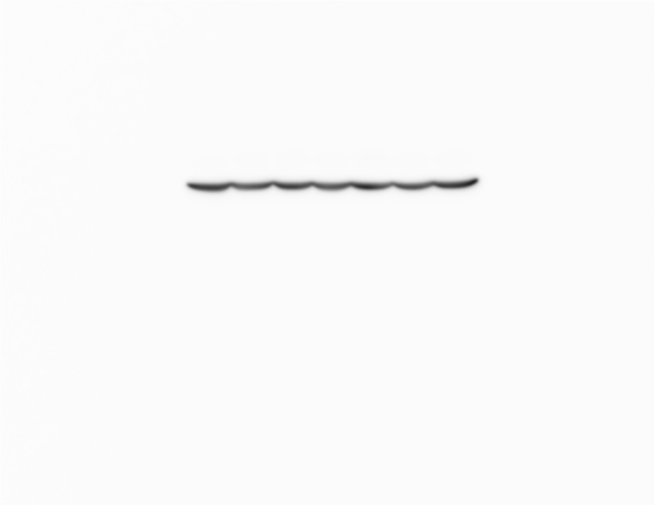

Figure S8: Original blots (3) in Figure 2B

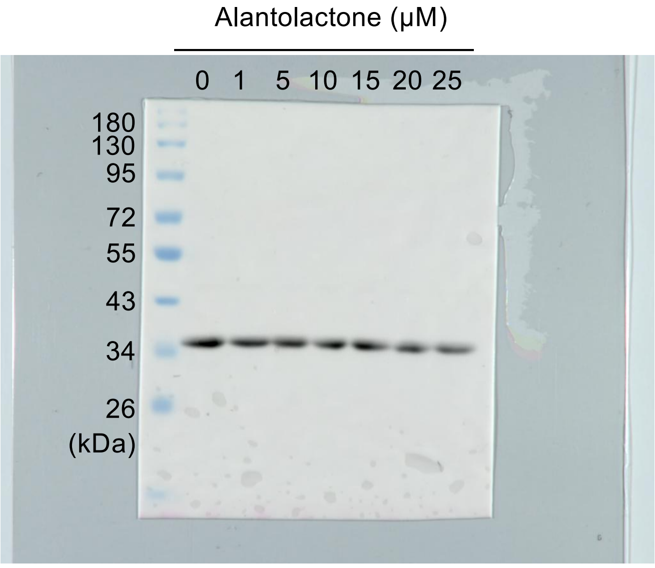

WB: TRADD

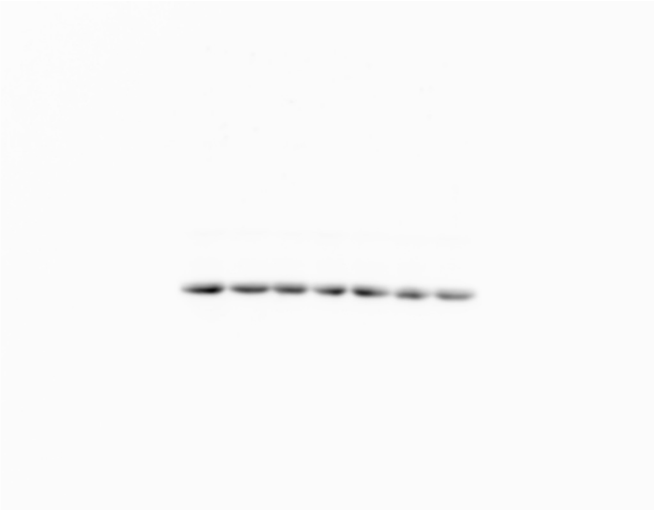

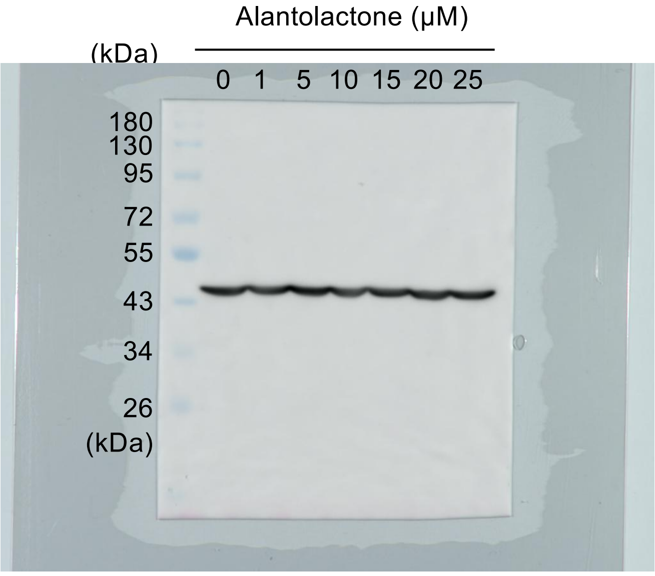

WB: β-Actin (reprobed)

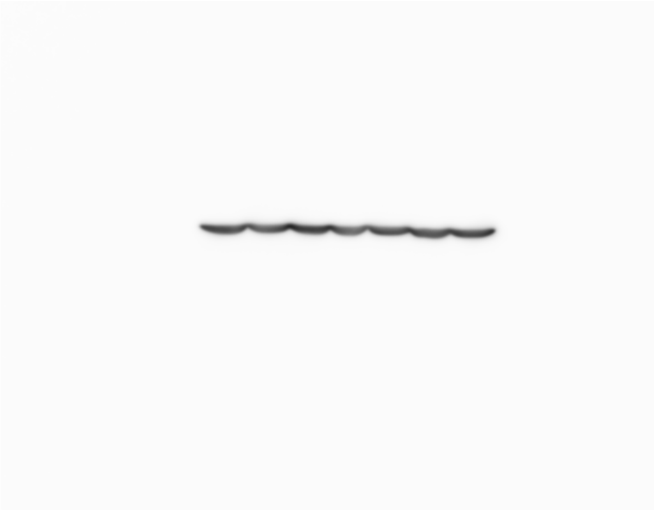

Figure S9: Original blots in Figure 2C

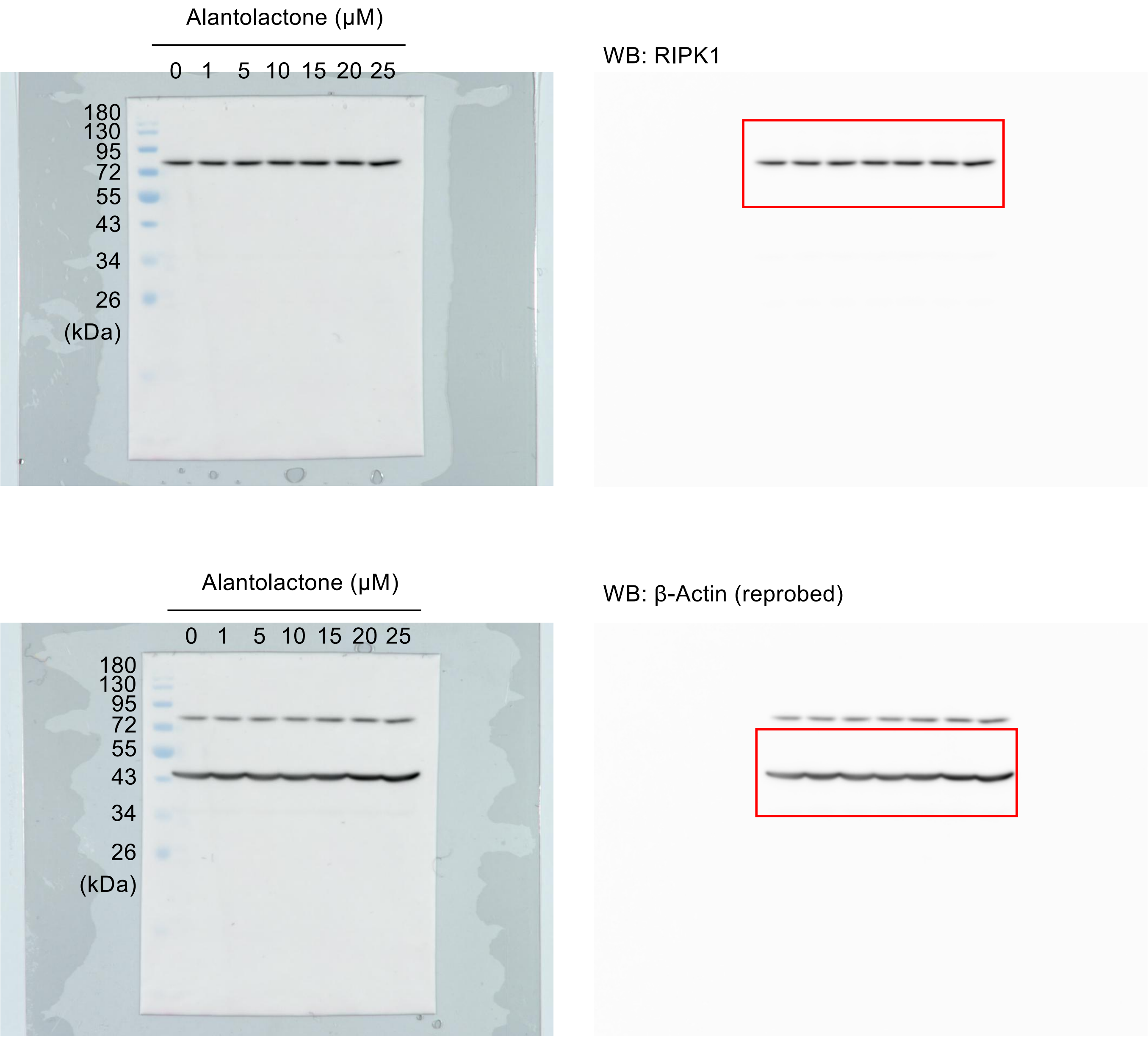

Figure S10: Original blots (1) in Figure 2D

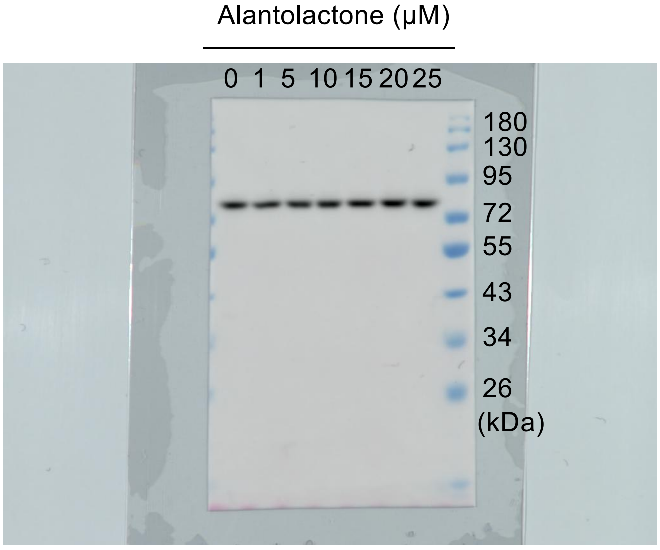

WB: RIPK1

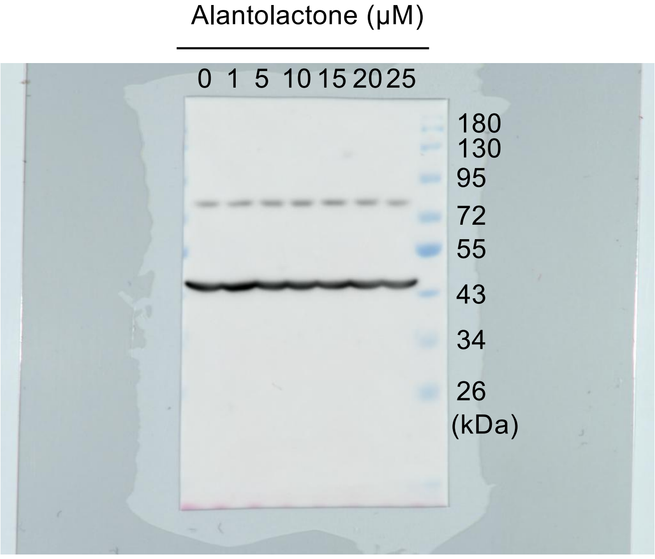

WB: β-Actin (reprobed)

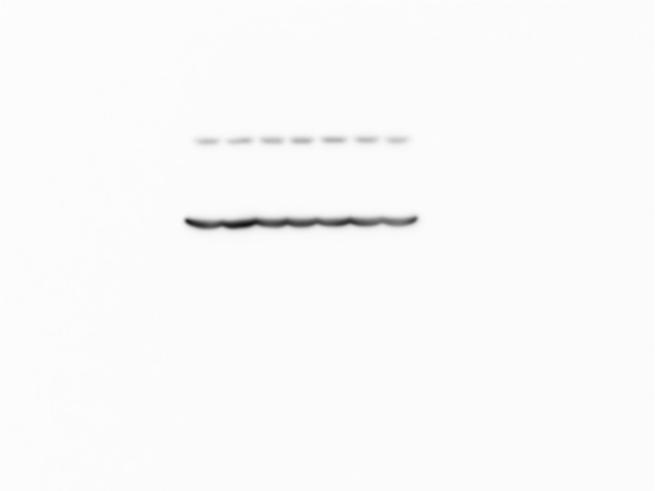

Figure S11: Original blots (2) in Figure 2D

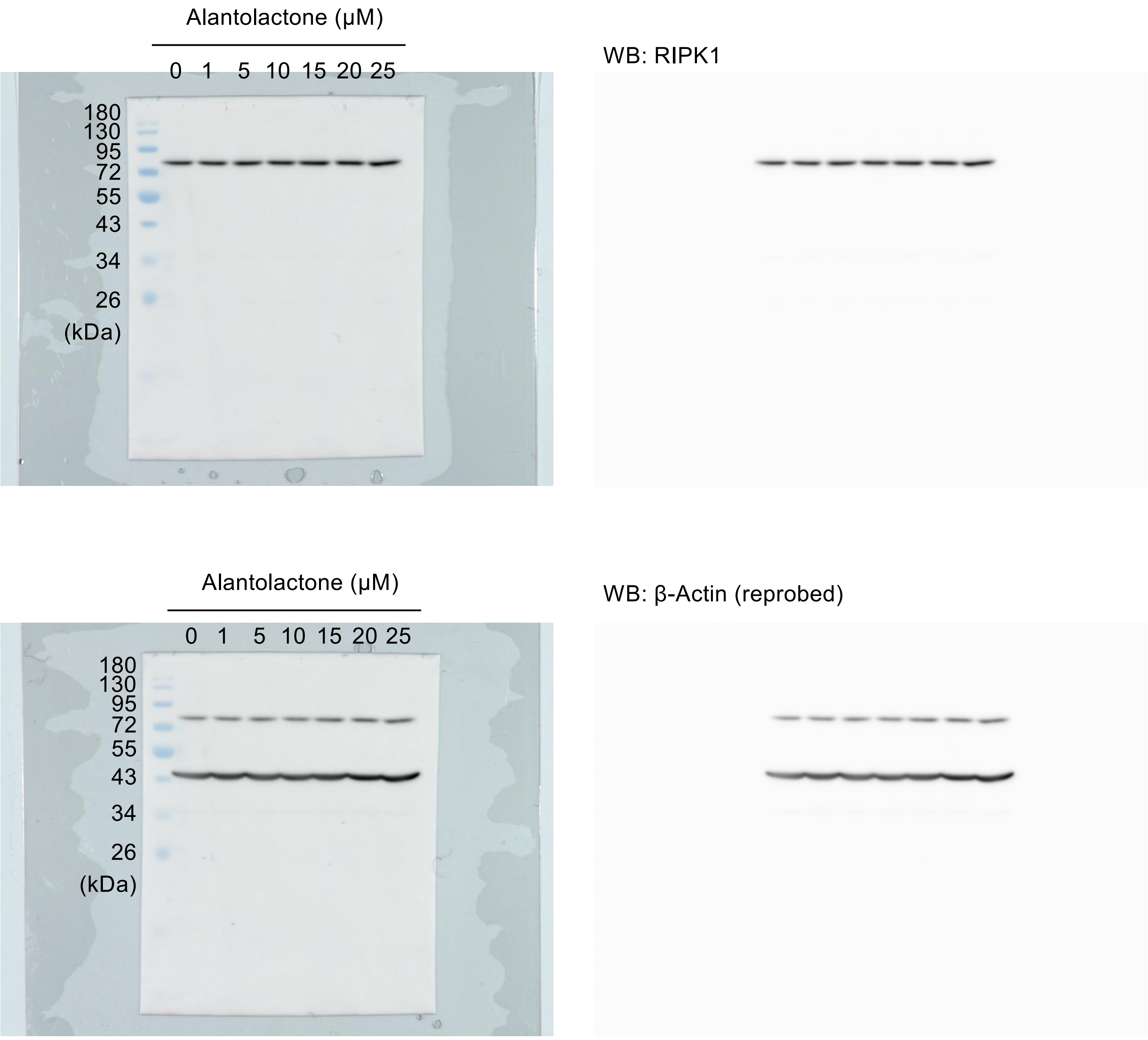

Figure S12: Original blots (3) in Figure 2D

Alantolactone (μM)

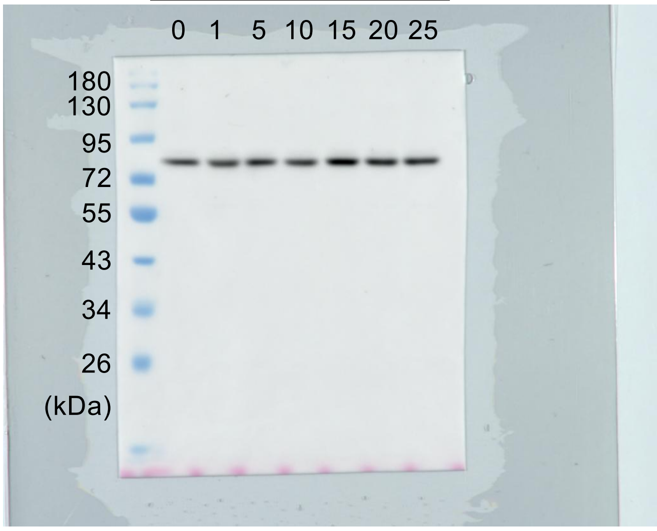

WB: RIPK1

Alantolactone (μM)

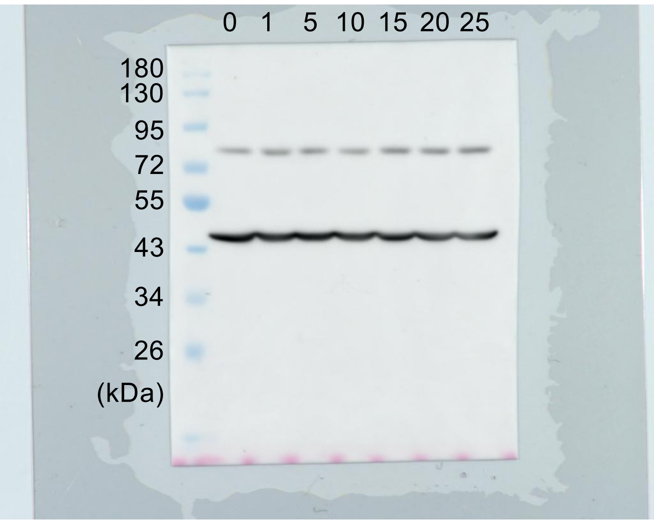

WB: β-Actin (reprobed)

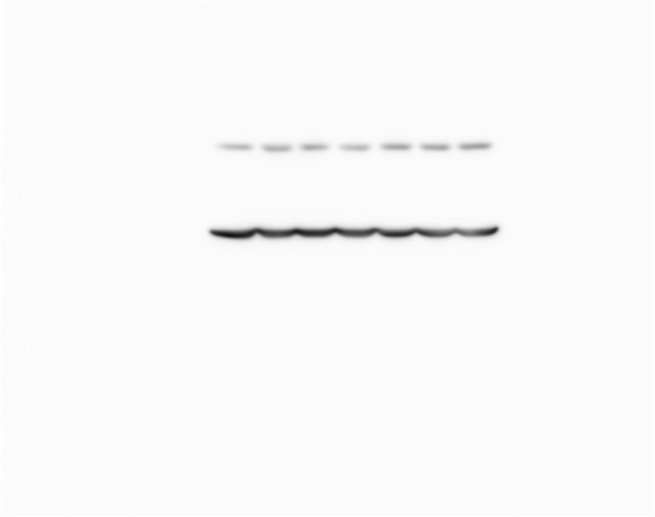

Figure S13: Original blots in Figure 2E

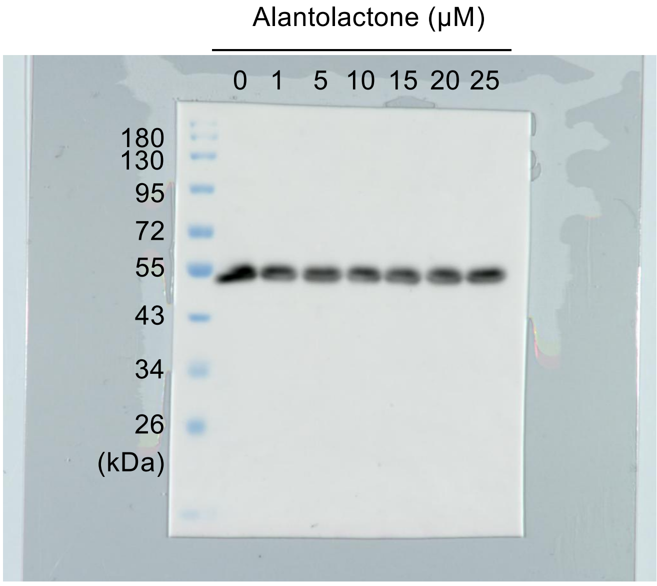

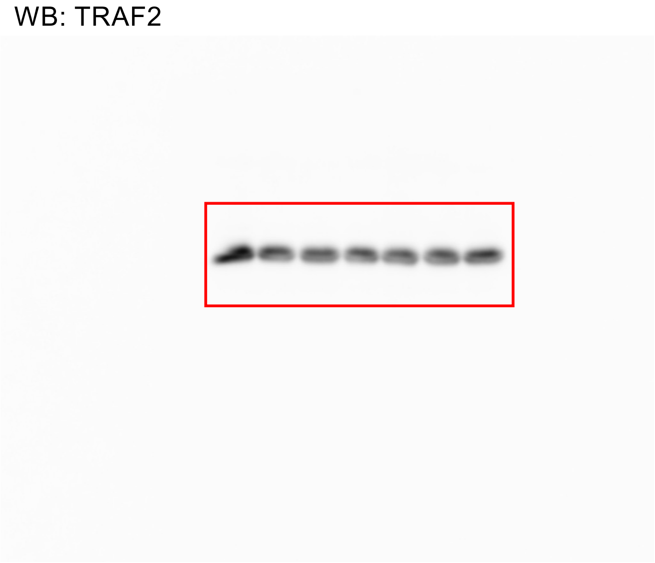

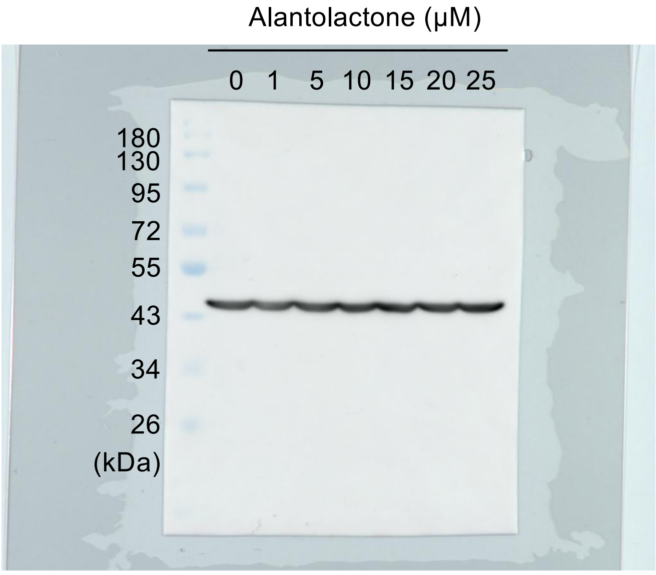

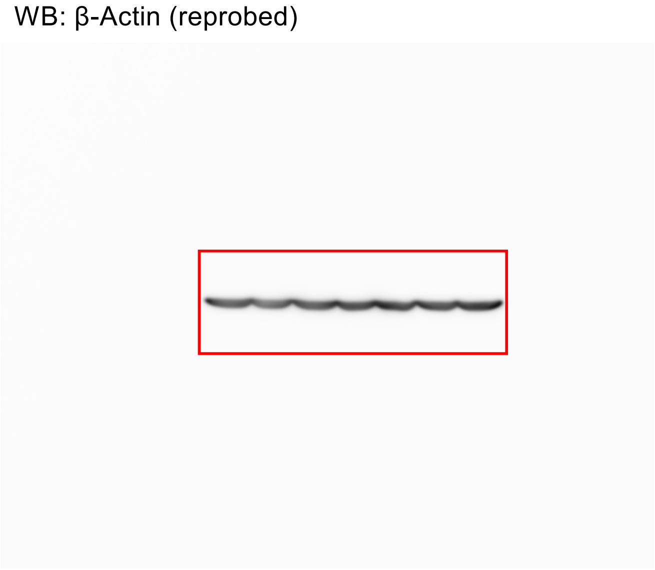

Figure S14: Original blots (1) in Figure 2F

Alantolactone (μM)

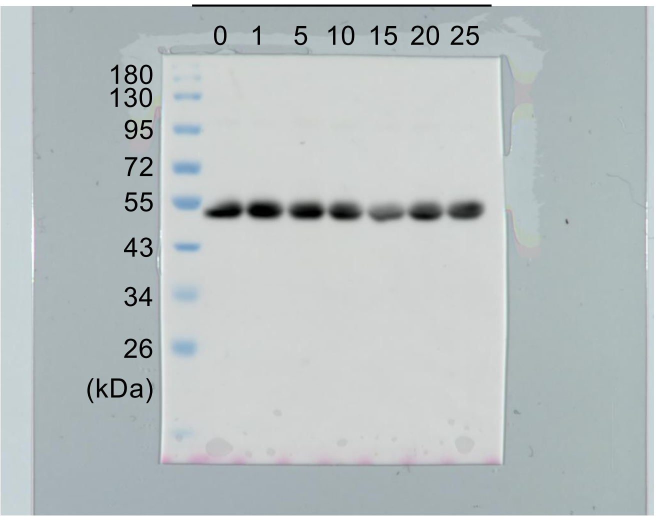

WB: TRAF2

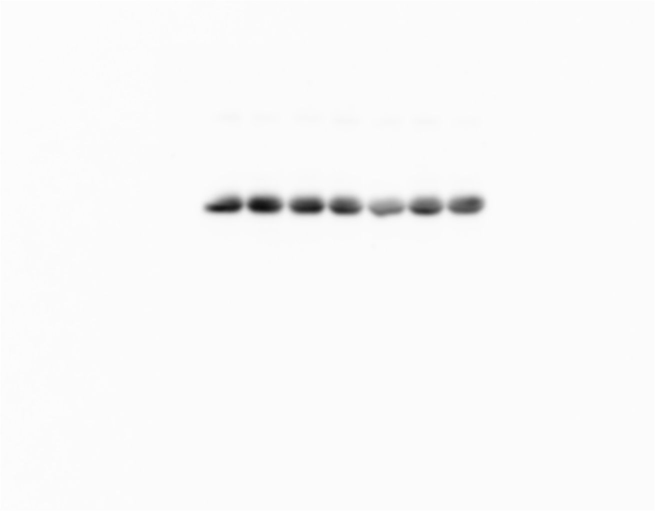

Alantolactone (μM)

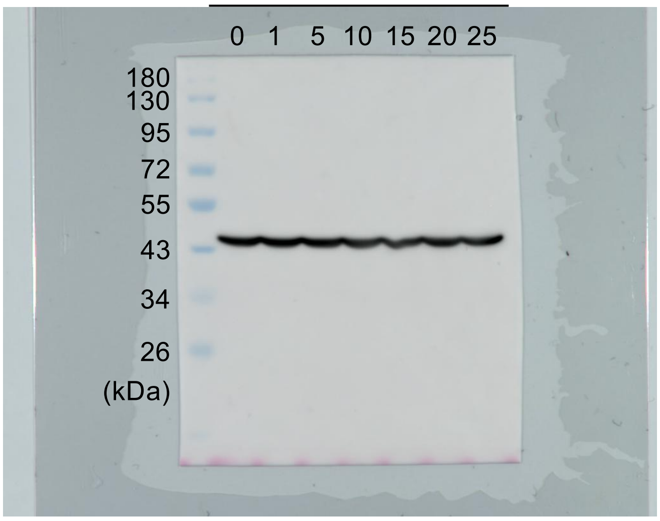

WB: β-Actin (reprobed)

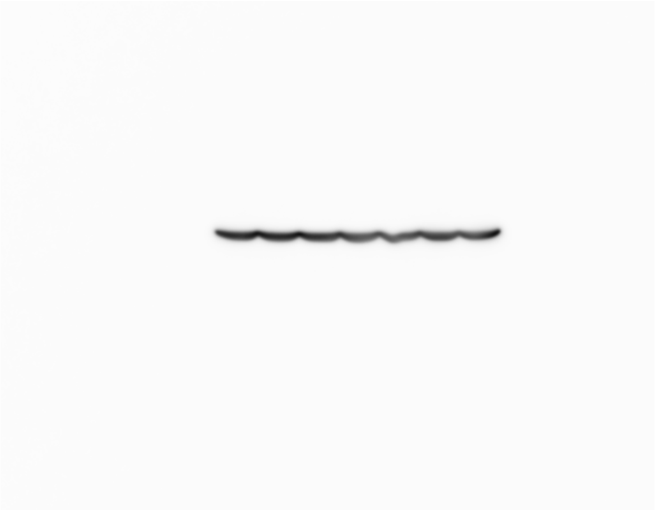

Figure S15: Original blots (2) in Figure 2F

Alantolactone (μM)

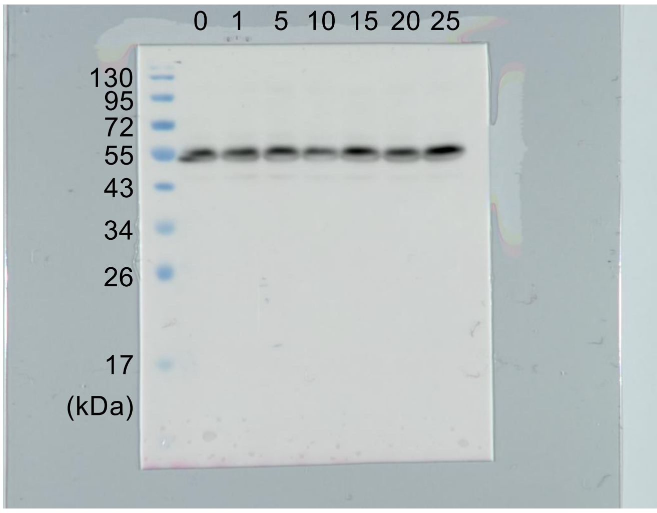

WB: TRAF2

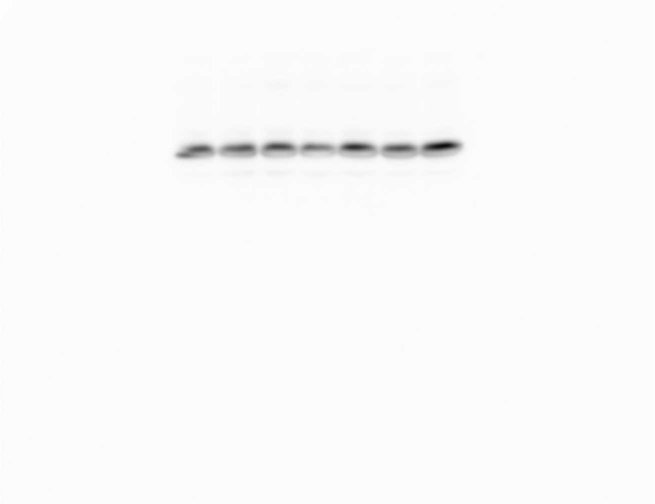

Alantolactone (μM)

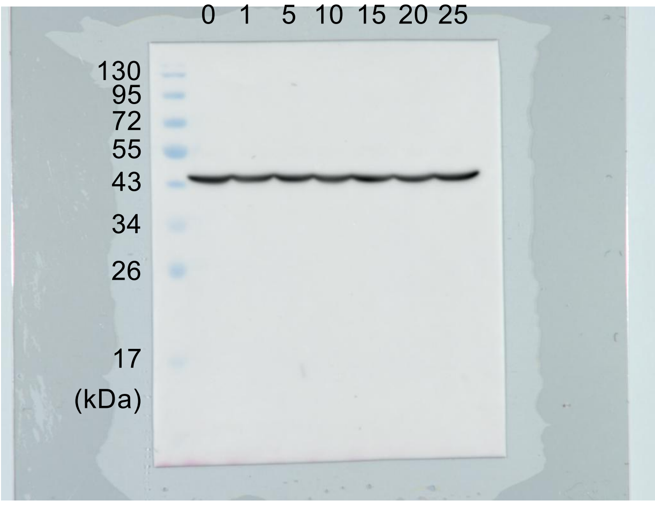

WB: β-Actin (reprobed)

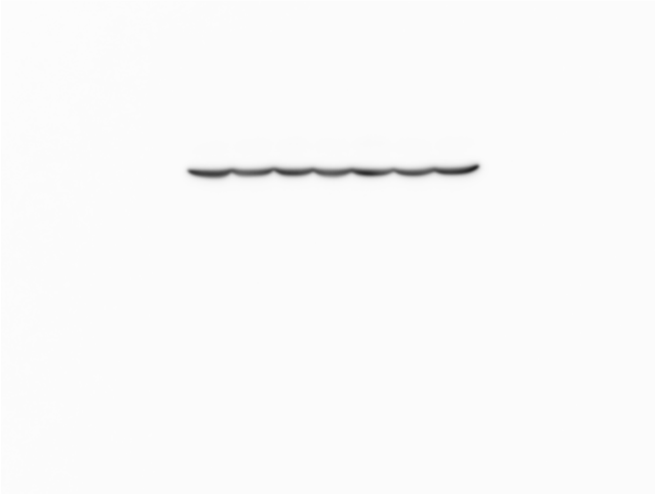

Figure S16: Original blots (3) in Figure 2F

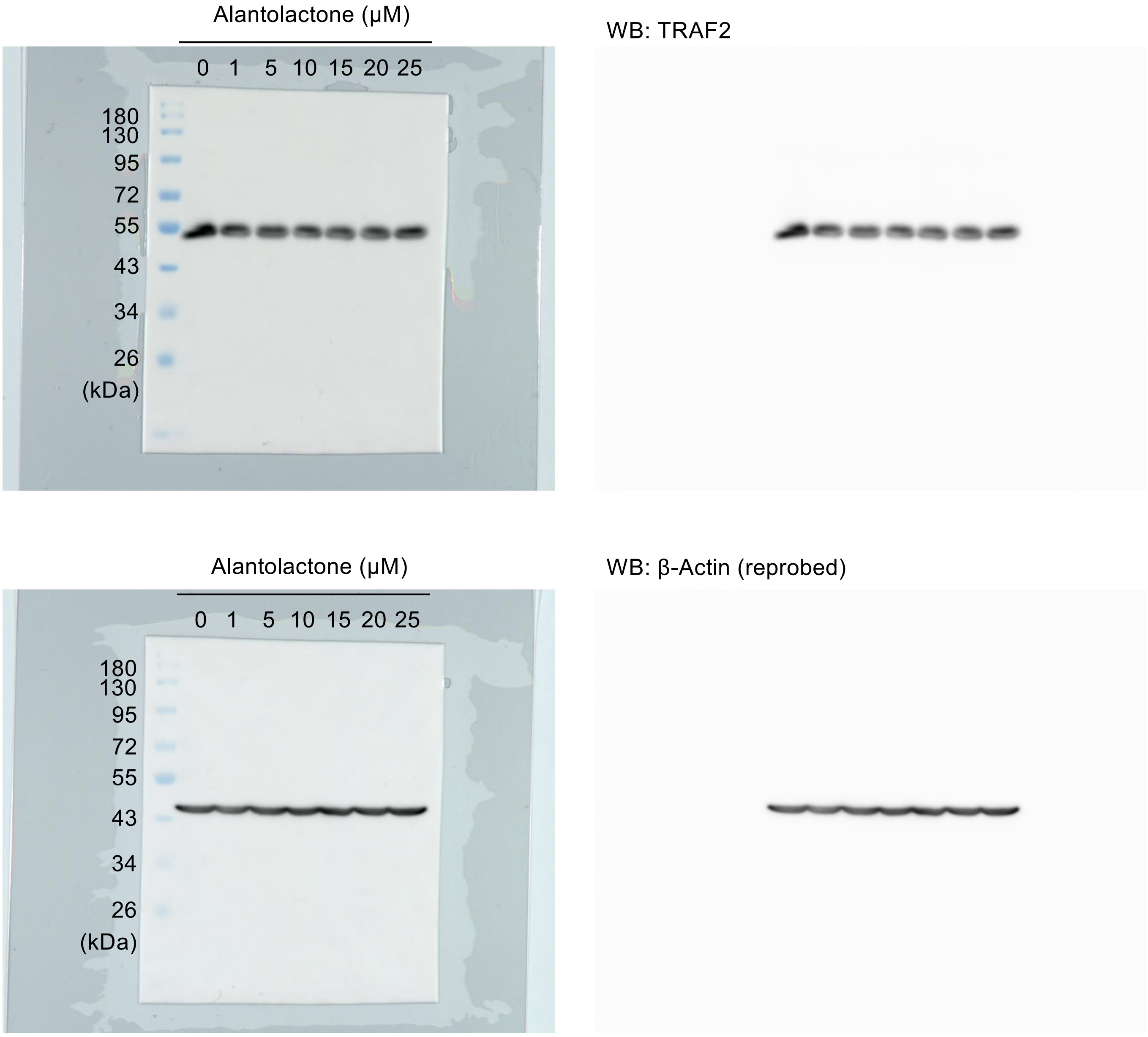

Figure S17: Original blots in Figure 3A

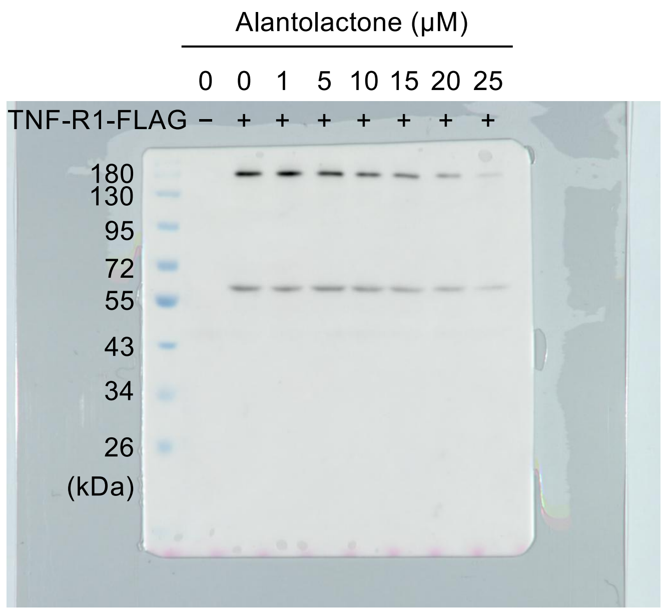

WB: TNF-R1

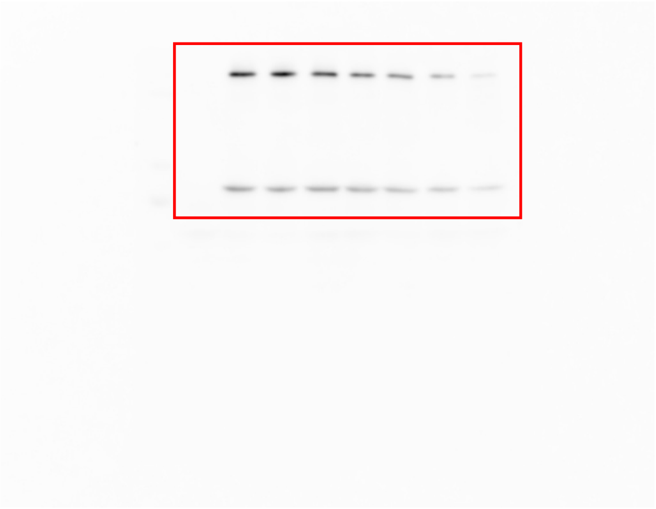

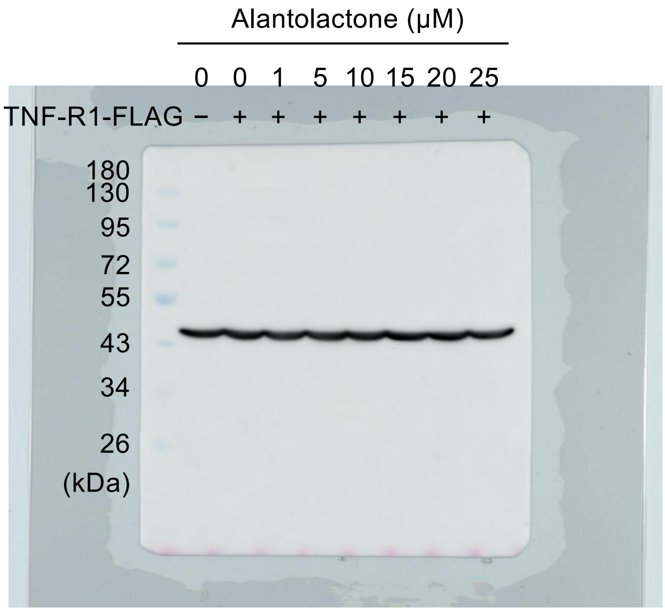

WB: β-Actin (reprobed)

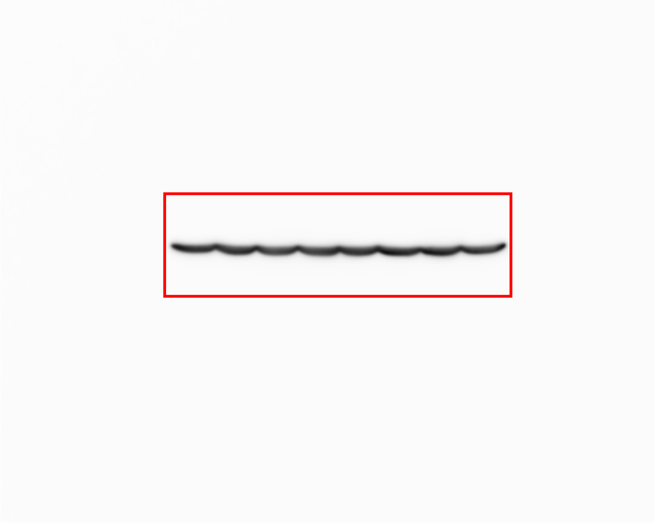

Figure S18: Original blots (1) in Figure 3B

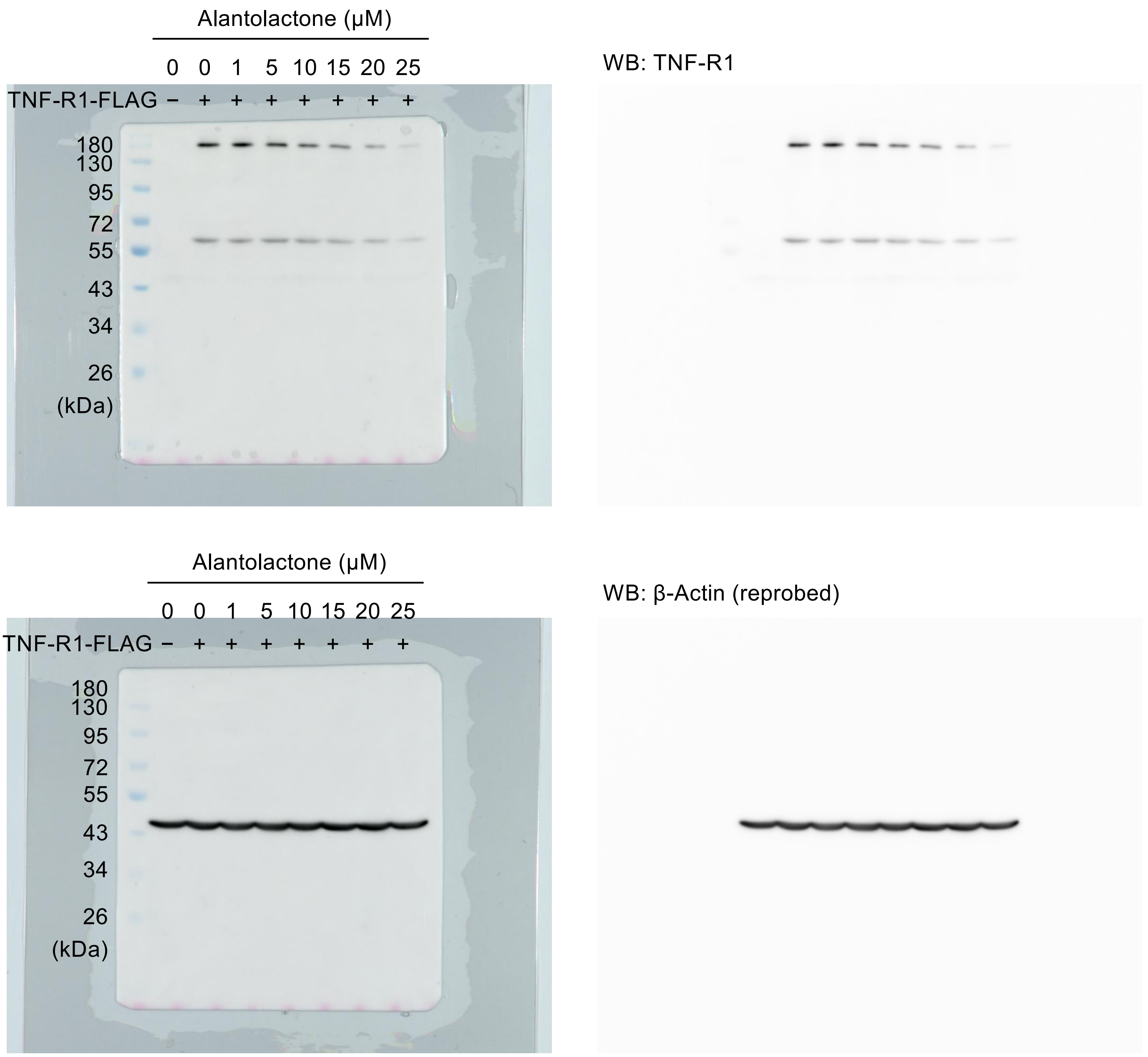

Figure S19: Original blots (2) in Figure 3B

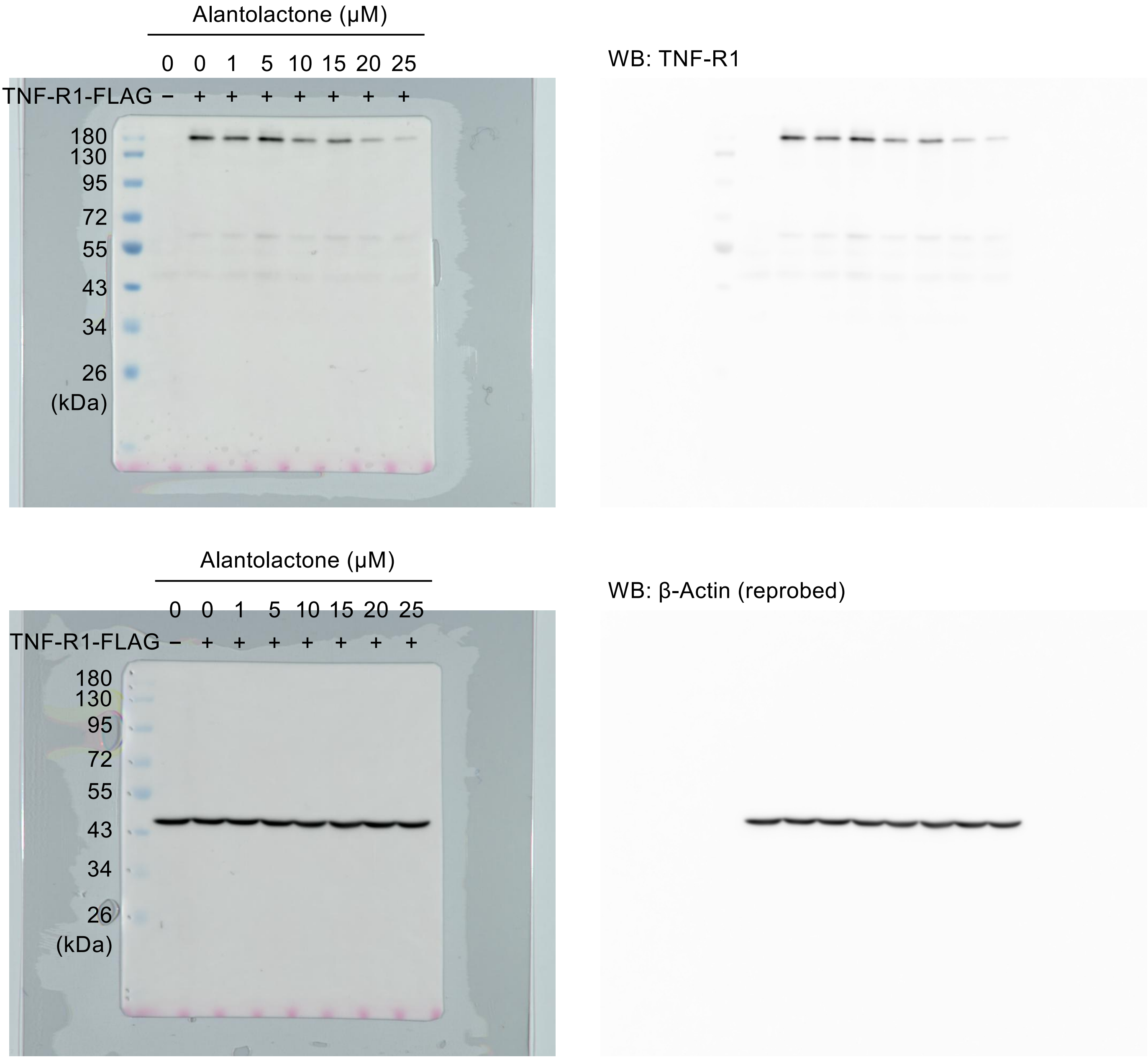

Figure S20: Original blots (3) in Figure 3B

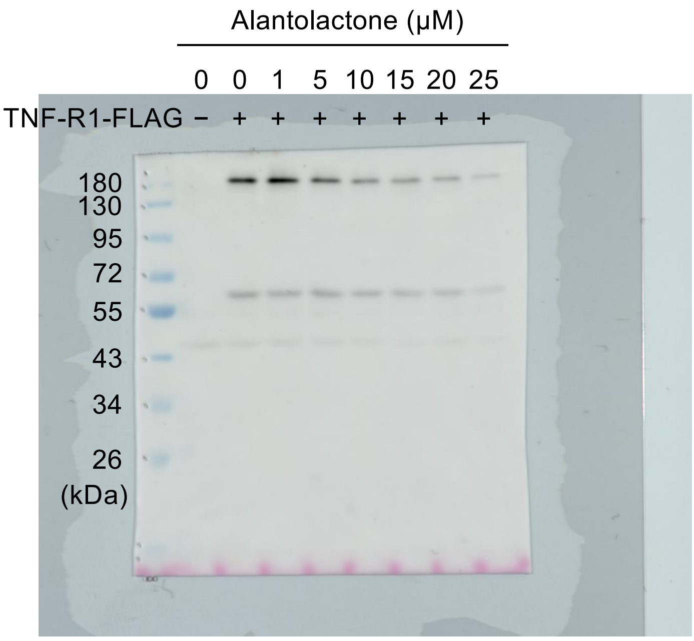

WB: TNF-R1

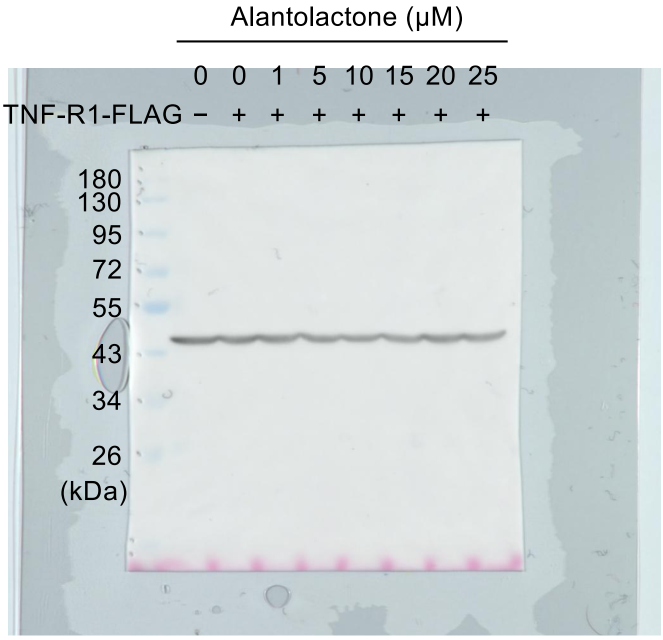

WB: β-Actin (reprobed)

Figure S21: Original blots in Figure 4A

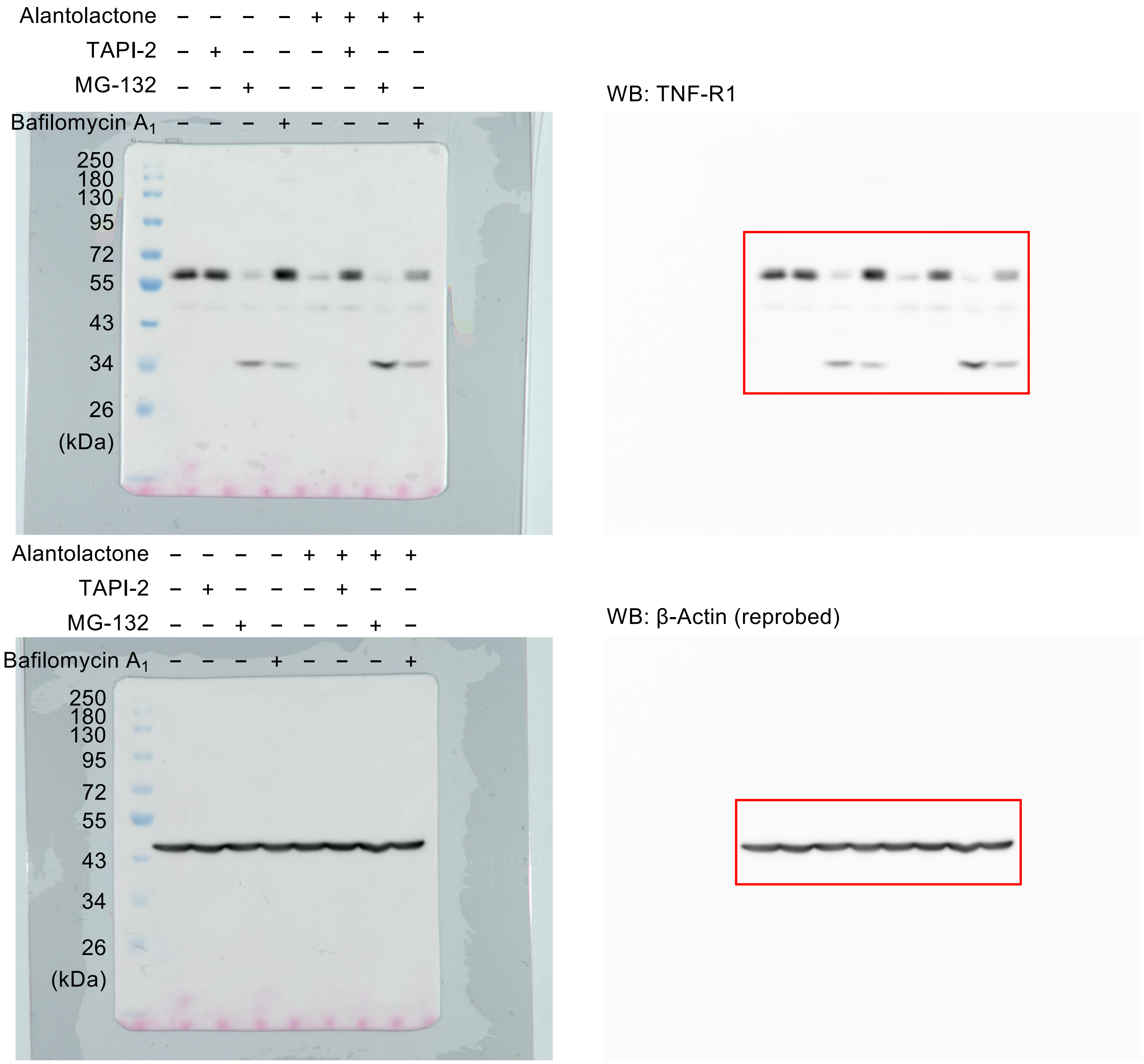

Figure S22: Original blots (1) in Figure 4B

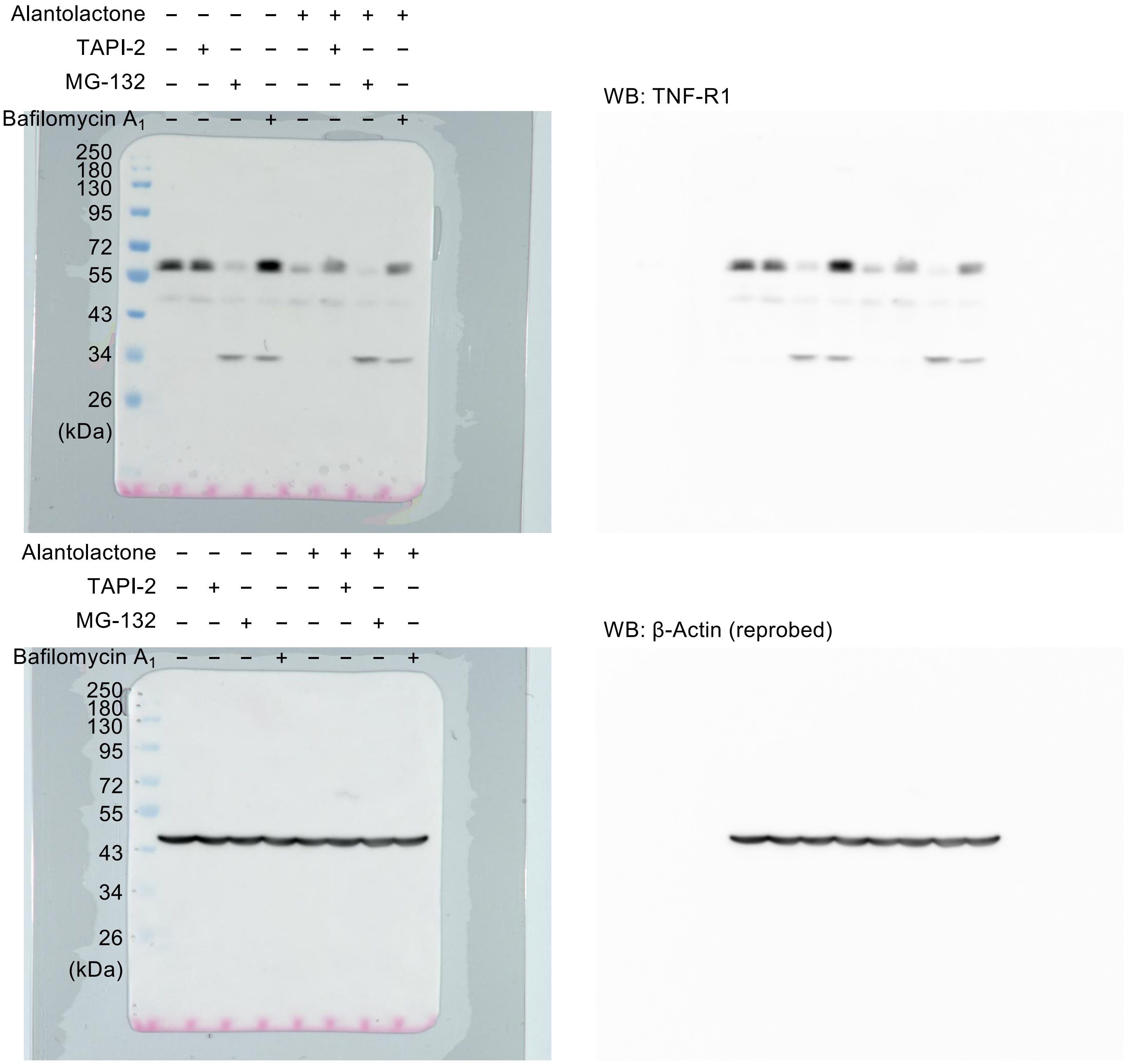

Figure S23: Original blots (2) in Figure 4B

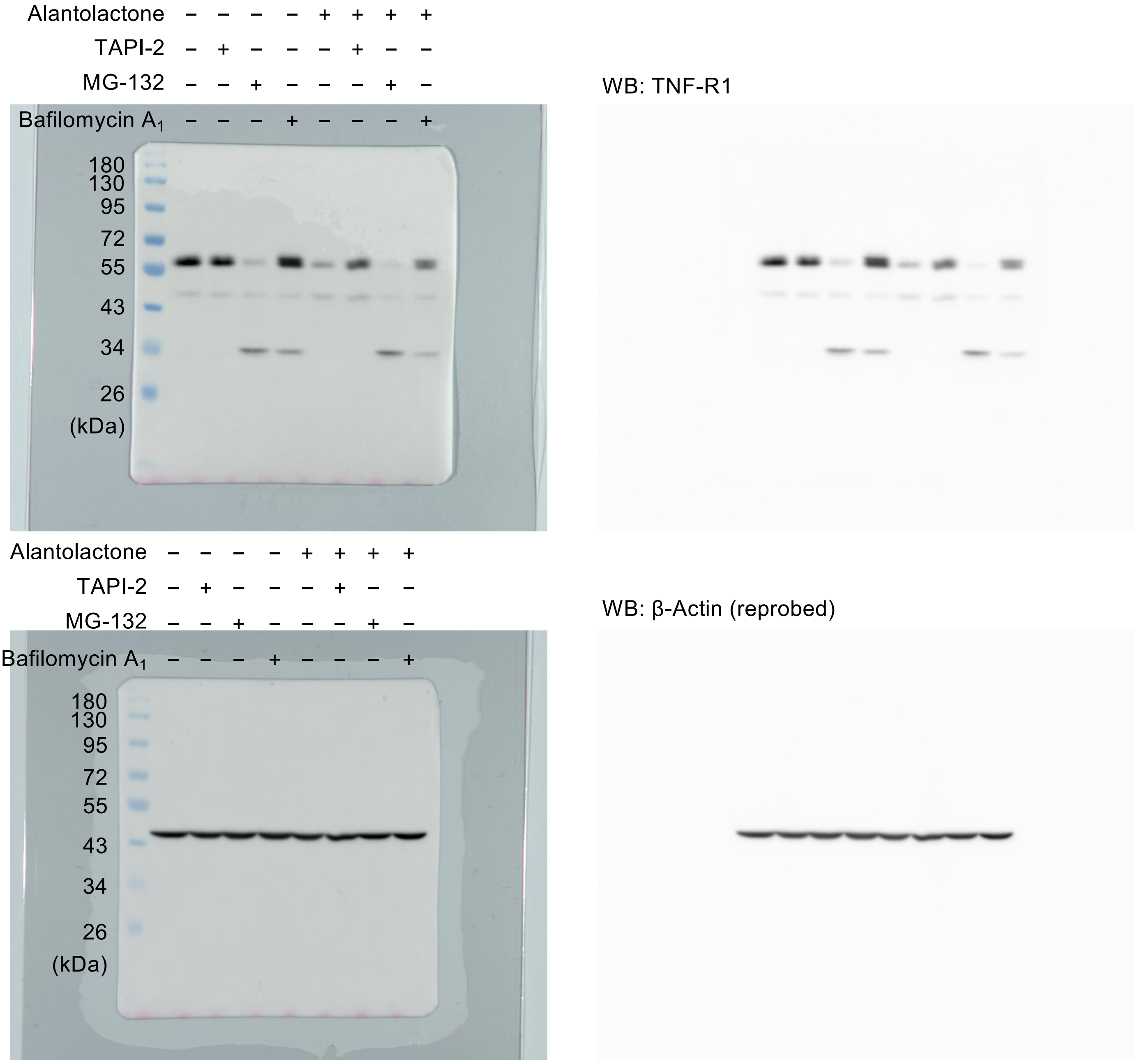

Figure S24: Original blots (3) in Figure 4B

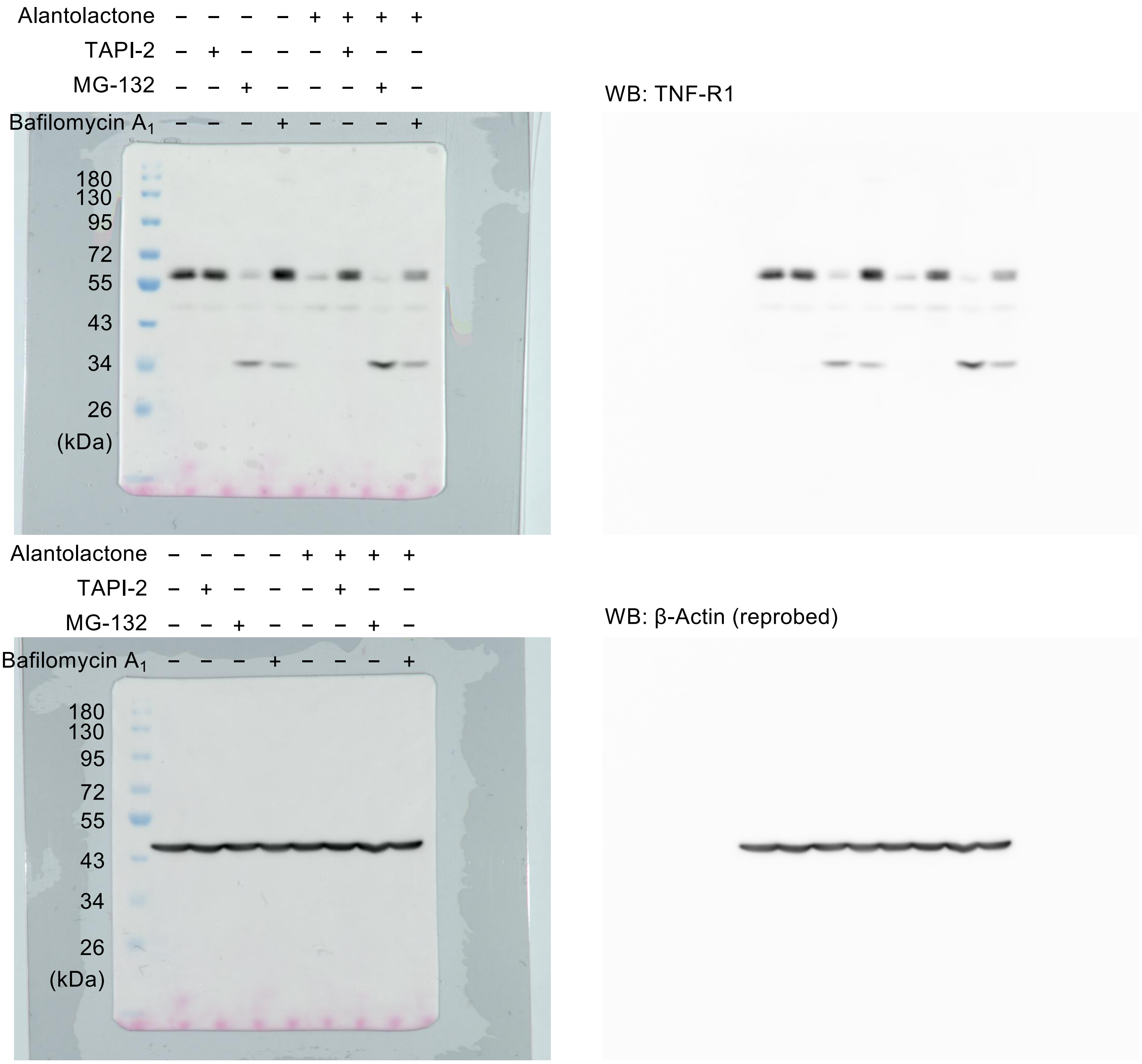

Figure S25: Original blots in Figure 5A

Alantolactone (μM)

0 1 5 10 15 20 25

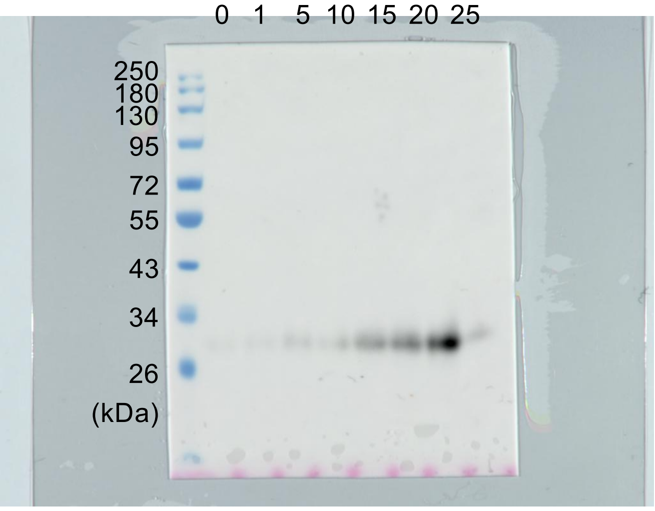

WB: TNF-R1

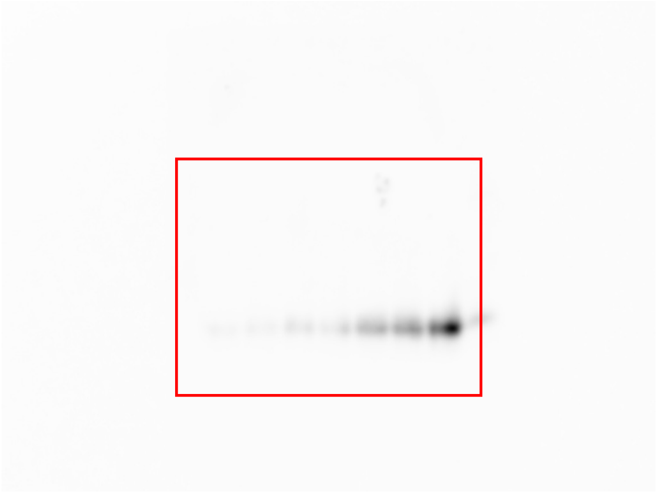

Alantolactone (μM)

0 1 5 10 15 20 25

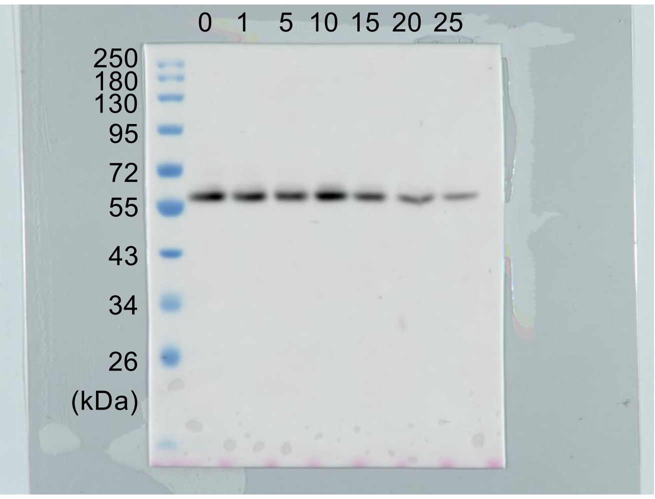

WB: TNF-R1

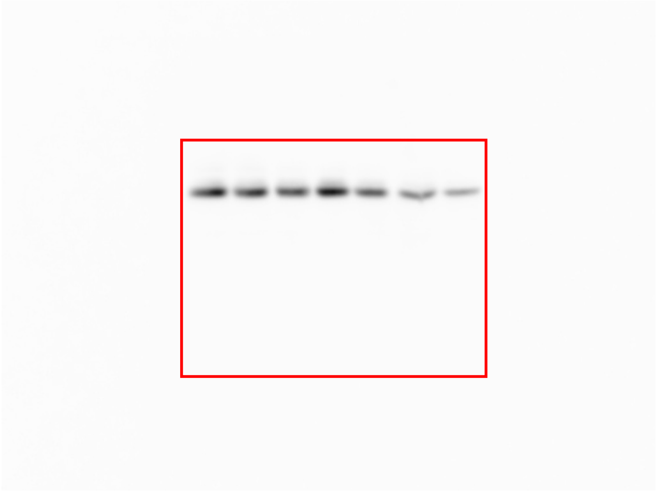

Alantolactone (μM)

0 1 5 10 15 20 25

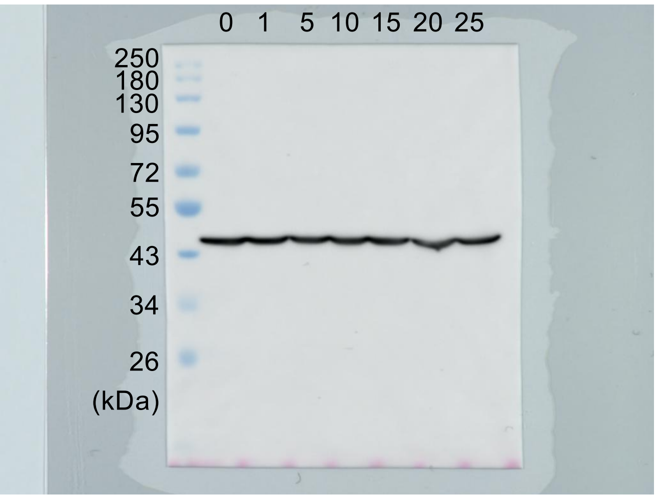

WB: β-Actin (reprobed)

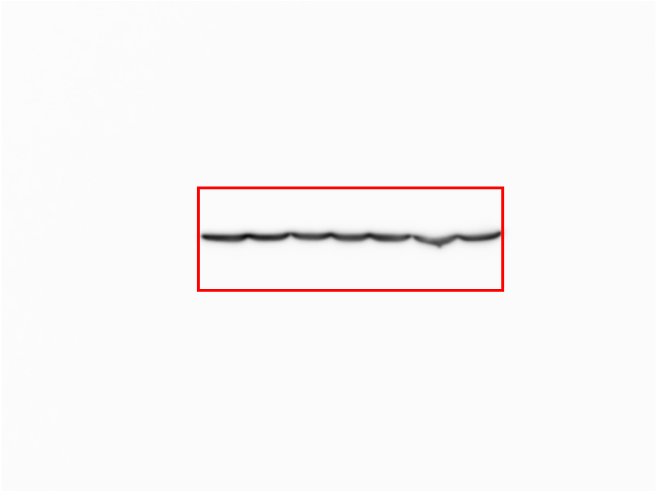

Figure S26: Original blots (1) in Figure 5B,C

Alantolactone (µM)

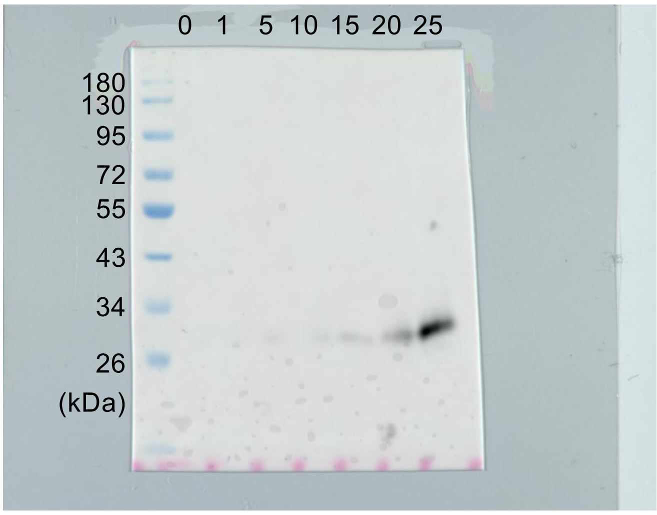

WB: TNF-R1

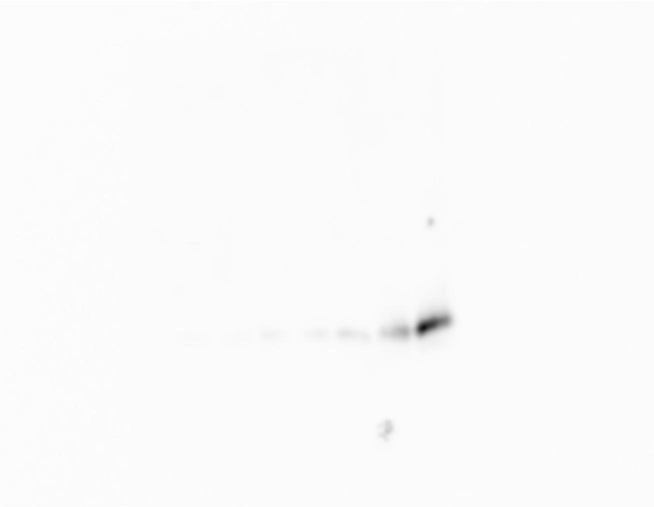

Alantolactone (µM)

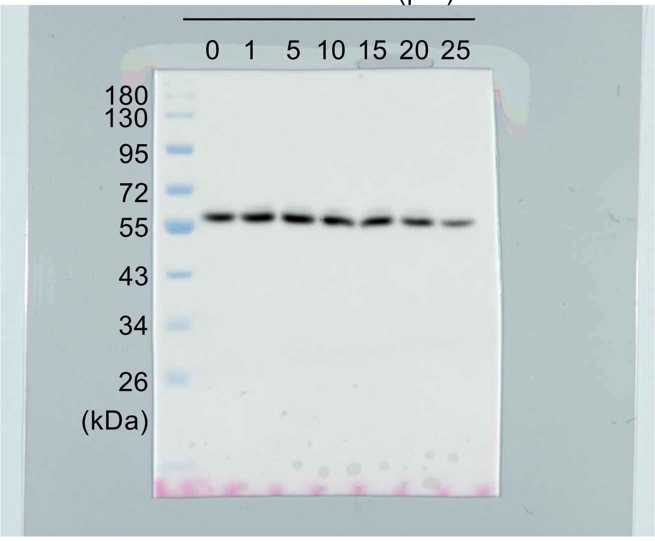

WB: TNF-R1

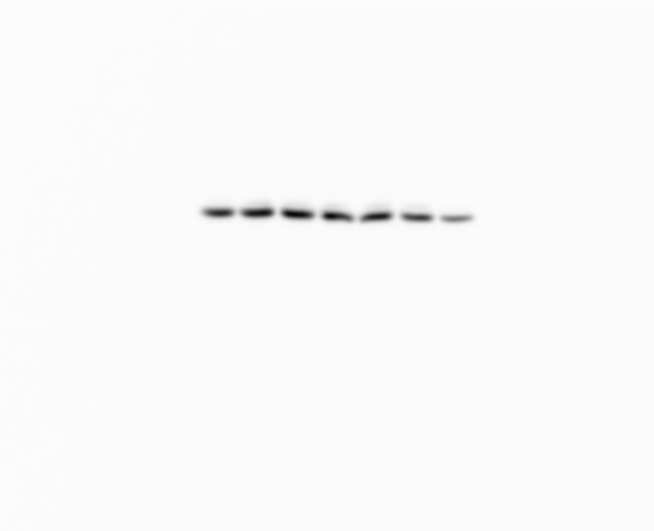

Alantolactone (µM)

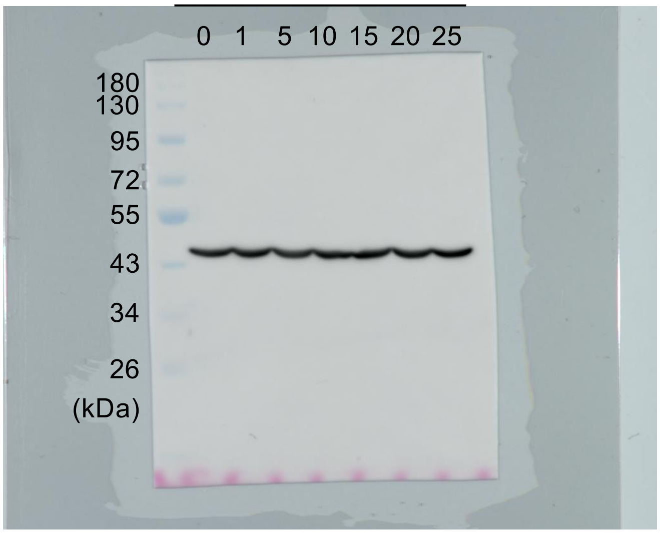

WB: β-Actin (reprobed)

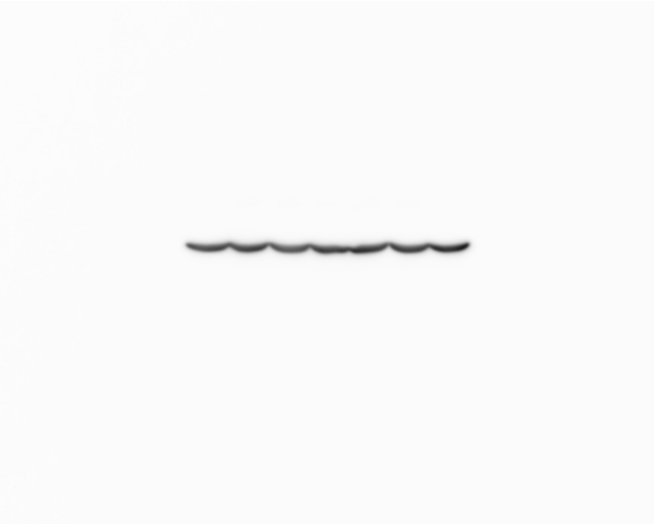

Figure S27: Original blots (2) in Figure 5B,C

Alantolactone (μM)

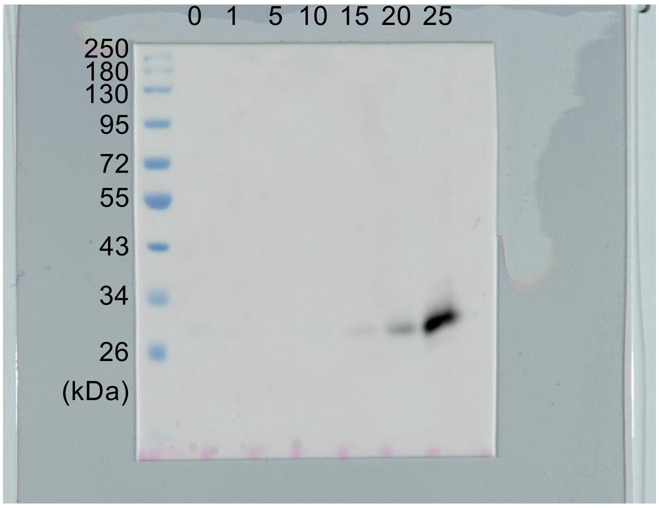

WB: TNF-R1

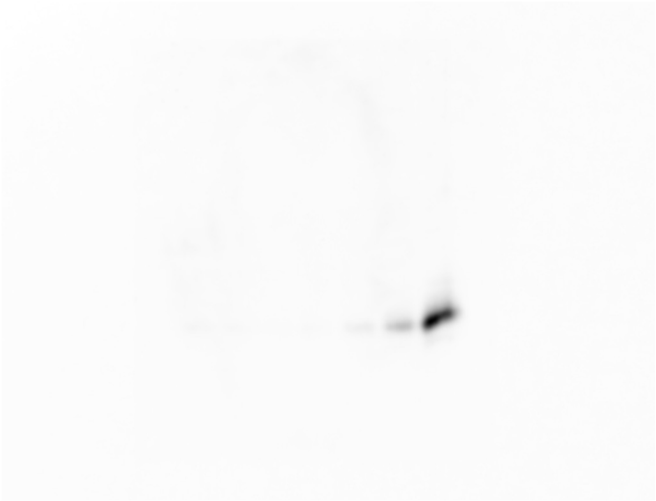

Alantolactone (μM)

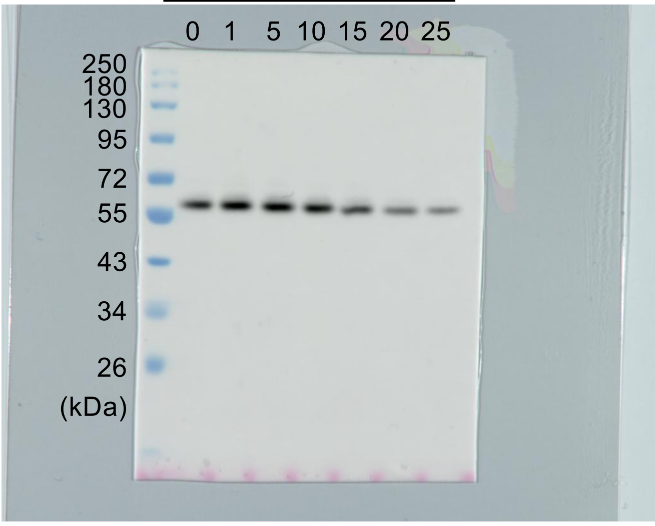

WB: TNF-R1

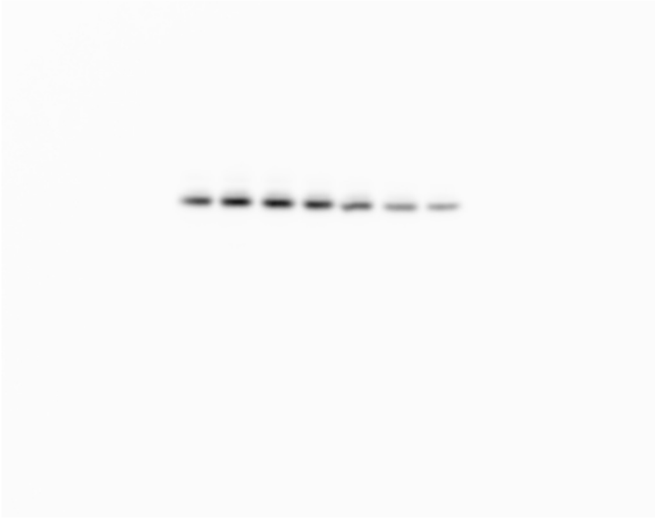

Alantolactone (μM)

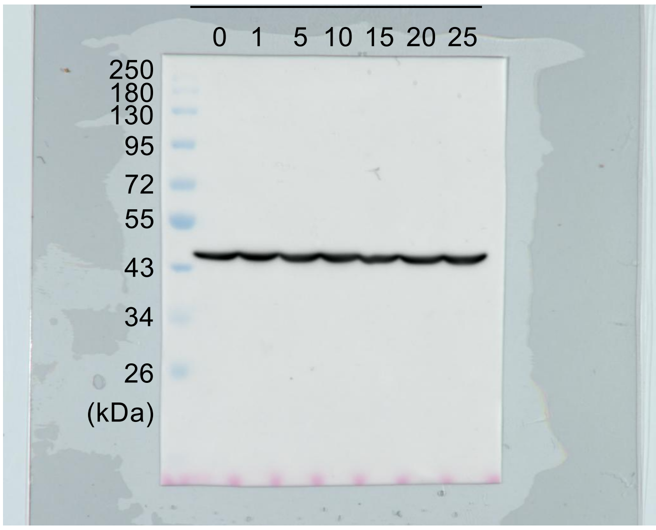

WB: β-Actin (reprobed)

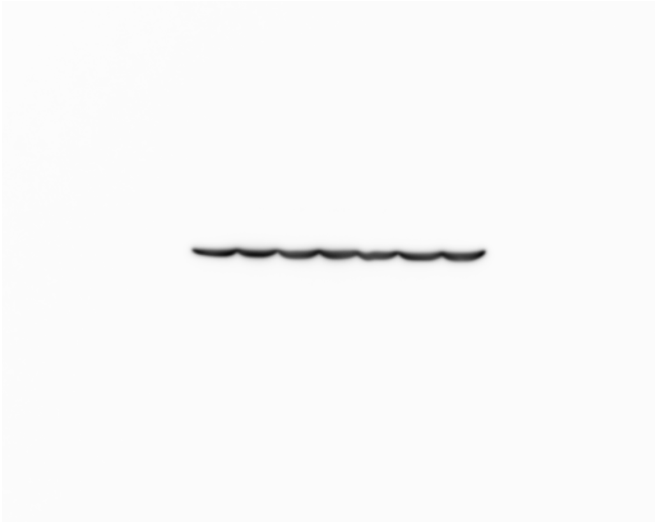

Figure S28: Original blots (3) in Figure 5B,C

Alantolactone (μM)

WB: TNF-R1

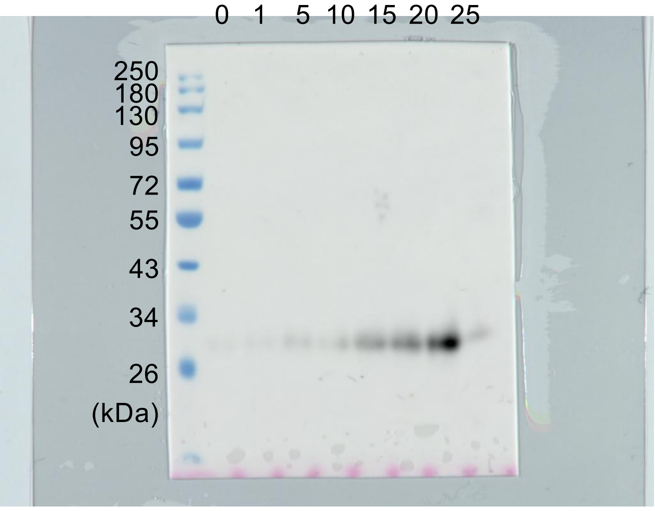

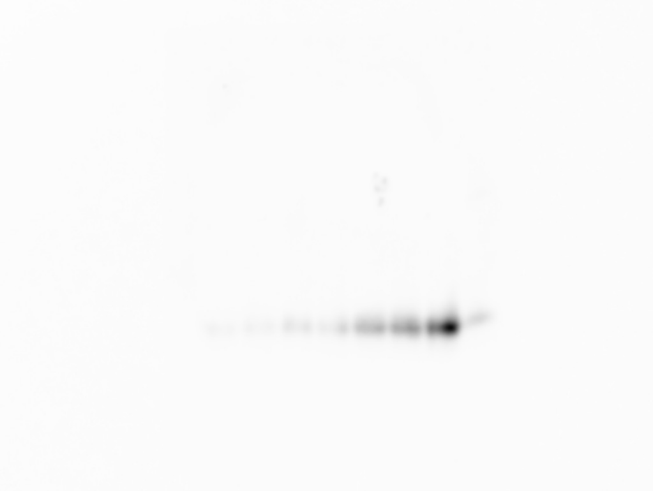

Alantolactone (μM)

WB: TNF-R1

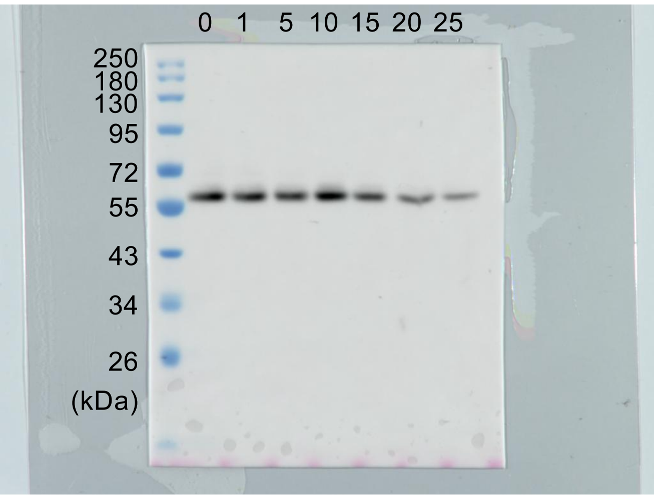

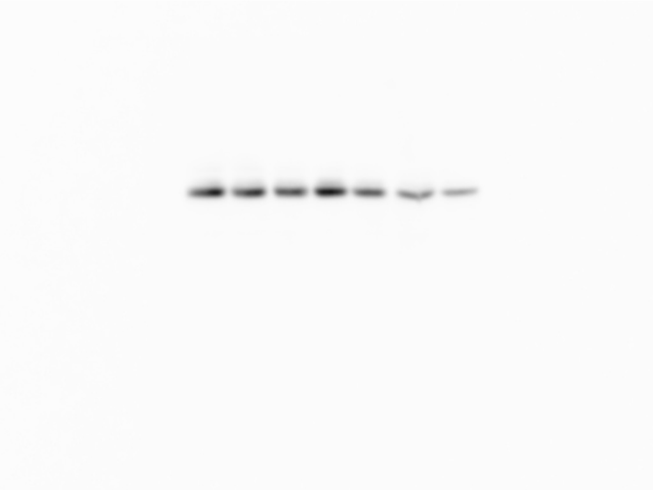

Alantolactone (μM)

WB: β-Actin (reprobed)

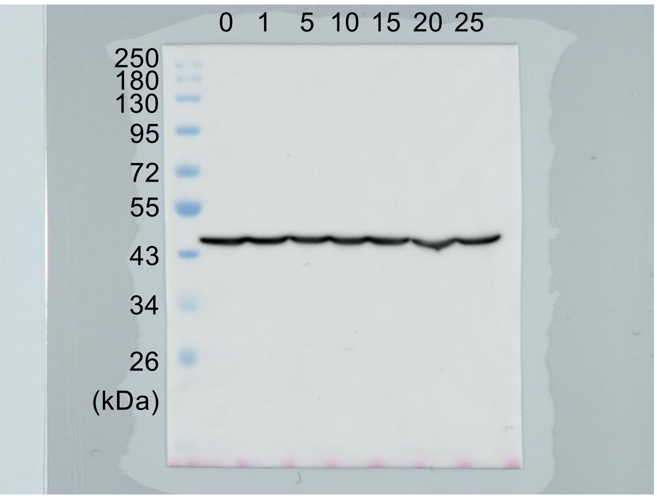

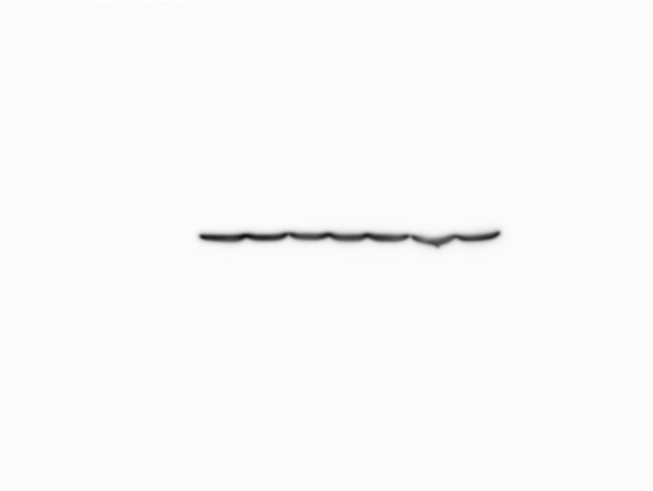

Figure S29: Original blots in Figure 6A

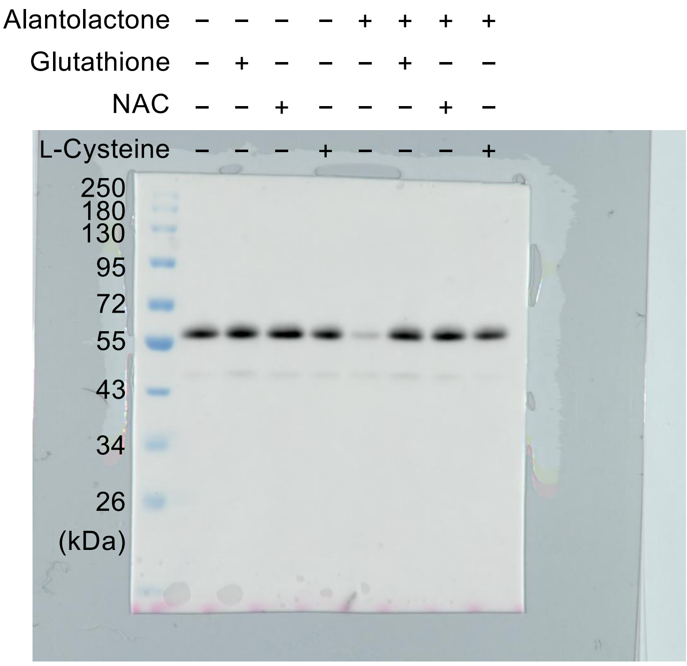

WB: TNF-R1

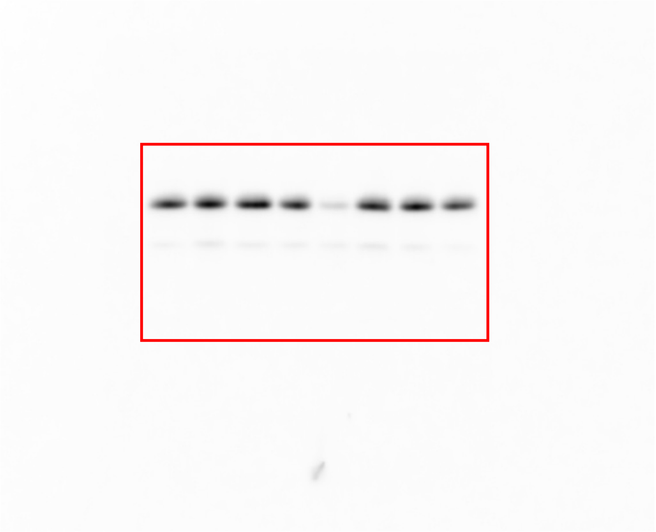

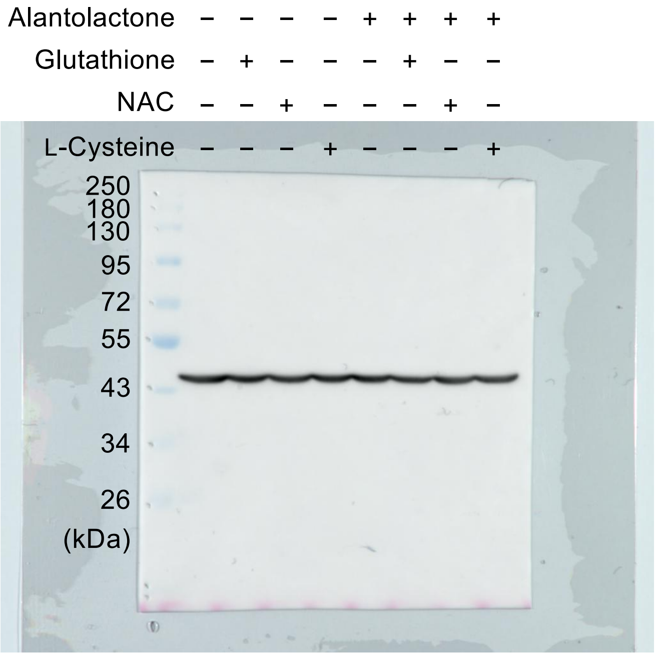

WB: β-Actin (reprobed)

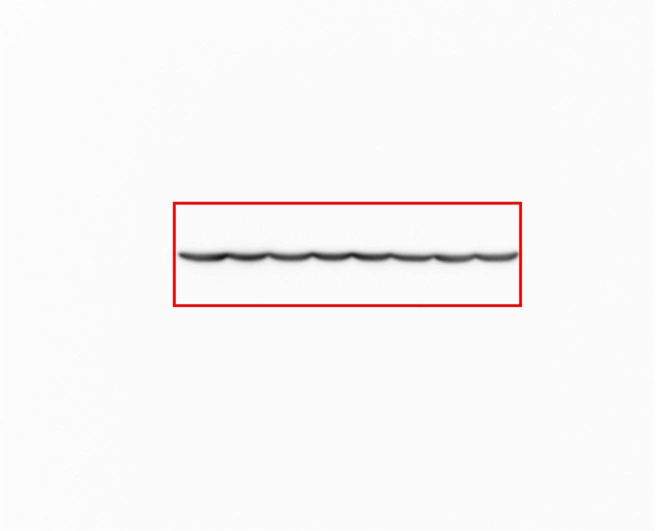

Figure S30: Original blots (1) in Figure 6B

Alantolactone	−	−	−	−	+	+	+	+
Glutathione	−	+	−	−	−	+	−	−
NAC	−	−	+	−	−	−	+	−

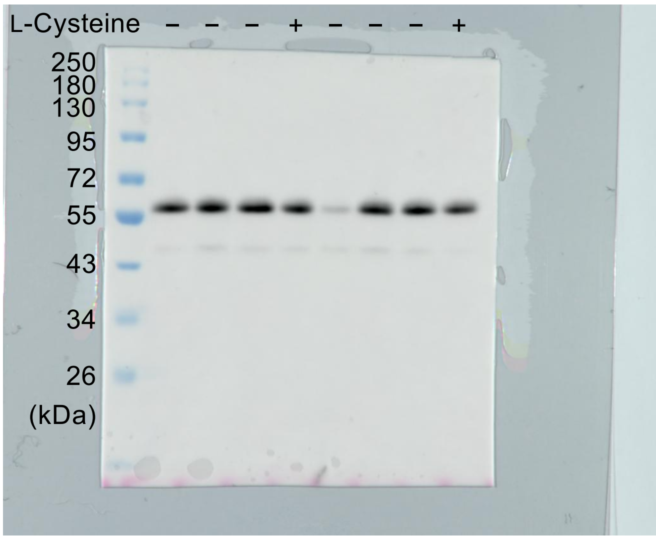

WB: TNF-R1

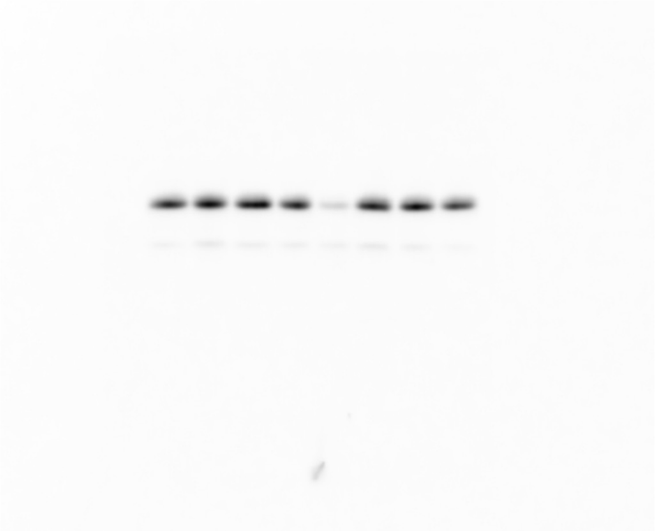

Alantolactone	−	−	−	−	+	+	+	+
Glutathione	−	+	−	−	−	+	−	−
NAC	−	−	+	−	−	−	+	−

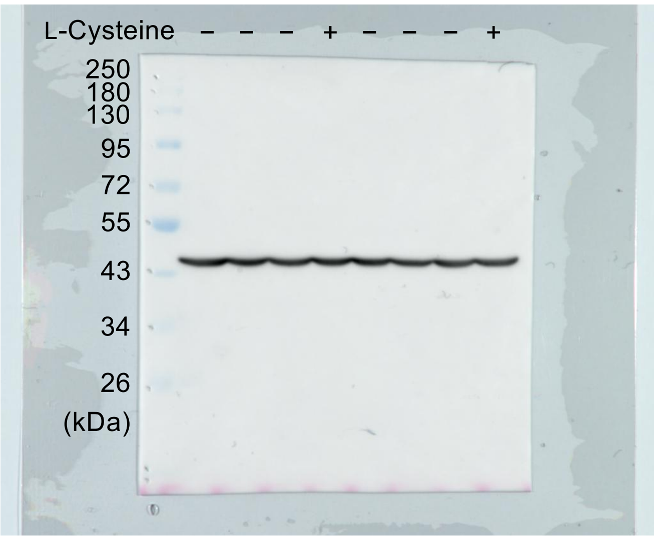

WB: β-Actin (reprobed)

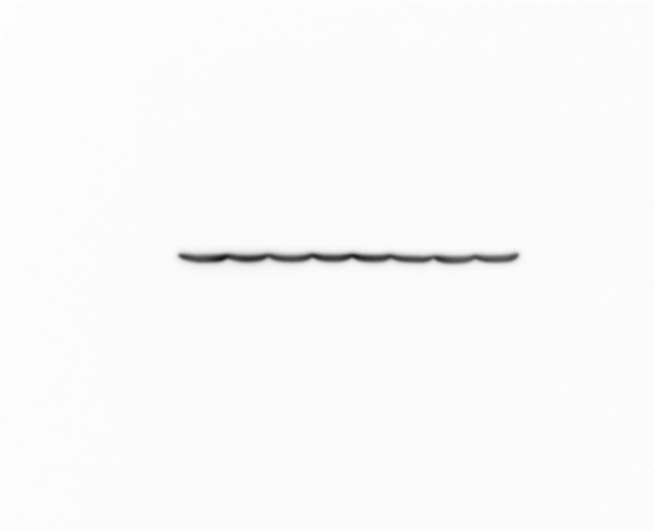

Figure S31: Original blots (2) in Figure 6B

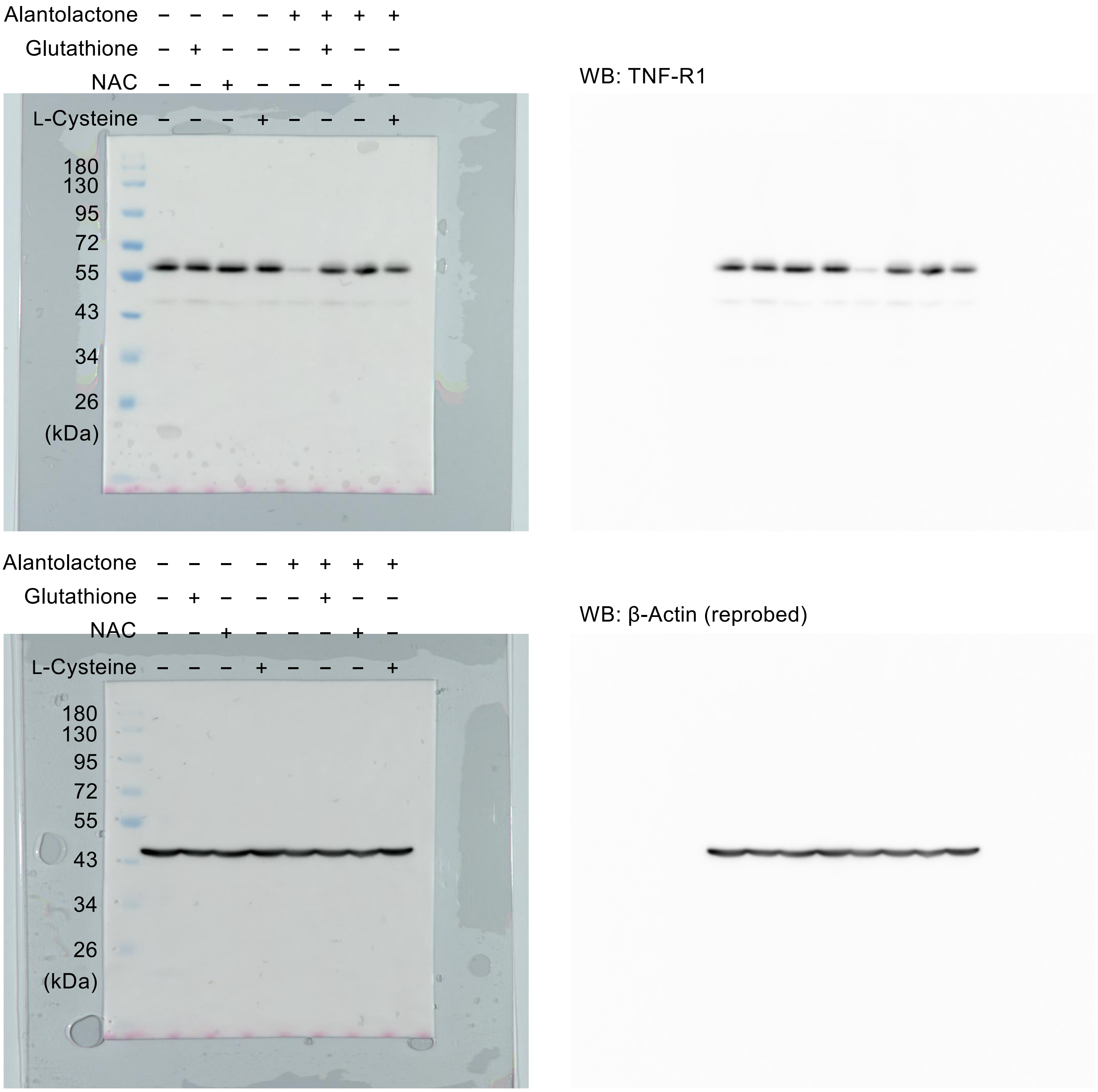

Figure S32: Original blots (3) in Figure 6B

Alantolactone – – – – + + + +
Glutathione – + – – – + – –
NAC – – + – – – + –

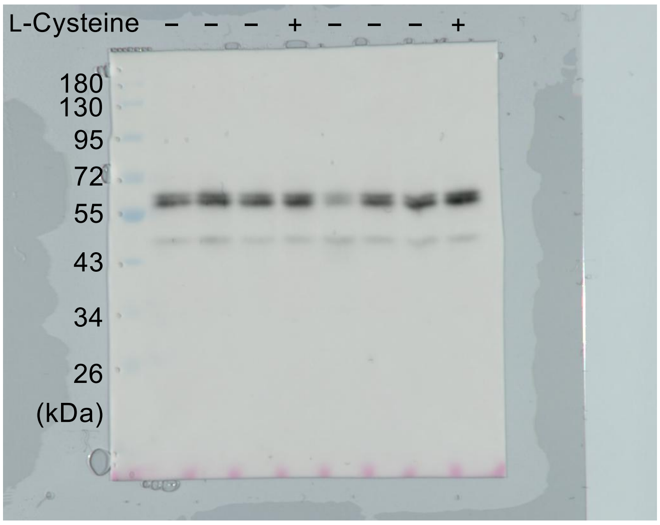

WB: TNF-R1

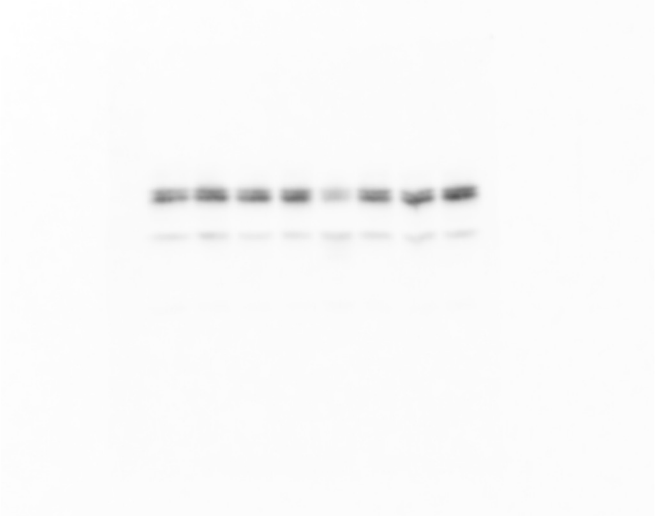

Alantolactone – – – – + + + +
Glutathione – + – – – + – –
NAC – – + – – – + –

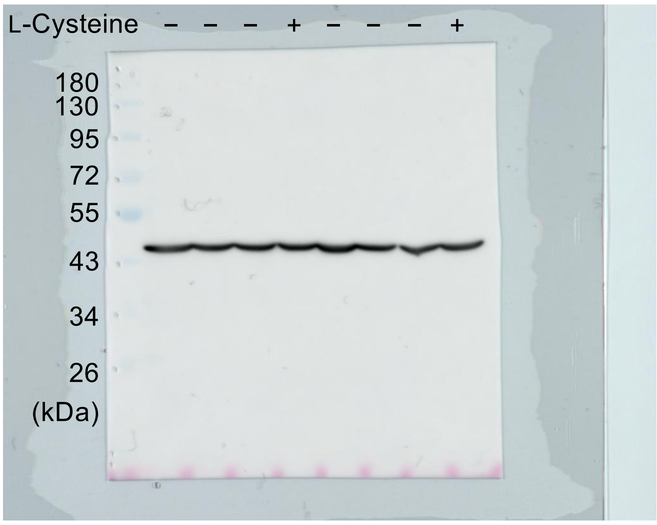

WB: β-Actin (reprobed)

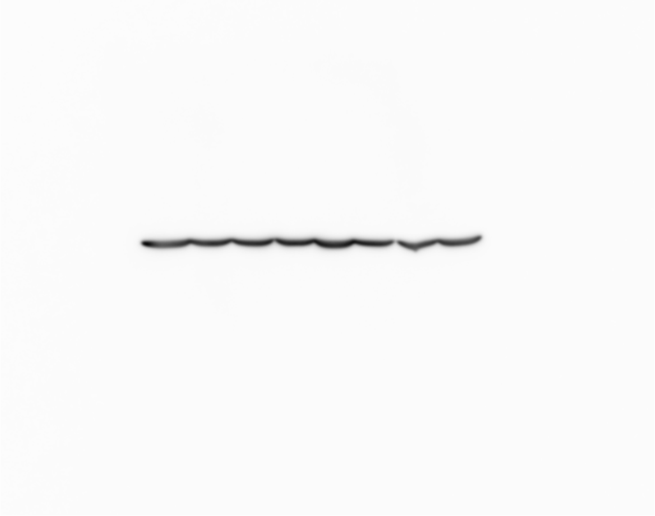

Figure S33: Original blots in Figure 8A

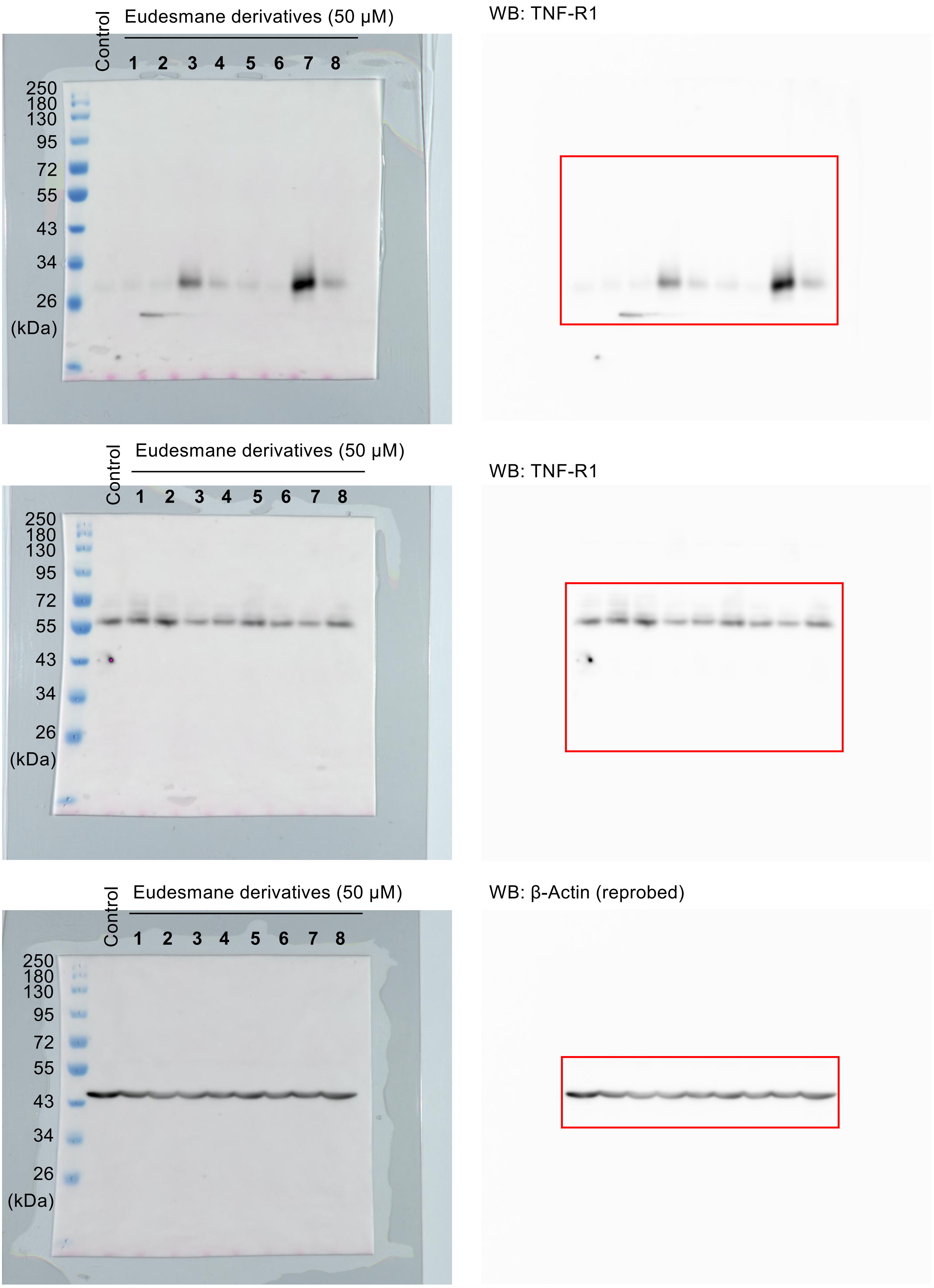

Figure S34: Original blots (1) in Figure 8B,C

Control Eudesmane derivatives (50 µM)

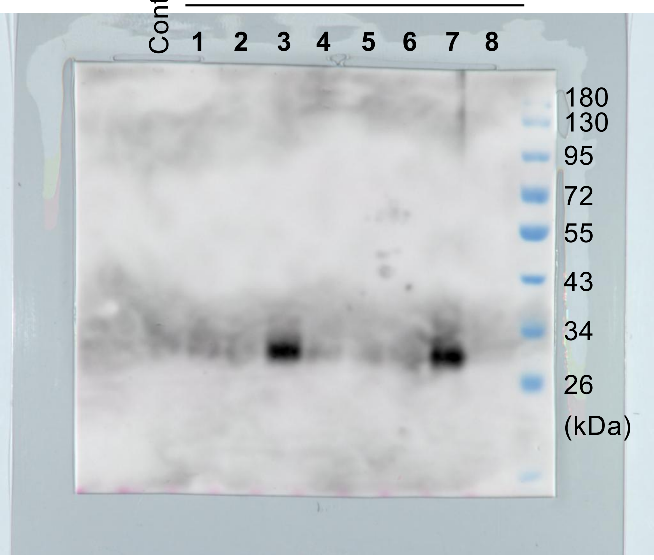

WB: TNF-R1

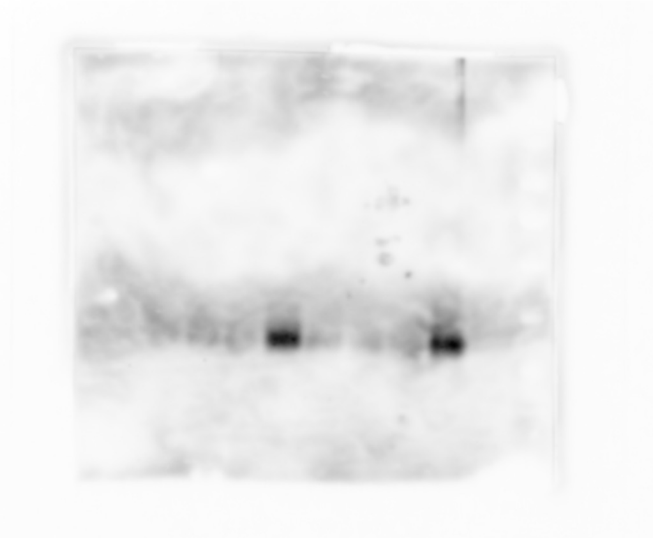

Control Eudesmane derivatives (50 µM)

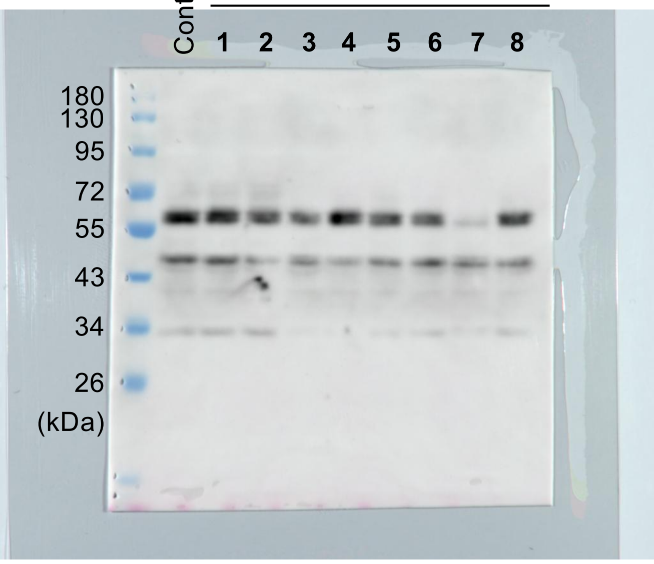

WB: TNF-R1

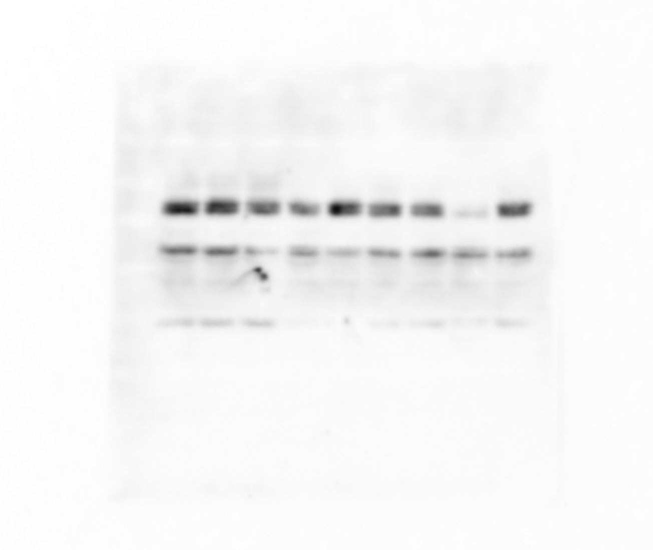

Control Eudesmane derivatives (50 µM)

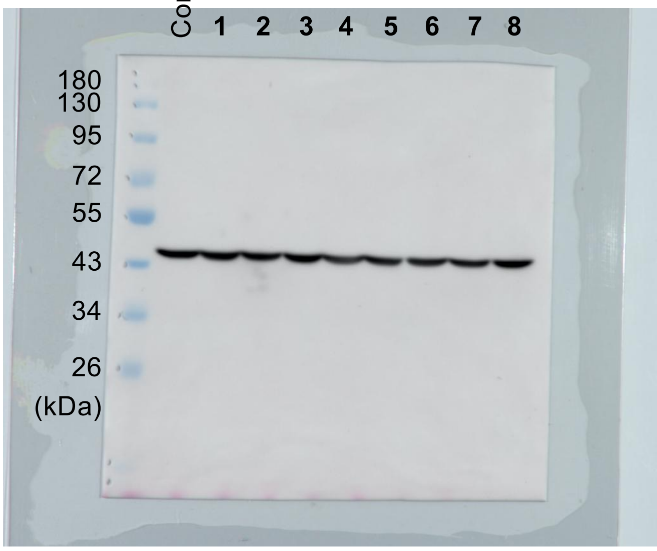

WB: β-Actin (reprobed)

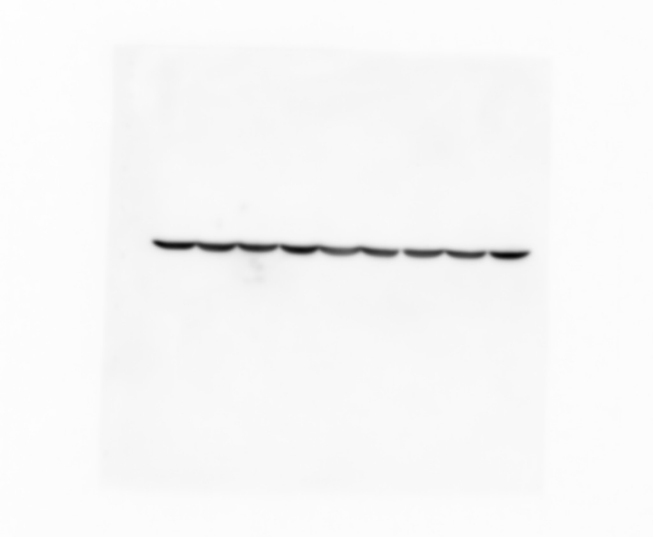

Figure S35: Original blots (2) in Figure 8B,C

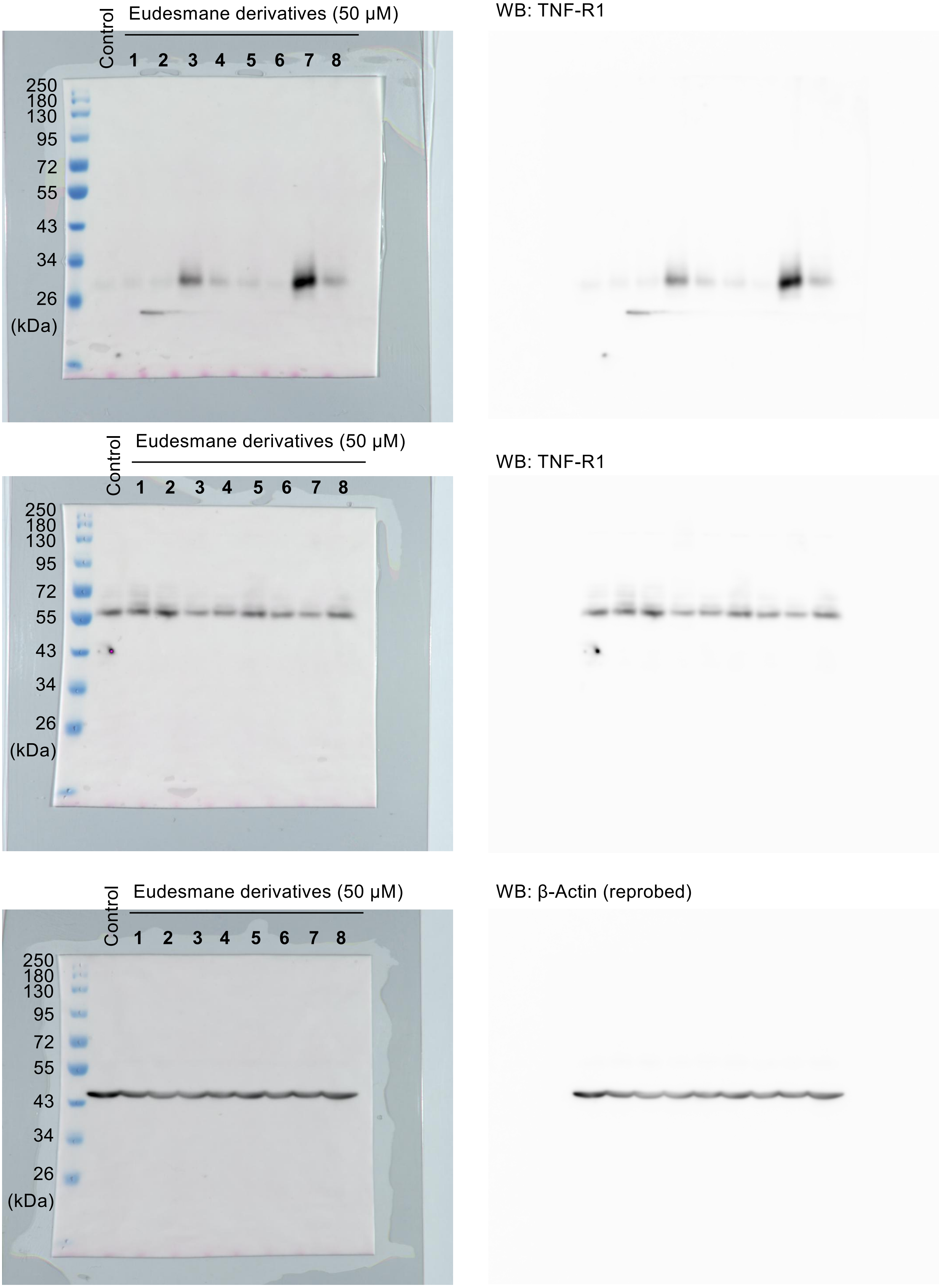

Figure S36: Original blots (3) in Figure 8B,C

Control Eudesmane derivatives (50 µM)

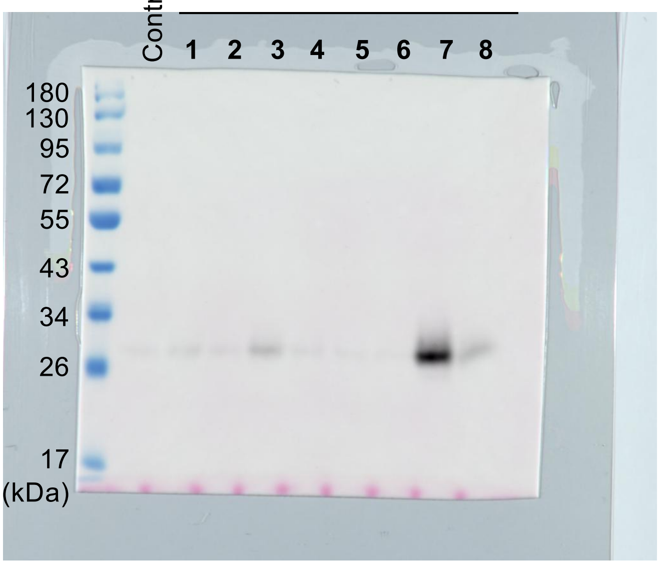

WB: TNF-R1

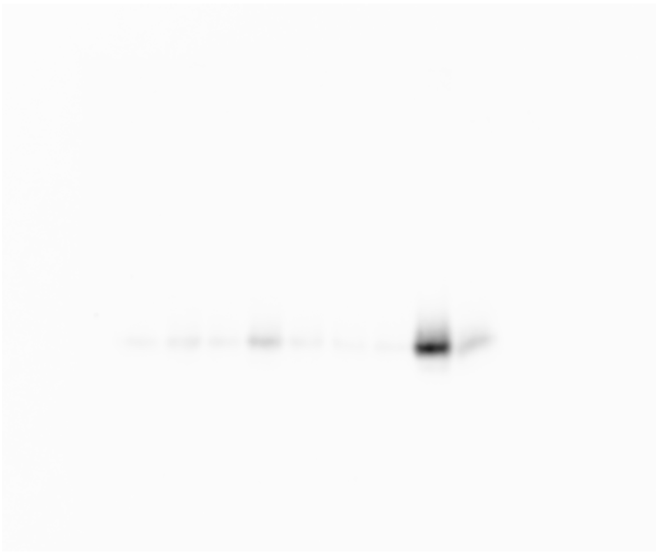

Control Eudesmane derivatives (50 µM)

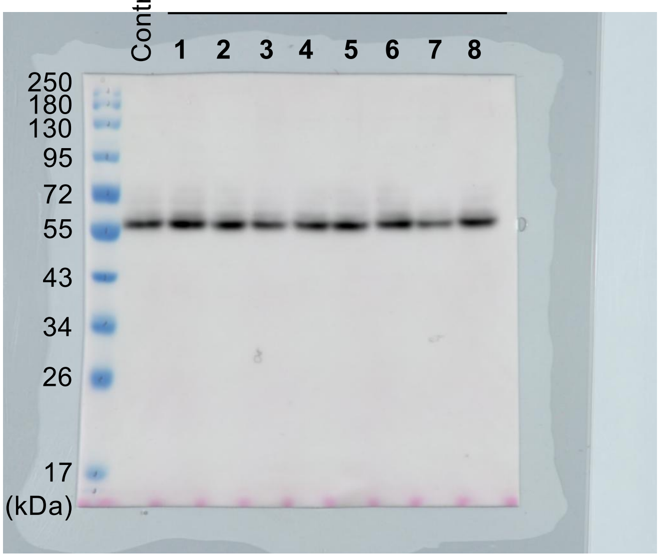

WB: TNF-R1

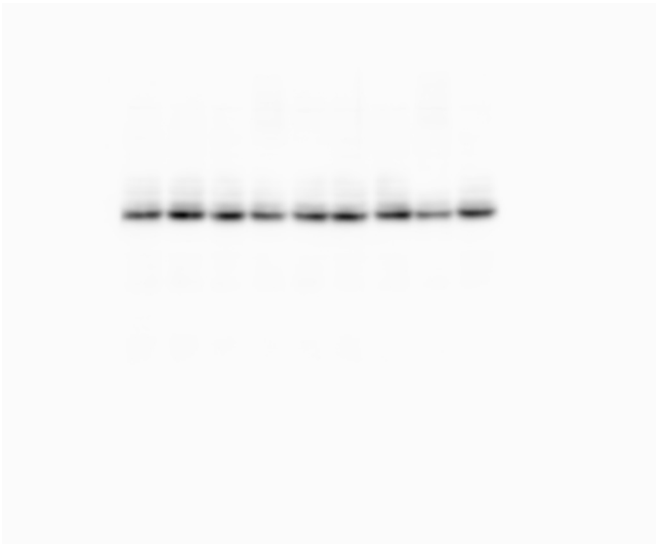

Control Eudesmane derivatives (50 µM)

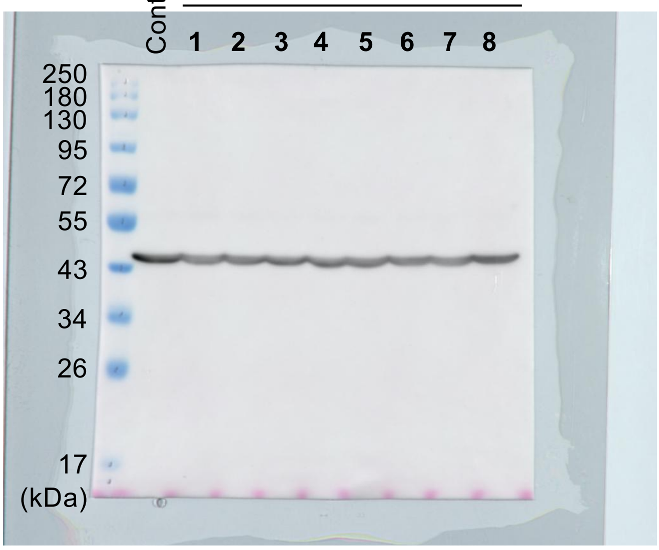

WB: β-Actin (reprobed)

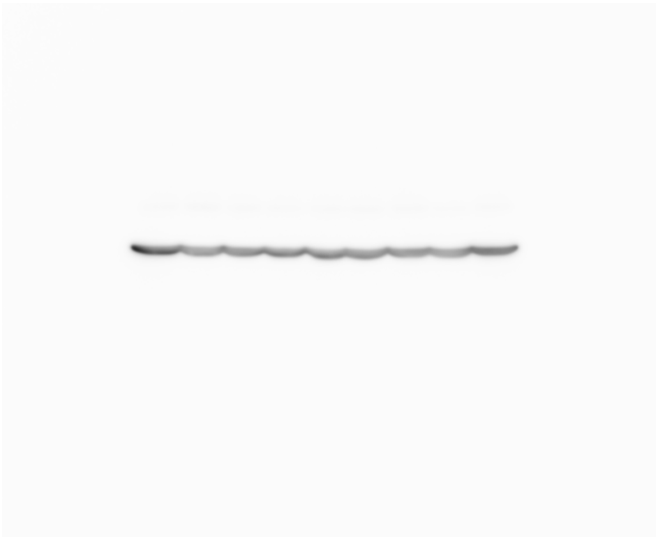

Figure S37: Original blots in Figure 8D

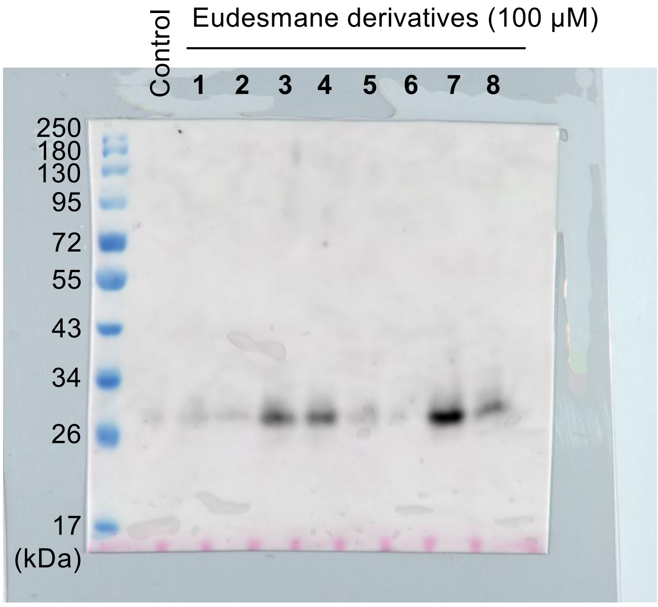

WB: TNF-R1

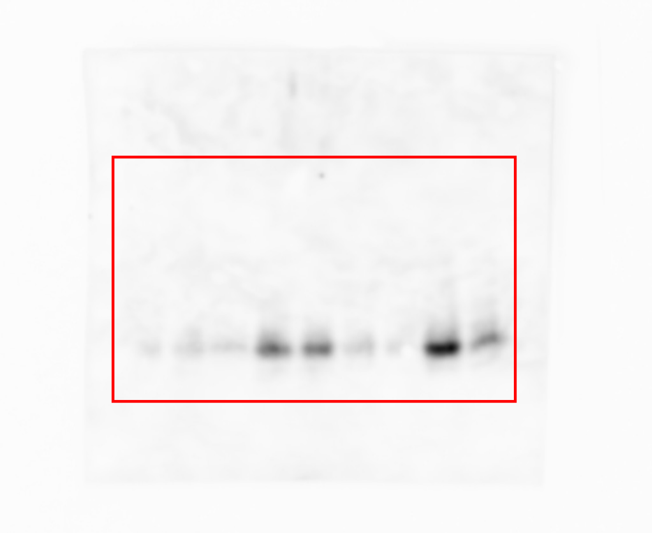

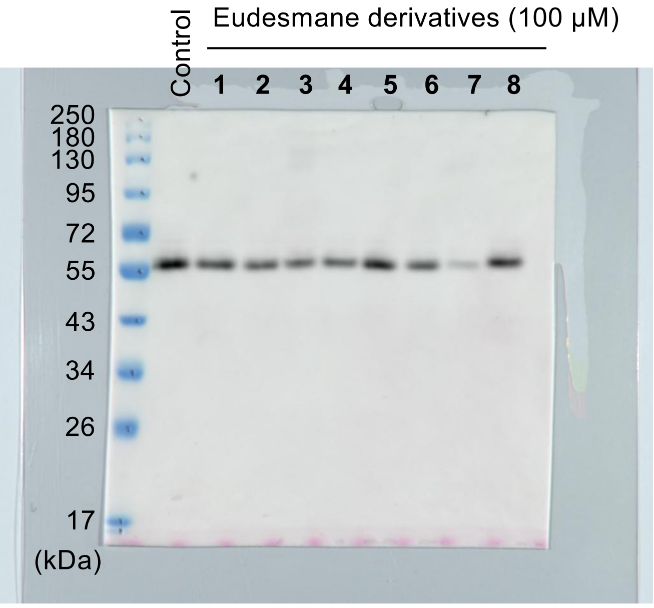

WB: TNF-R1

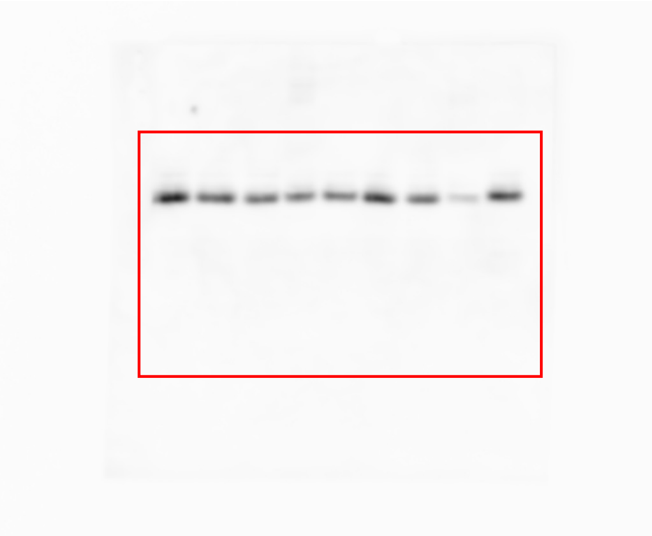

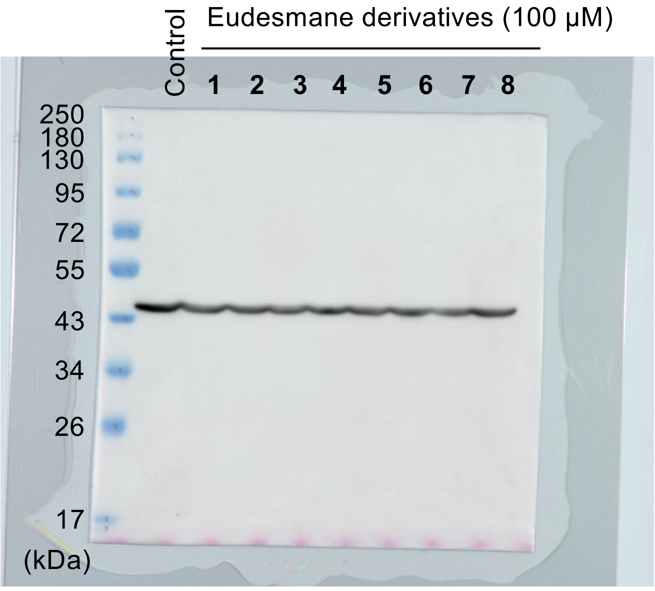

WB: β-Actin (reprobed)

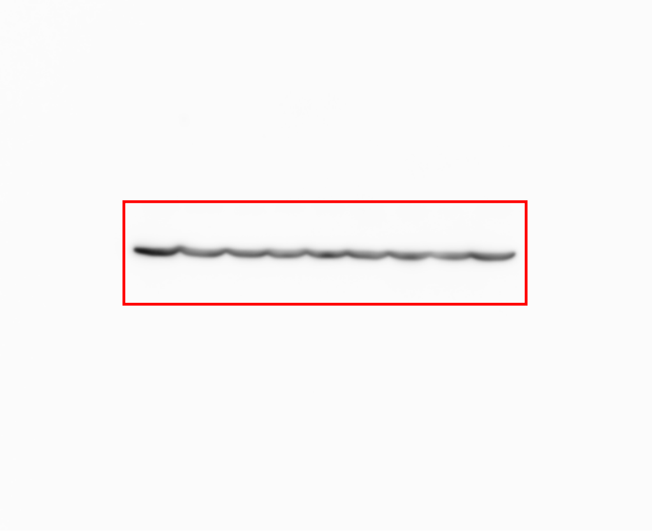

Figure S38: Original blots (1) in Figure 8E,F

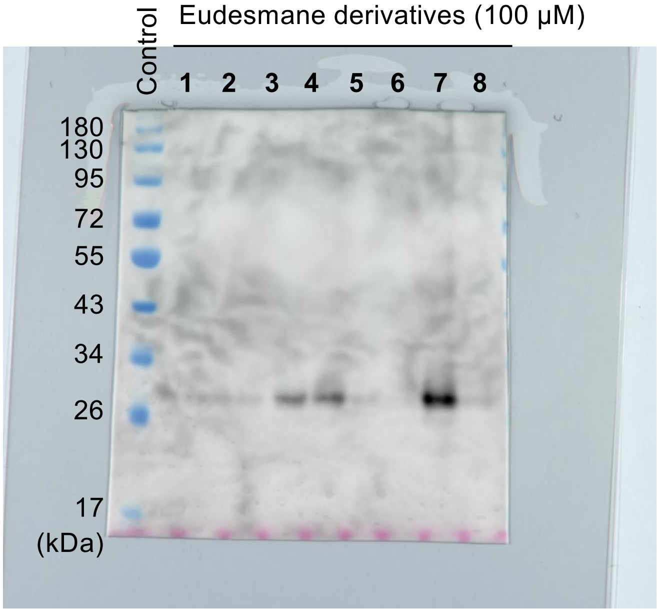

WB: TNF-R1

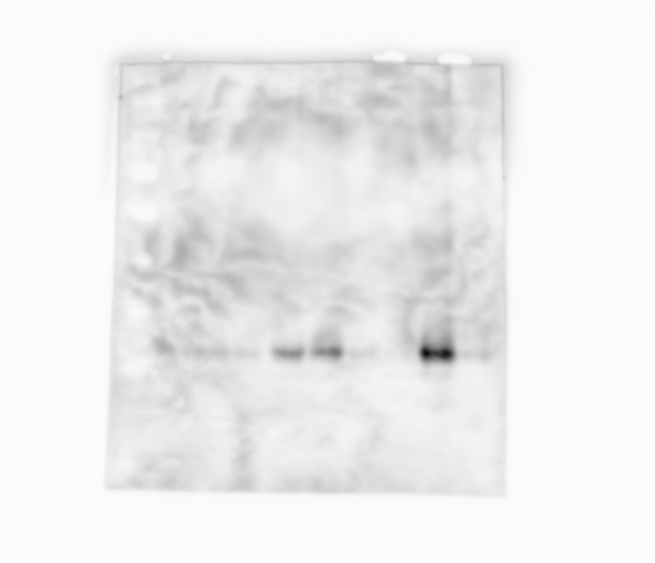

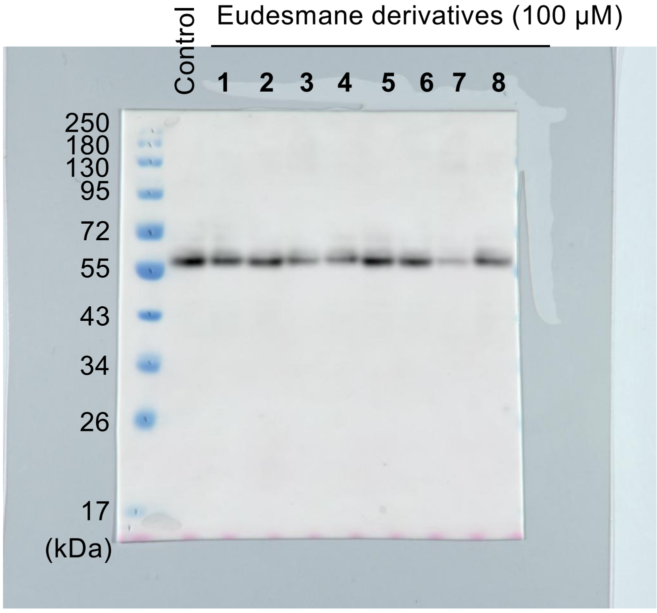

WB: TNF-R1

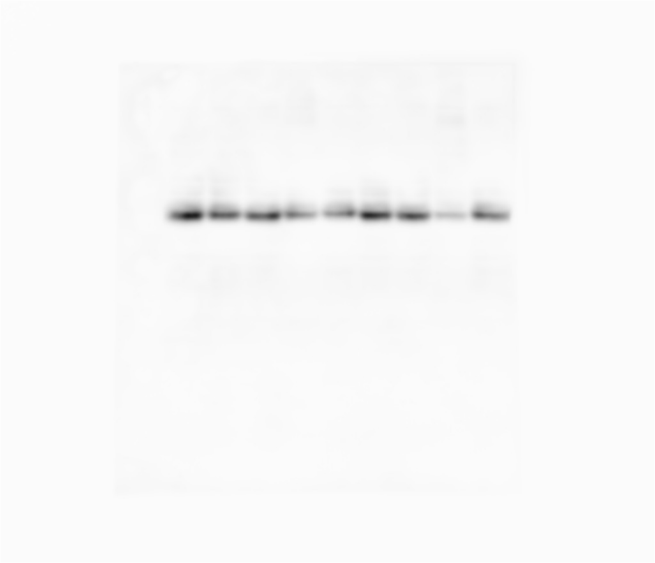

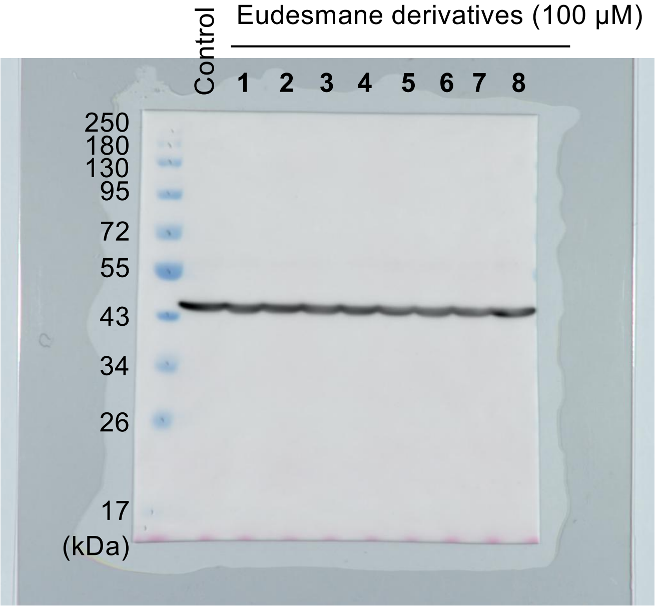

WB: β-Actin (reprobed)

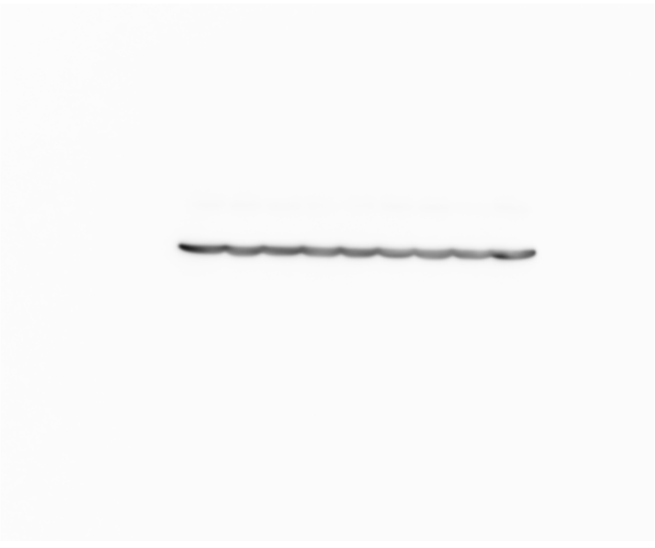

Figure S39: Original blots (2) in Figure 8E,F

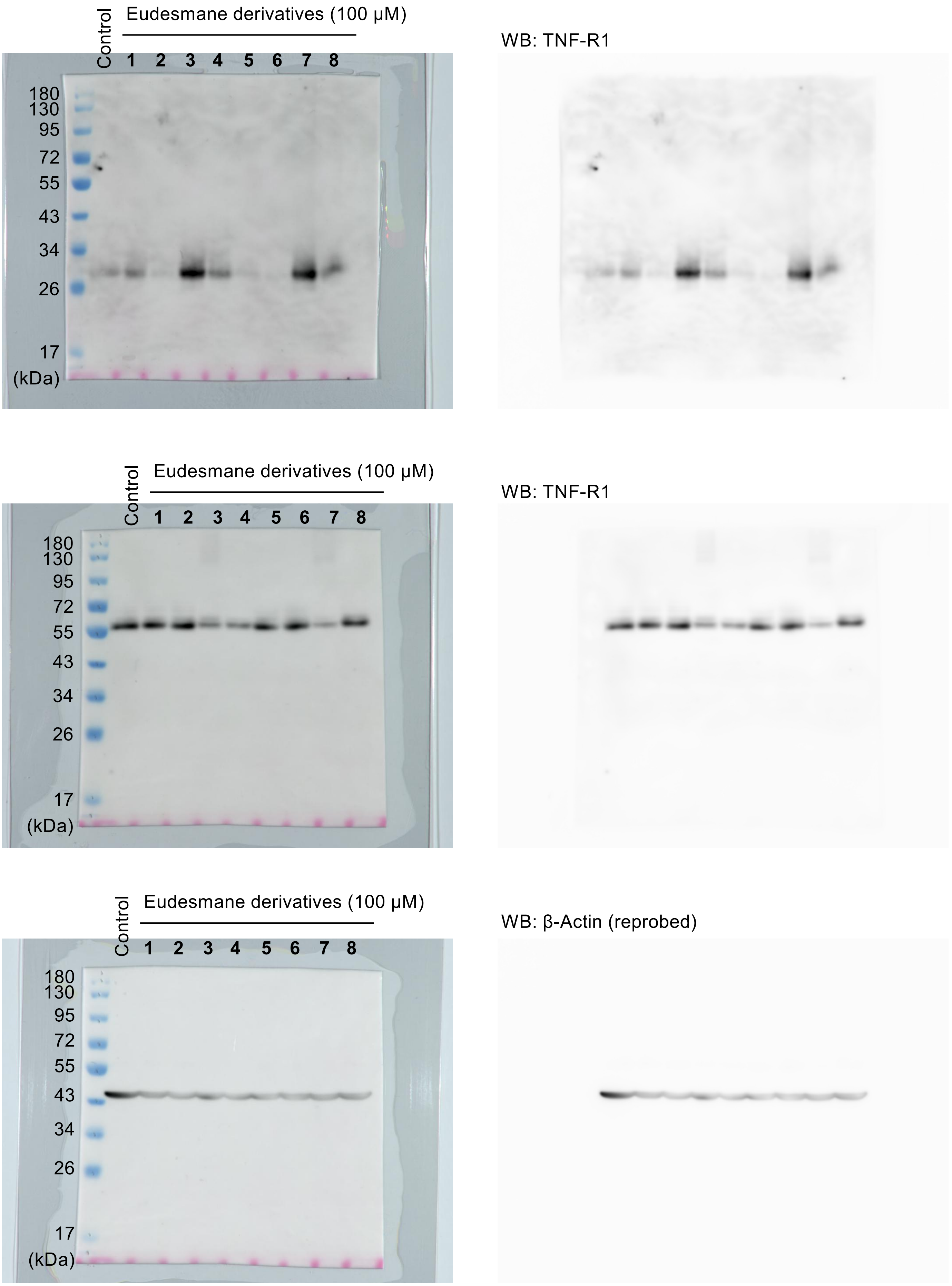

Figure S40: Original blots (3) in Figure 8E,F

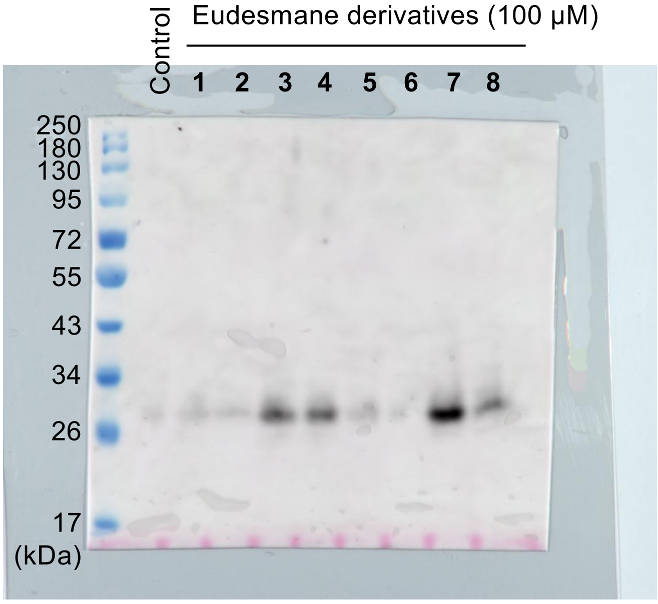

WB: TNF-R1

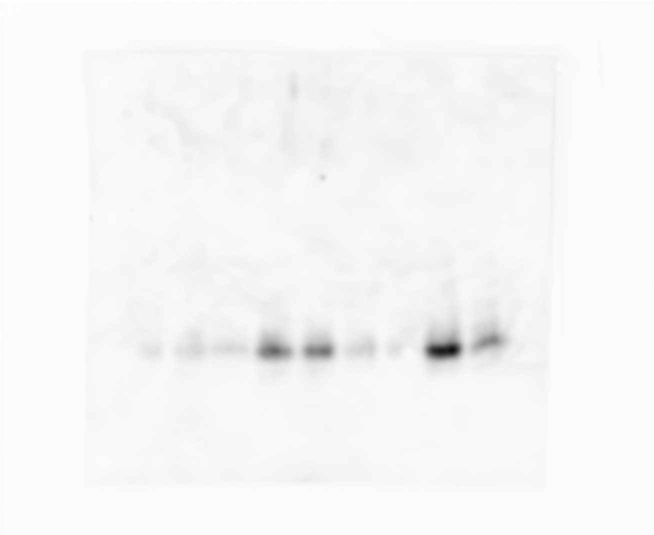

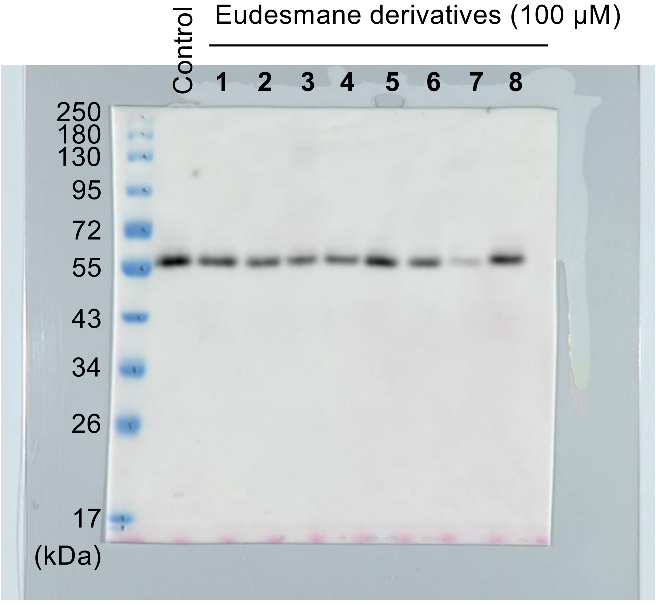

WB: TNF-R1

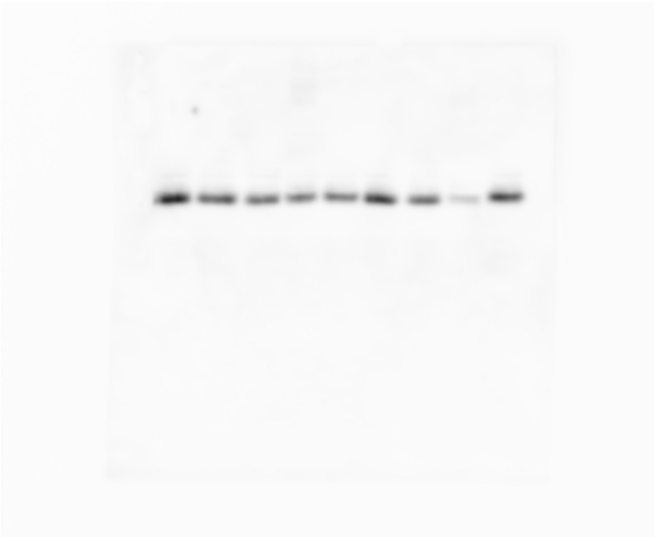

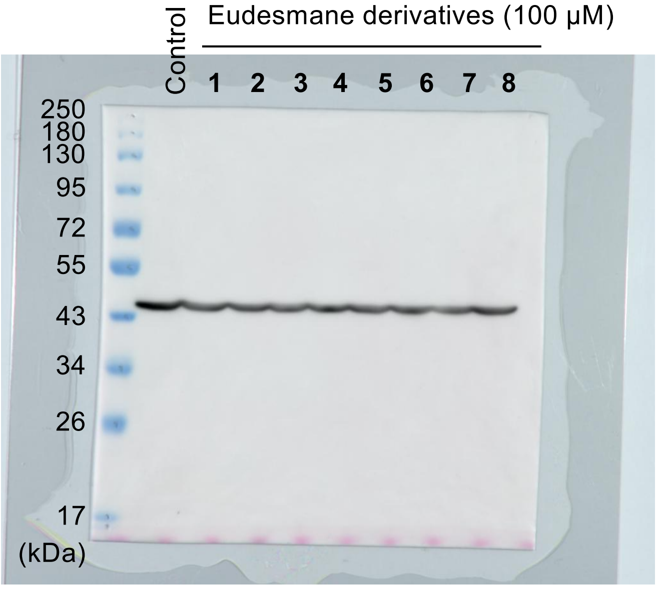

WB: β-Actin (reprobed)

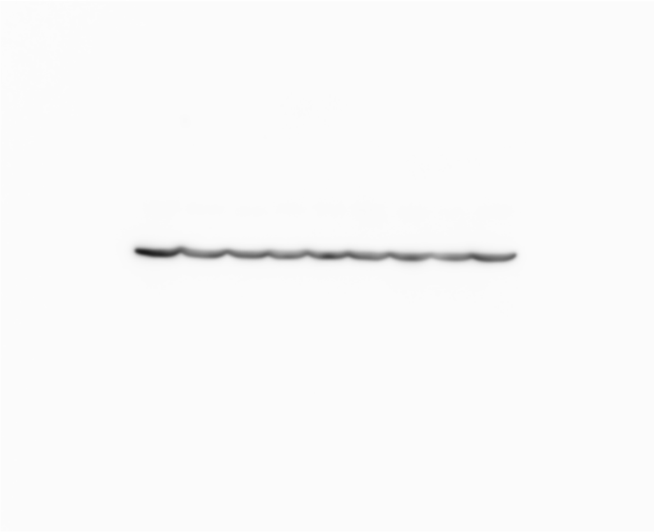

Figure S41: Original blots in Figure 9B

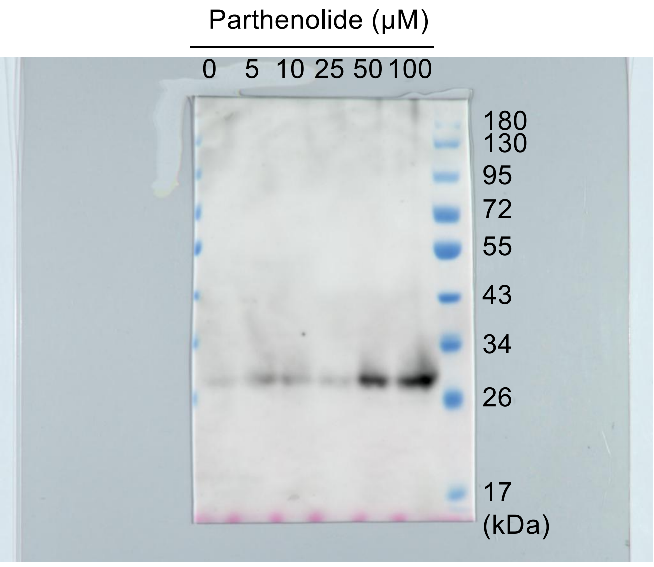

WB: TNF-R1

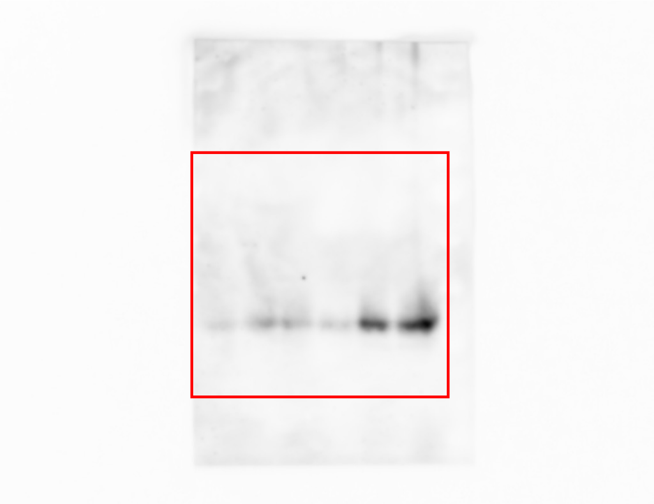

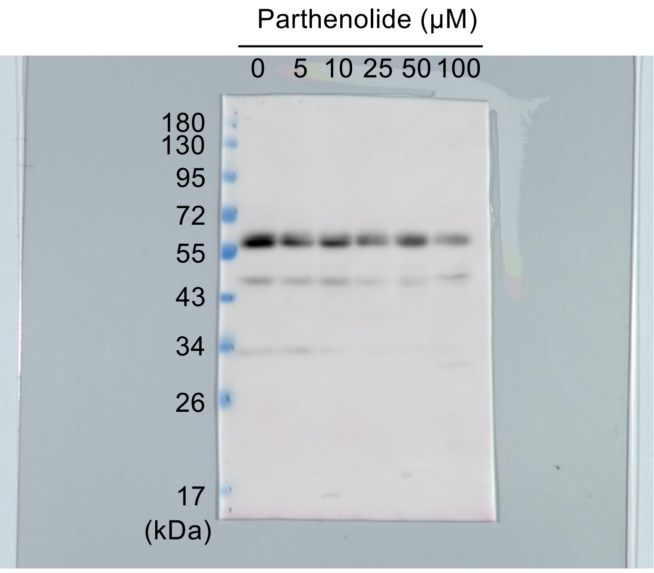

WB: TNF-R1

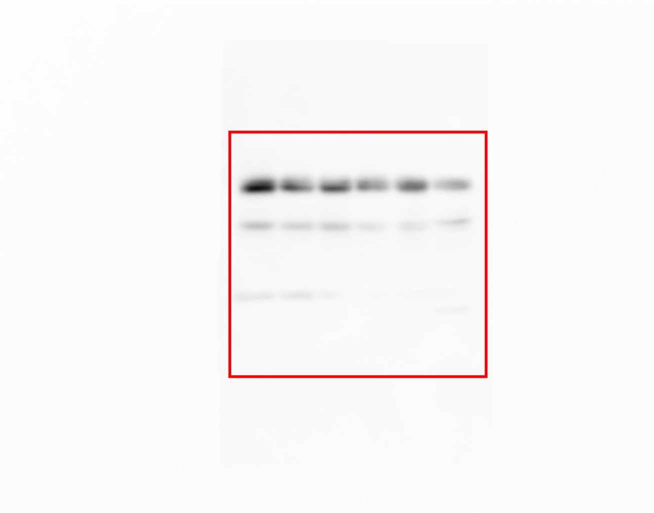

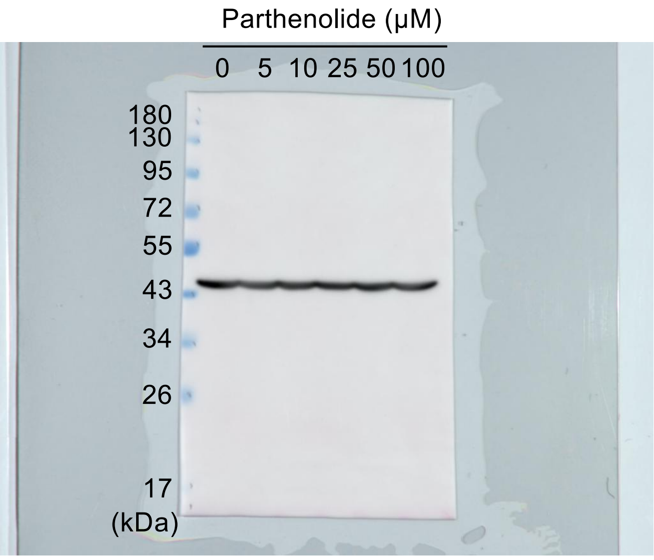

WB: β-Actin (reprobed)

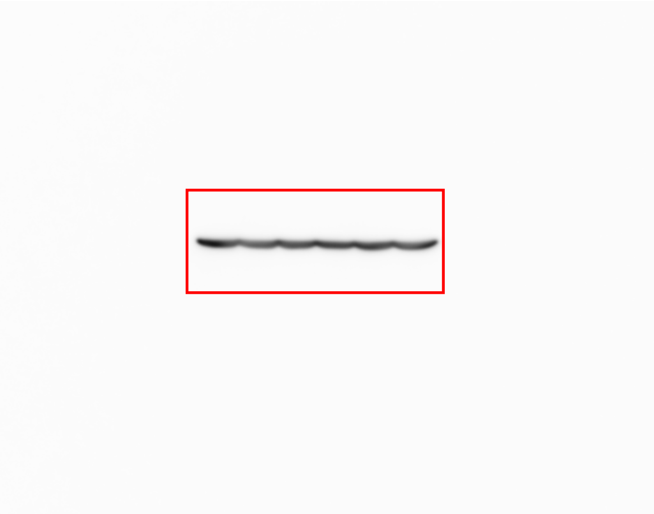

Figure S42: Original blots (1) in Figure 9C,D

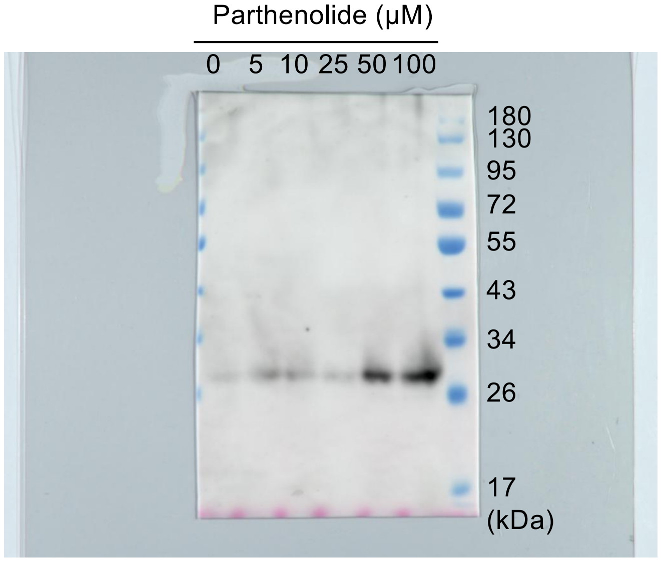

WB: TNF-R1

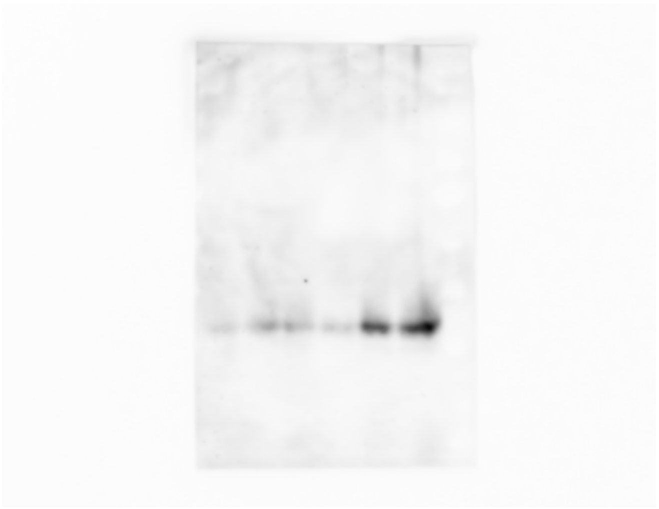

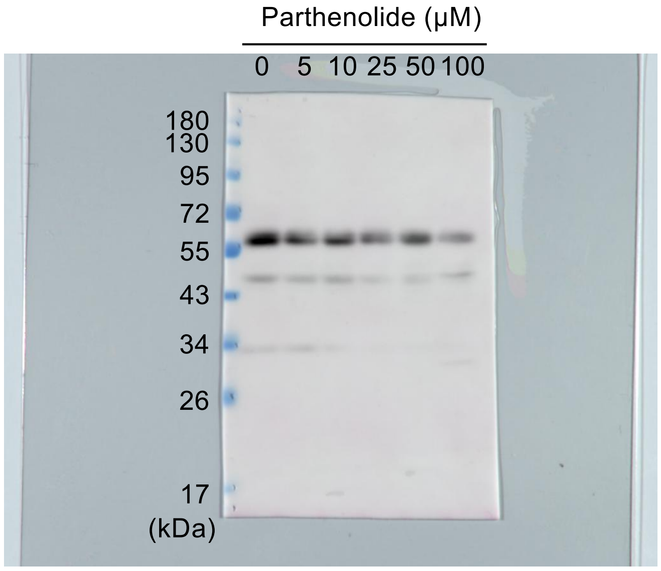

WB: TNF-R1

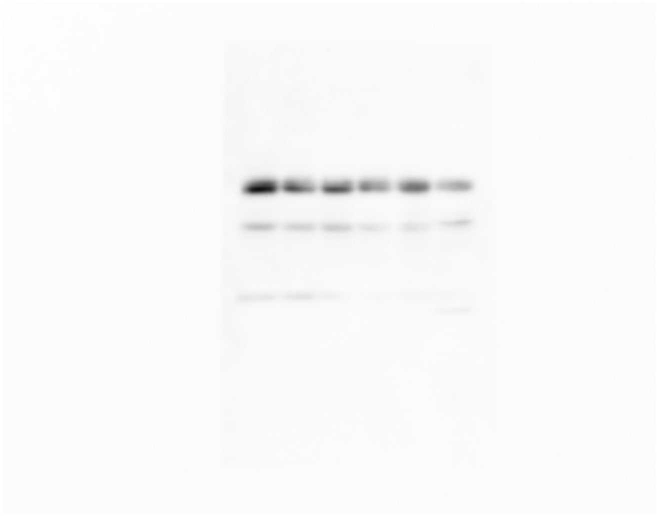

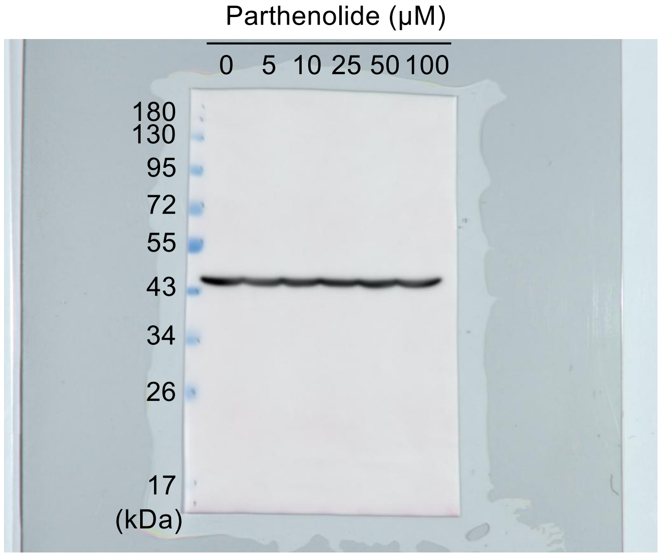

WB: β-Actin (reprobed)

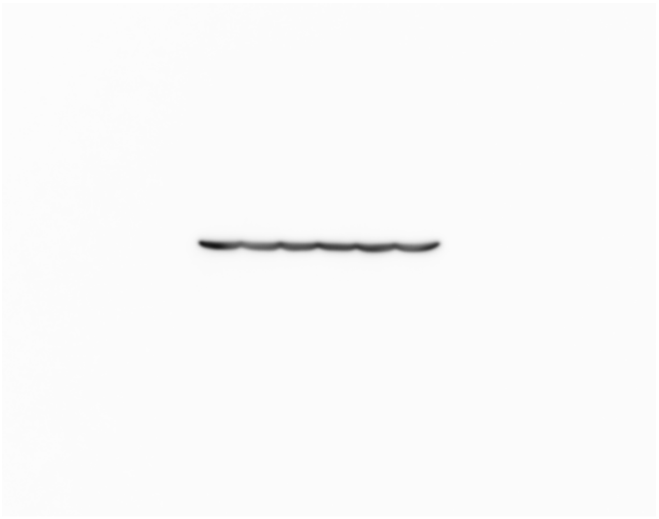

Figure S43: Original blots (2) in Figure 9C,D

Parthenolide (μM)

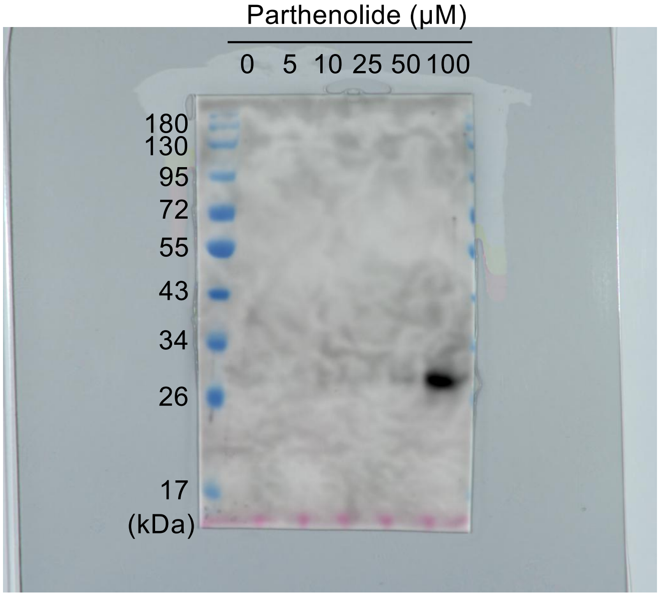

WB: TNF-R1

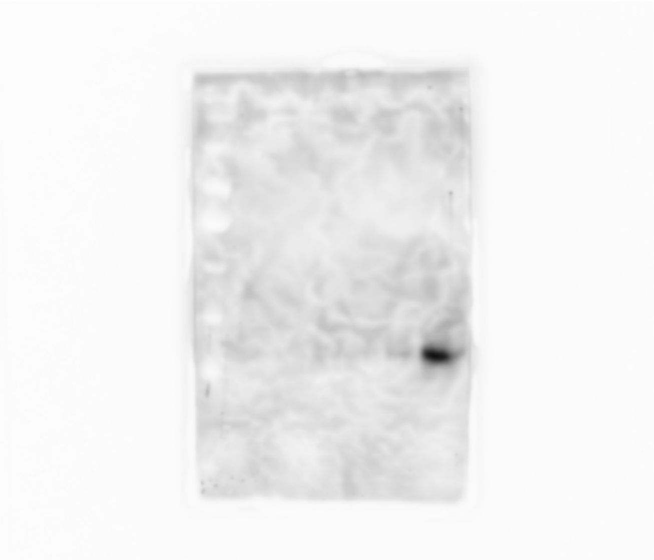

Parthenolide (μM)

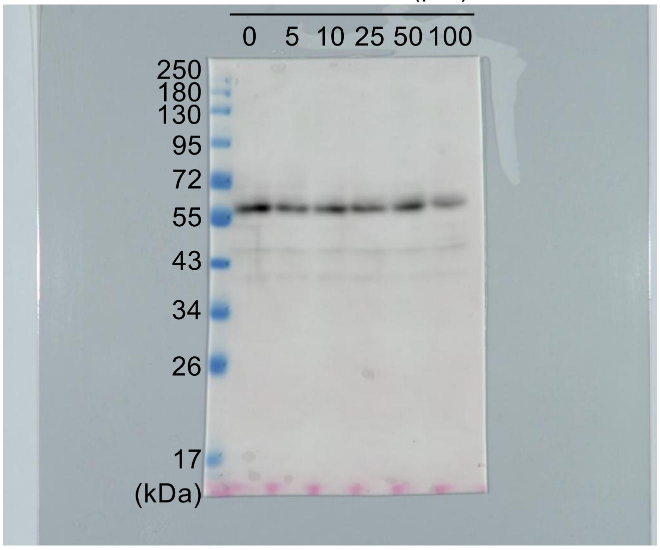

WB: TNF-R1

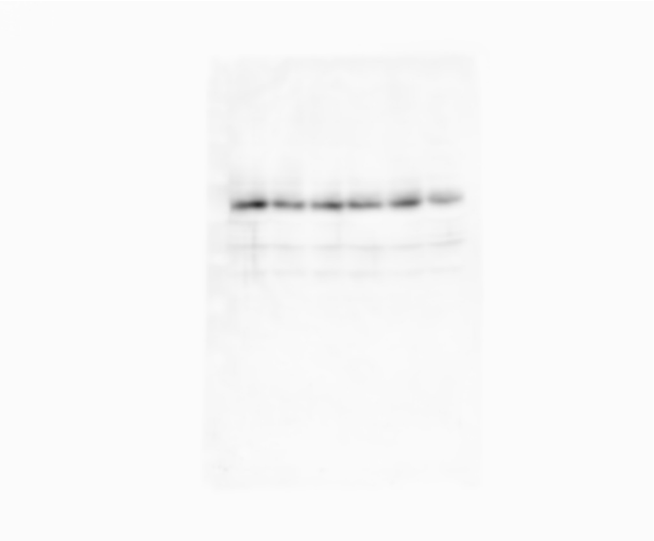

Parthenolide (μM)

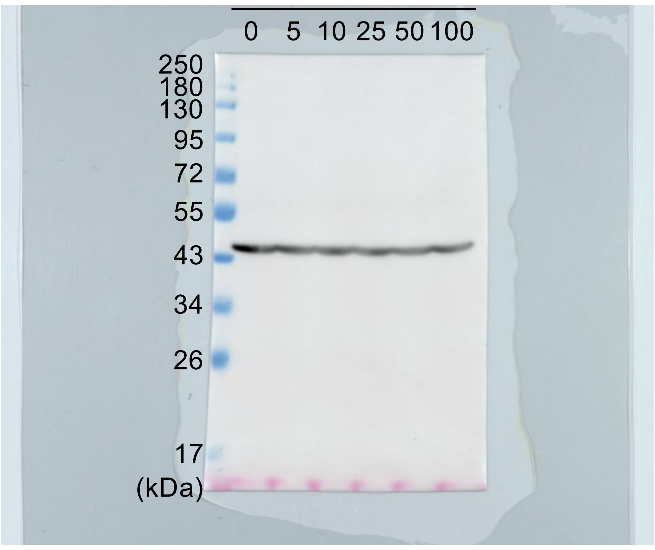

WB: β-Actin (reprobed)

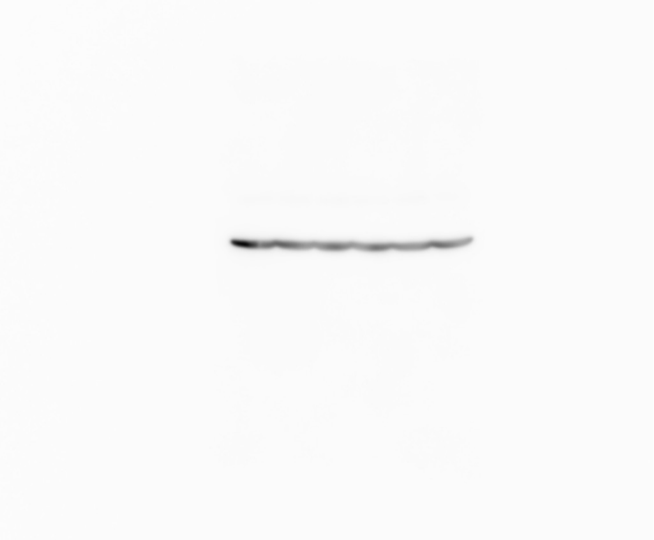

Figure S44: Original blots (3) in Figure 9C,D

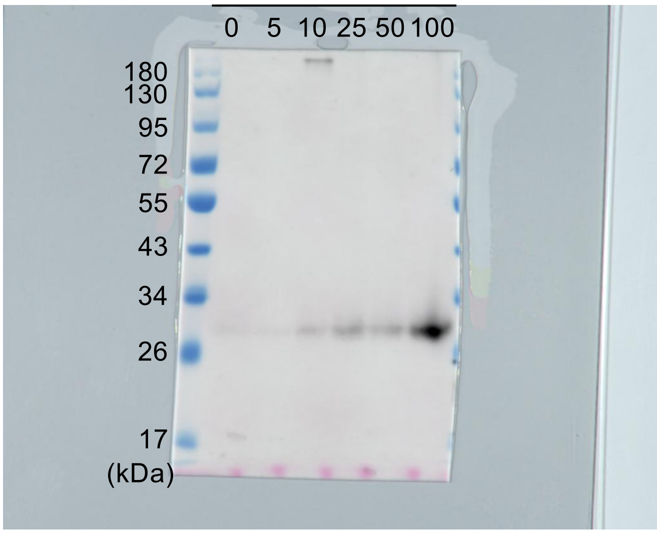

WB: TNF-R1

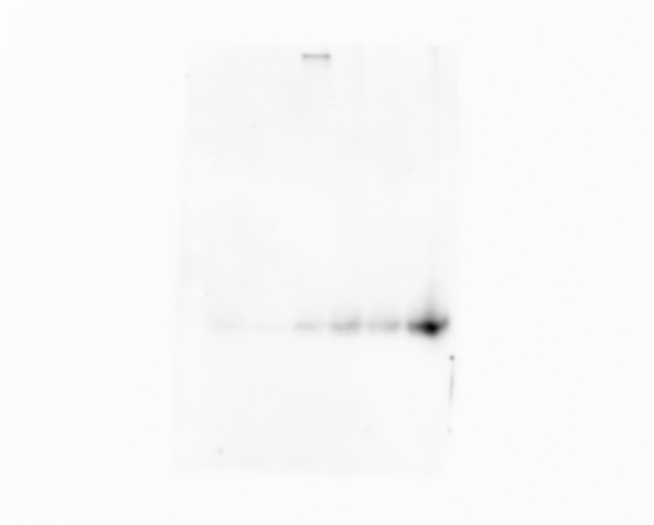

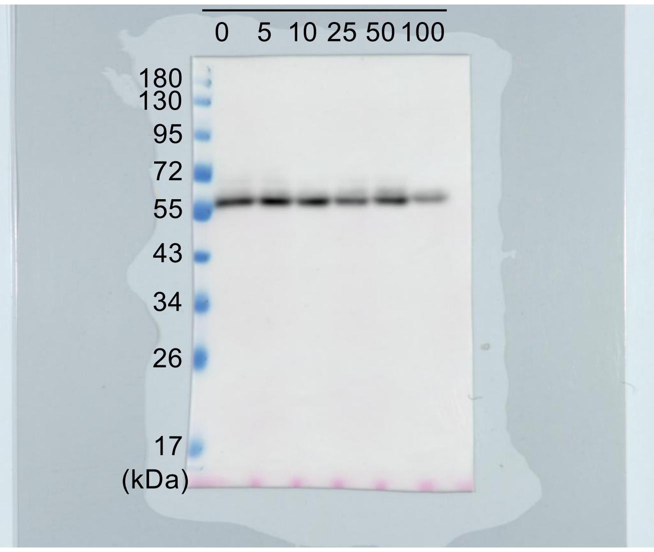

WB: TNF-R1

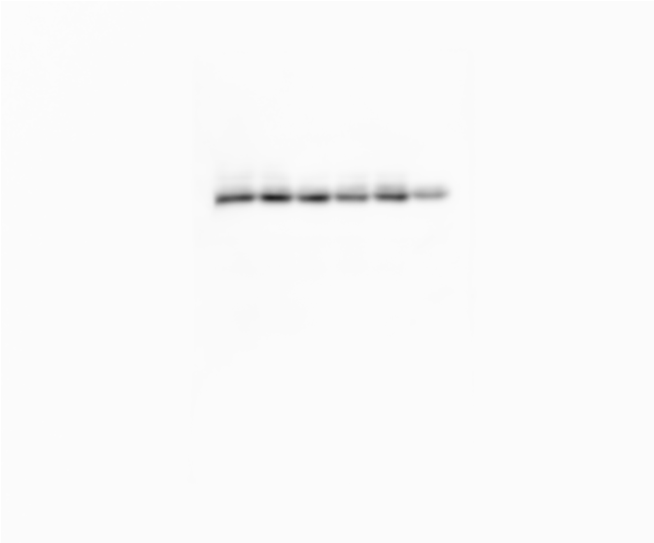

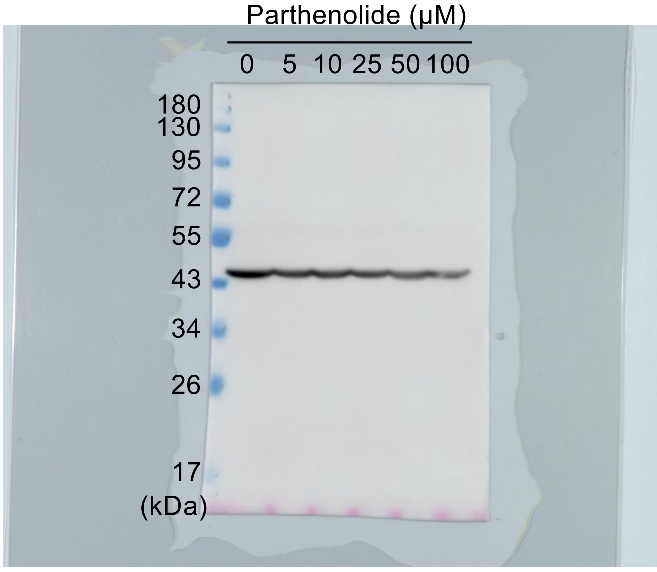

WB: β-Actin (reprobed)

Figure S45: Original blots in Figure 9E

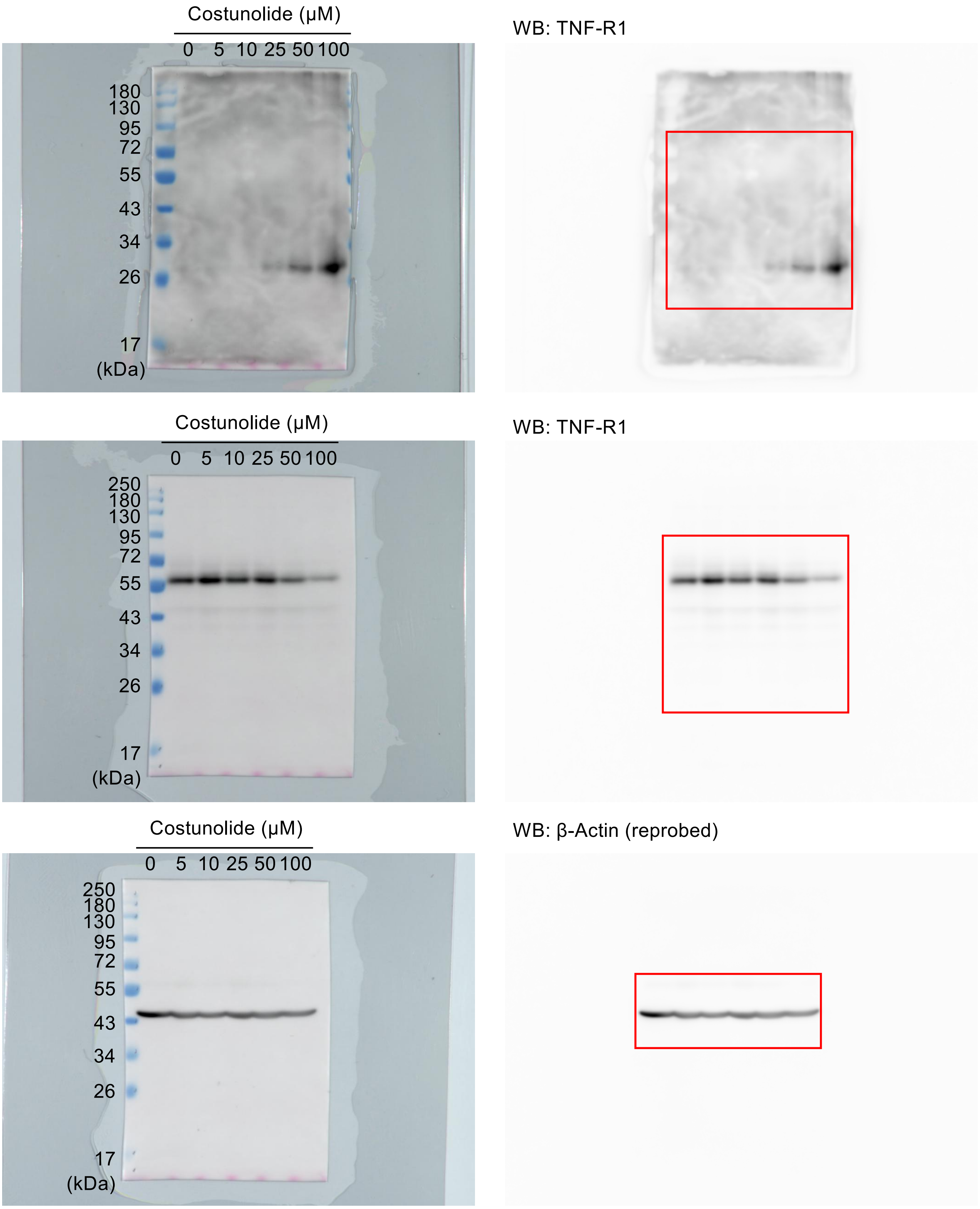

Figure S46: Original blots (1) in Figure 9F,G

Costunolide (μM)

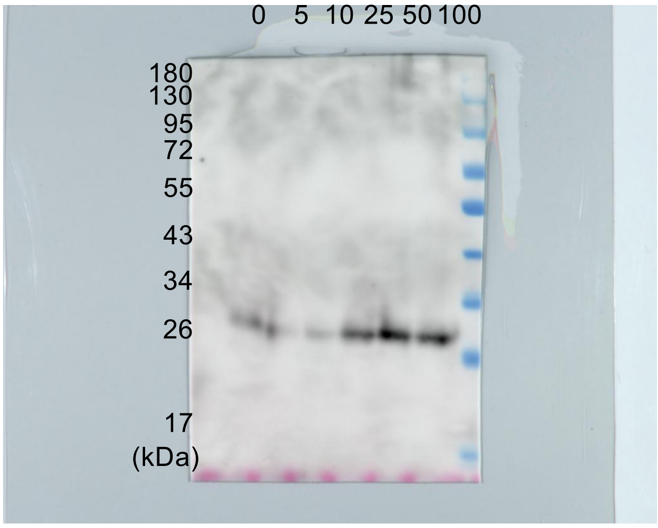

WB: TNF-R1

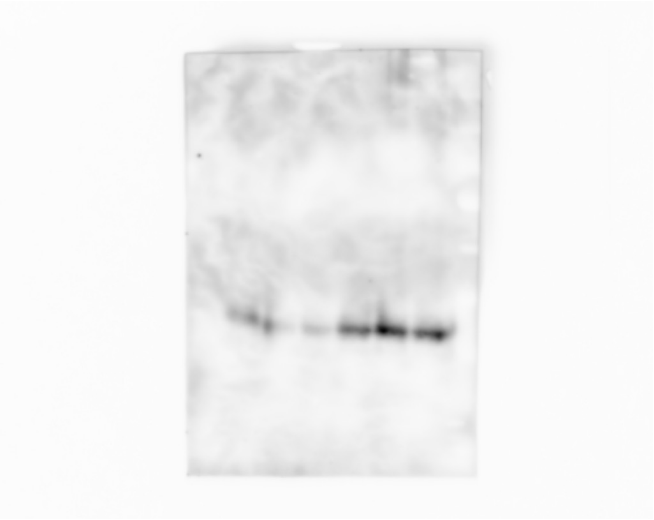

Costunolide (μM)

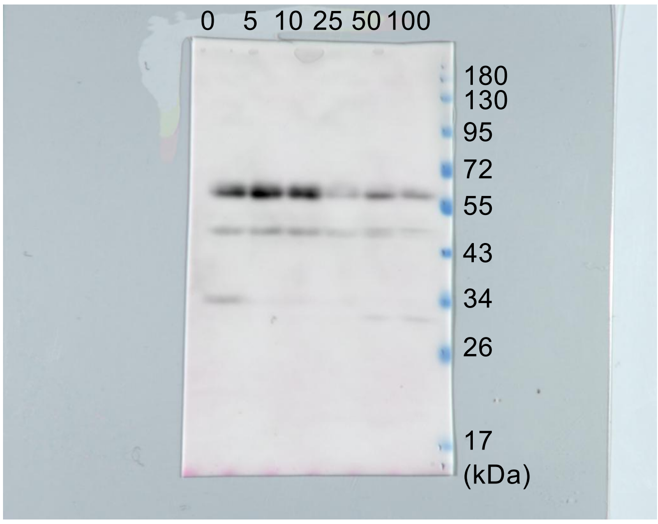

WB: TNF-R1

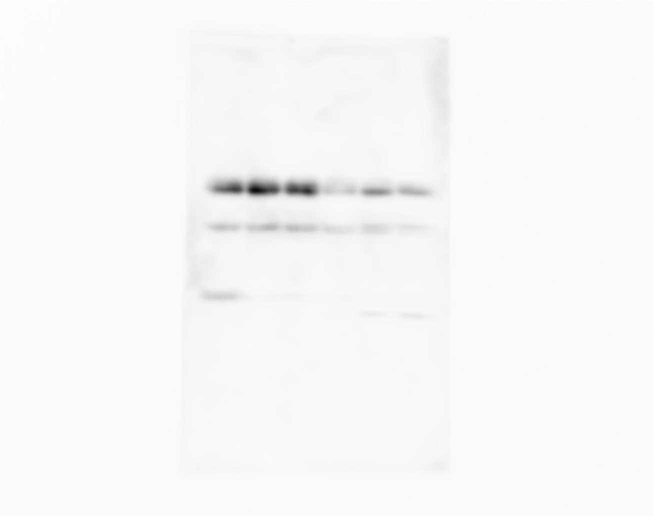

Costunolide (μM)

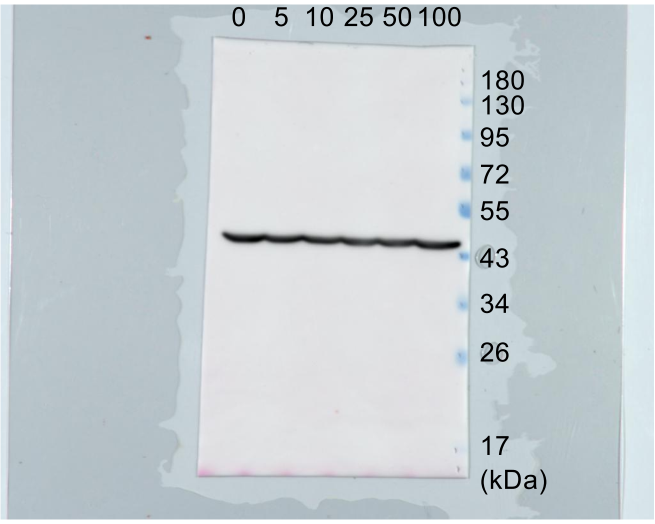

WB: β-Actin (reprobed)

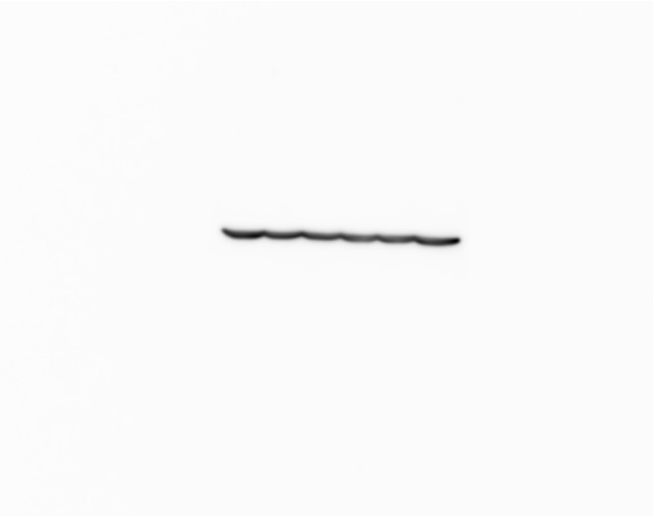

Figure S47: Original blots (2) in Figure 9F,G

Costunolide (µM)

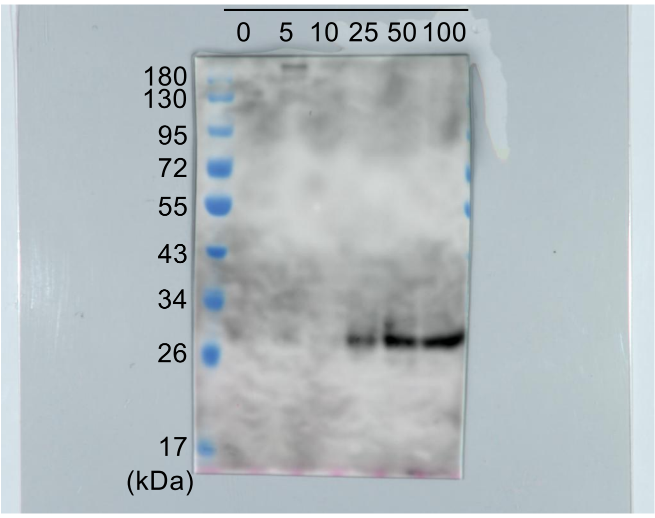

WB: TNF-R1

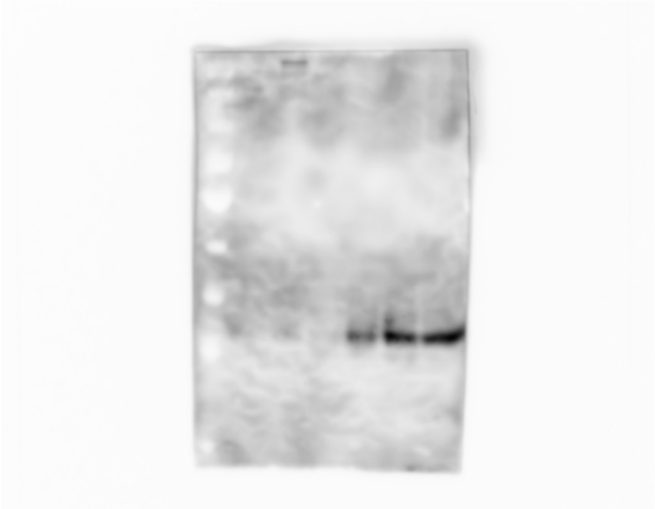

Costunolide (µM)

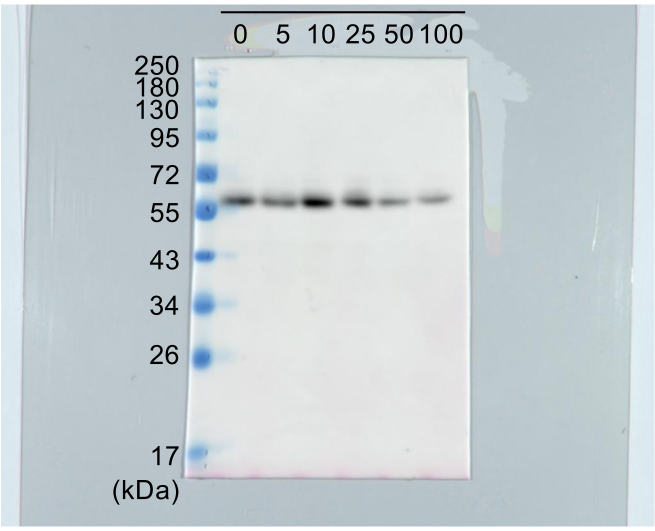

WB: TNF-R1

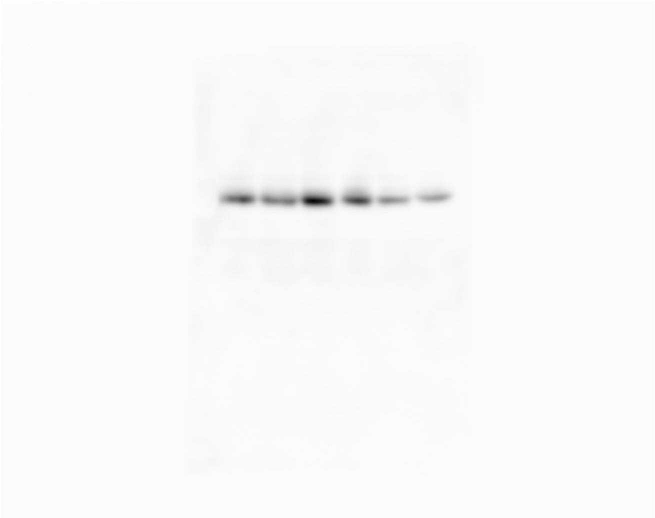

Costunolide (µM)

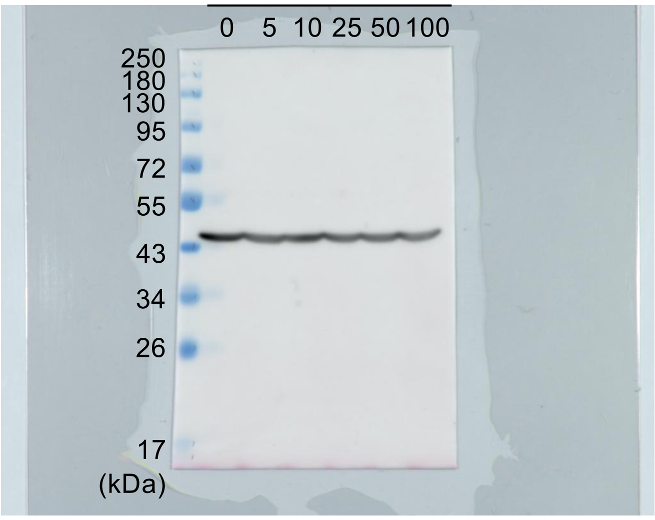

WB: β-Actin (reprobed)

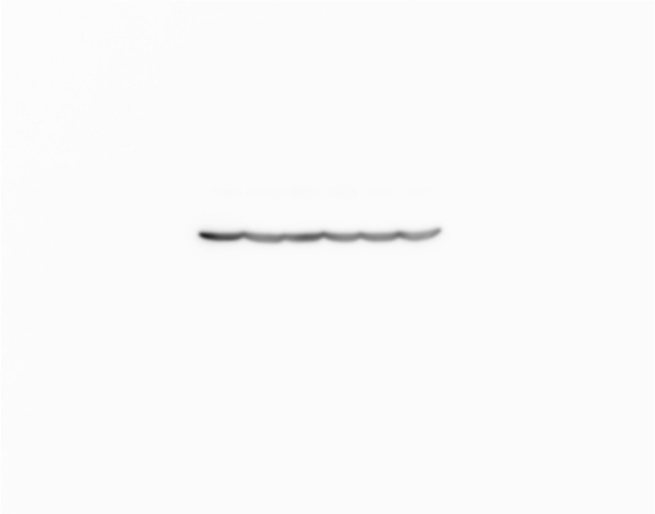

Figure S48: Original blots (3) in Figure 9F,G

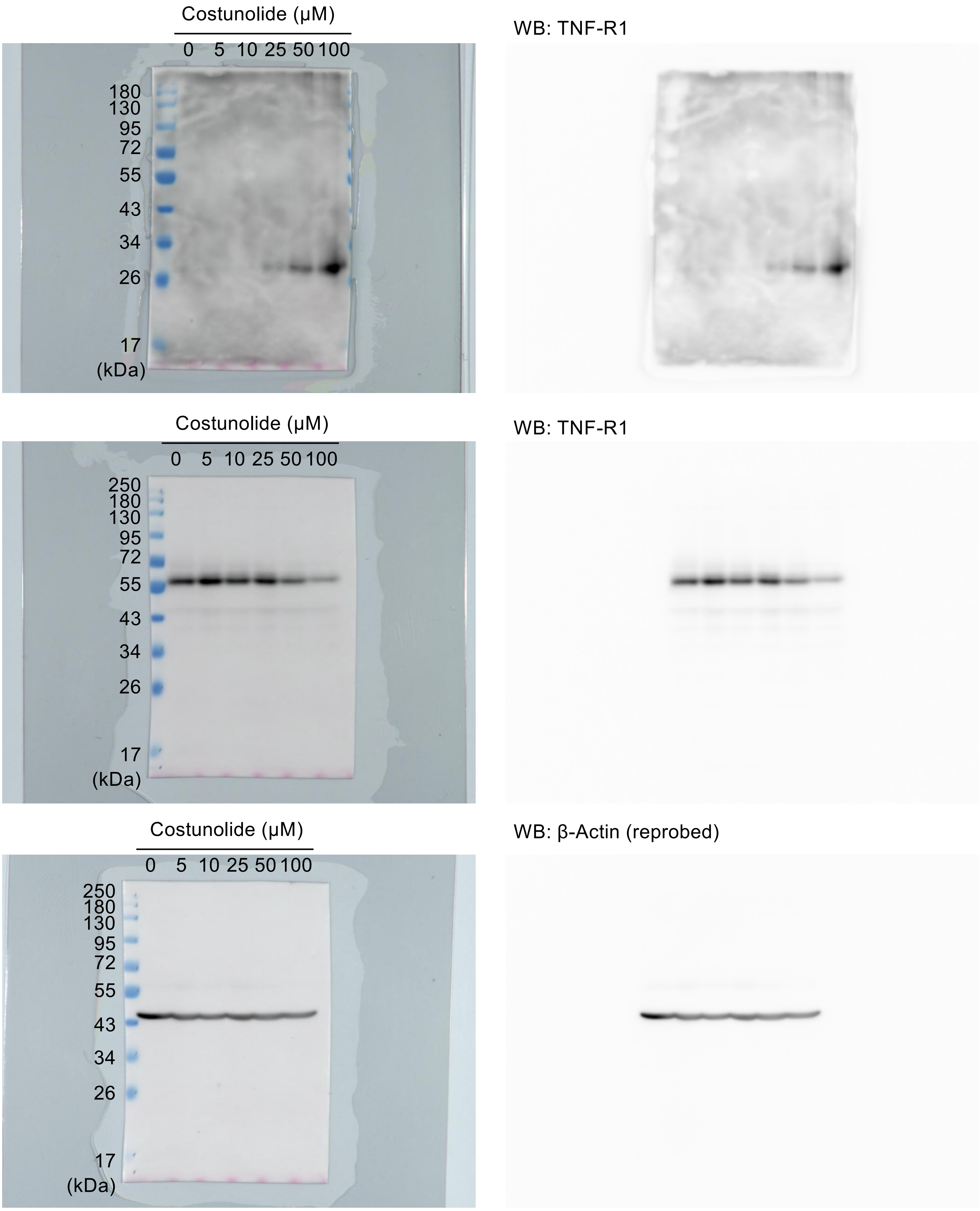

Supplement: Supplementary file 1 [file molecules-29-01866-s001.zip › molecules-2947077-supplementary.pdf]
